# Pd-Catalyzed Dynamic Kinetic Asymmetric Cross-Coupling of Heterobiaryl Bromides with *N*-Tosylhydrazones

**DOI:** 10.1021/acs.orglett.2c01355

**Published:** 2022-05-23

**Authors:** Shivashankar Kattela, Carlos Roque D. Correia, Abel Ros, Valentín Hornillos, Javier Iglesias-Sigüenza, Rosario Fernández, José M. Lassaletta

**Affiliations:** † Instituto de Investigaciones Químicas (CSIC-US) and Centro de Innovación en Química Avanzada (ORFEO-CINQA), Avda. Américo Vespucio, 49, 41092 Sevilla, Spain; ‡ Chemistry Institute, University of Campinas, CEP 13083-970 Campinas, Saõ Paulo, Brazil; § Departamento de Química Orgánica, Universidad de Sevilla and Centro de Innovación en Química Avanzada (ORFEO-CINQA), C/Prof. García González, 1, 41012 Sevilla, Spain

## Abstract

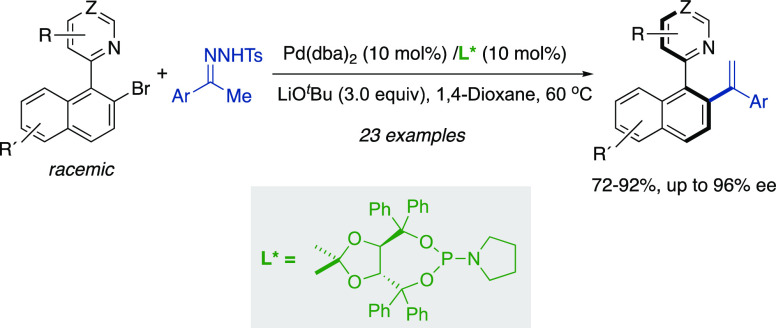

A dynamic kinetic asymmetric Pd-catalyzed cross-coupling reaction of heterobiaryl bromides with ketone *N*-tosylhydrazones for the synthesis of heterobiaryl styrenes is described. The combination of Pd(dba)_2_ as a precatalyst with a TADDOL-derived phosphoramidite ligand provides the corresponding coupling products in good yields and high enantioselectivities under mild conditions. Racemization-free *N*-oxidation and *N*-alkylation of the products allowed us to obtain appealing functionalized axially chiral heterobiaryl derivatives.

Axially chiral biaryl atropisomers are fundamentally important in nature due to their presence in a large number of natural products and bioactive substances.^1^ Moreover, they are also key structural frameworks in material sciences, supramolecular chemistry, and organic synthesis.^2^ Remarkably, an axially chiral (hetero)biaryl constitutes the central core of many privileged chiral ligands, catalysts, and auxiliaries that are routinely employed in asymmetric synthesis.^3^ Consequently, a great deal of effort has already been devoted to the efficient preparation^4^ of these chiral structures, including the asymmetric coupling of two aryl groups by oxidative dimerization or cross-coupling,^5^ asymmetric [2+2+2] cycloadditions,^6^ asymmetric ring opening of bridged biaryl lactones,^7^ stereoselective functionalization of prochiral biaryls, in particular by C–H functionalization,^8^ (dynamic) kinetic resolutions,^9^ and a growing number of organocatalytic approaches.^10^ Our group reported in 2013 an alternative methodology for the synthesis of heterobiaryls (e.g., 2-arylpyridines or analogues) consisting of Pd-catalyzed dynamic kinetic asymmetric (DYKAT) coupling between aryl boroxines and racemic heterobiaryl triflates.^11^ The resolution strategy is based on the formation of cationic oxidative addition diastereomeric intermediates (Scheme 1A) in which the configurational stability of the stereogenic axis is compromised by the widening of angles φ_1_ and φ_2_. This method was later extended to perform dynamic kinetic C–P,^12^ C–N,^13^ and other C–C cross-couplings^14^ from diverse heterobiaryl electrophiles. On the contrary, catalytic processes initiated by formation of metal carbenoids followed by migratory insertion have rarely been applied to the synthesis of axially chiral compounds. Inspired by the work of Barluenga and Valdés,^15^ the group of Gu reported on the use of 1-tetralone tosyl hydrazones as carbene precursors in the Pd-catalyzed coupling with substituted 1-naphthyl bromides, affording axially chiral vinyl arenes with large enantiomeric excesses (Scheme 1B).^16^ More recently, a related Cu-catalyzed coupling of diazo compounds with isoquinoline or phthalazine *N*-oxides has been reported to obtain axially chiral QUINOX analogues, although in racemic form (Scheme 1C).^17^ On the basis of the findings described above, we envisioned that the use of carbene precursors (e.g., hydrazones) as coupling partners in the DYKAT-based strategy should enable the synthesis of bifunctional heterobiaryl olefins via a palladium/carbene insertion, migration, and β-hydride elimination process (Scheme 1D). As a starting hypothesis, it was assumed that the low rotational barrier in carbenoid intermediate **I** increases significantly after the migratory insertion event as a result of the geometrical restrictions in the resulting intermediate **II**, a larger six-membered cycle with long N–Pd and Pd–C bonds.

**Scheme 1:**
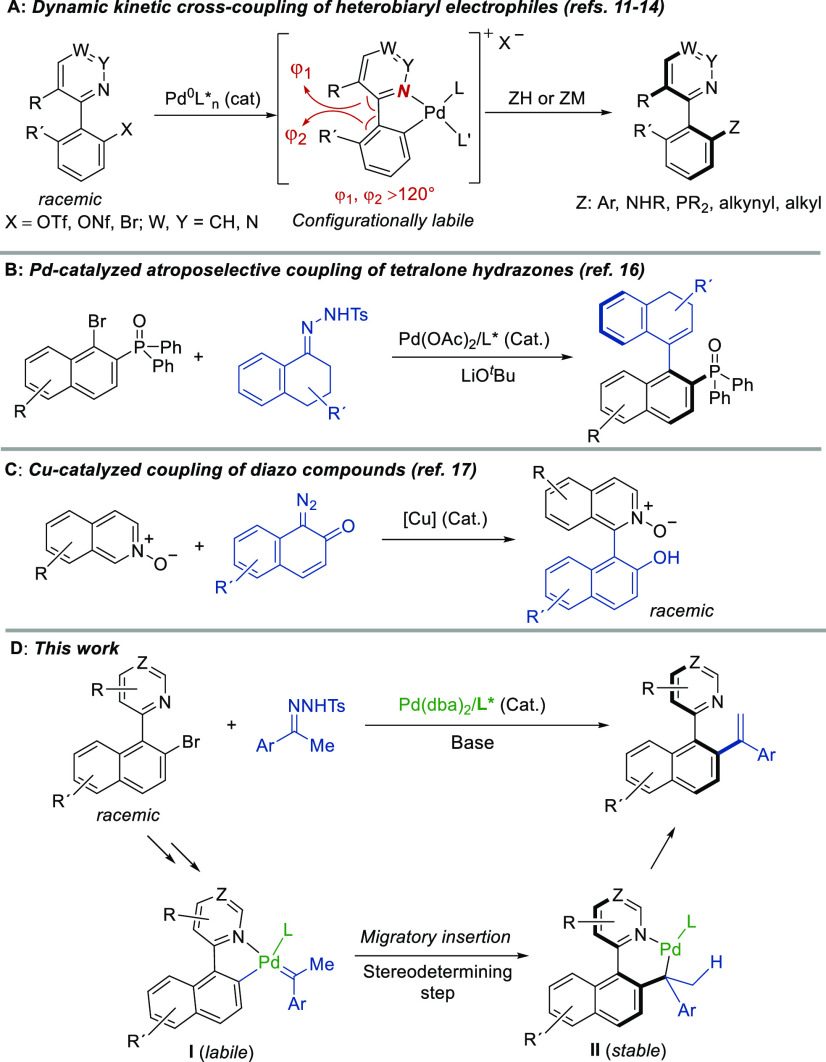
Antecedents and Our Synthetic Plan

The initial studies were carried out using the coupling between racemic bromide **1A** and acetophenone tosylhydrazone **2a** as the model reaction, with NaO^*t*^Bu as the base, anhydrous toluene as the solvent at 60 °C, 10 mol % Pd(OAc)_2_, and 12 mol % ligand as the catalyst system (Table 1). Different ligands that proved to be successful in our previous DYKAT processes were screened (see the Supporting Information for complete ligand screening). Bidentate P,P and P,N ligands such as BINAP **L1**, QUINAP **L2**, Josiphos-type **L3**, and *N,N*-pyridine-oxazoline ligand **L4** were not effective, and the desired product **3Aa** was obtained in a nearly racemic form (entries 1–4). These results can be explained by considering that bidentate ligands result in the formation of coordinatively saturated oxidative addition intermediates that, consequently, are not capable of forming key intermediate **I**. As expected, monodentate ligands such as TADDOL-based **L5–L10** and BINOL-derived **L11–L13** phosphoramidites showed in general better performance (entries 5–13). In particular, TADDOL derivative **L8**, containing a pyrrolidine moiety on the phosphoramidite, proved to be a promising ligand affording the desired (*R*)-**3Aa** product in good conversion (83%) and a moderate enantioselectivity (67%) (entry 8). After an additional screening of a Pd source, solvents, and a base (entries 14–21), we found that the use of Pd(dba)_2_ in combination with LiO^*t*^Bu as the base and anhydrous 1,4-dioxane as the solvent (entry 18) allowed the formation of (*R*)-**3Aa** with 85% conversion and 95% ee. Increasing the reaction temperature (65–70 °C) allowed full conversion to be reached, although at the expense of the enantioselectivity (entries 19 and 20). Finally, using a slightly larger excess of **2a** (1.5 equiv), the reaction also reaches full conversion while maintaining an excellent 95% ee (entry 21). Moreover, the amount of ligand could also be reduced to 10 mol % without erosion of the enantioselectivity or the catalytic activity (entry 22).

**Table 1:**
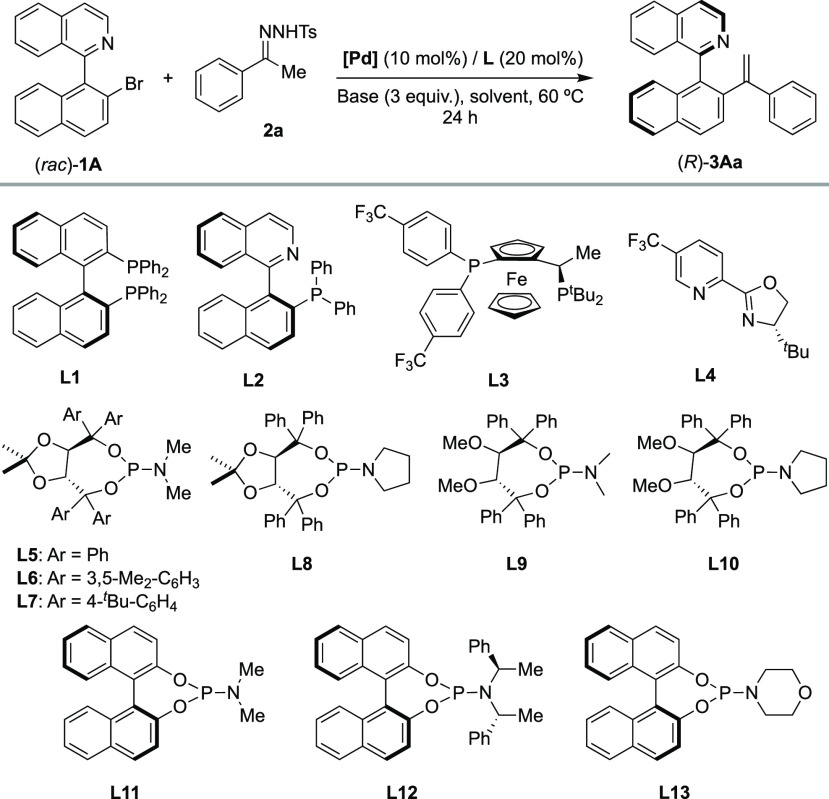
Screening of Ligands and Reaction Conditions^a^

	[Pd]	**L**	base	solvent	C (%)^b^	ee (%)^c^
1	Pd(OAc)_2_	**L1**	NaO*t*Bu	toluene	95	0
2	Pd(OAc)_2_	**L2**	NaO*t*Bu	toluene	22	3
3	Pd(OAc)_2_	**L3**	NaO*t*Bu	toluene	9	5
4	Pd(OAc)_2_	**L4**	NaO*t*Bu	toluene	32	0
5	Pd(OAc)_2_	**L5**	NaO*t*Bu	toluene	90	57
6	Pd(OAc)_2_	**L6**	NaO*t*Bu	toluene	72	21
7	Pd(OAc)_2_	**L7**	NaO*t*Bu	toluene	82	57
8	Pd(OAc)_2_	**L8**	NaO*t*Bu	toluene	83	67
9	Pd(OAc)_2_	**L9**	NaO*t*Bu	toluene	82	51
10	Pd(OAc)_2_	**L10**	NaO*t*Bu	toluene	58	51
11	Pd(OAc)_2_	**L11**	NaO*t*Bu	toluene	20	7
12	Pd(OAc)_2_	**L12**	NaO*t*Bu	toluene	24	9
13	Pd(OAc)_2_	**L13**	NaO*t*Bu	toluene	36	5
14	Pd(TFA)_2_	**L8**	NaO*t*Bu	toluene	85	67
15	Pd_2_(dba)_3_	**L8**	NaO*t*Bu	toluene	48	70
16	Pd(dba)_2_	**L8**	NaO*t*Bu	toluene	76	70
17	Pd(dba)_2_	**L8**	LiO*t*Bu	toluene	82	92
18	Pd(dba)_2_	**L8**	LiO*t*Bu	dioxane	85	95
19^d^	Pd(dba)_2_	**L8**	LiO*t*Bu	dioxane	>99	89
20^e^	Pd(dba)_2_	**L8**	LiO*t*Bu	dioxane	>99	91
21^f^	Pd(dba)_2_	**L8**	LiO*t*Bu	dioxane	>99	95
22^g^	Pd(dba)_2_	**L8**	LiO*t*Bu	dioxane	>99	95

a Reaction conditions: 0.1 mmol of **1A** in an anhydrous solvent (1.2 mL), **2a** (0.12 mmol, 1.2 equiv), and 3 equiv of base.

b Conversions were determined by ^1^H NMR spectroscopy.

c The ee values were determined by HPLC on chiral stationary phases.

d Reaction carried out at 70 °C.

e Reaction carried out at 65 °C.

f With 0.15 mmol (1.5 equiv) of **2a**.

g Reaction performed with 10 mol % ligand.

The coupling reaction of bromide **1A** could also be extended to other aromatic tosylhydrazones (Scheme 2). The reaction tolerates hydrazones **2b–d** containing electron-donating (OMe and Me) or slightly electron-withdrawing (Cl) groups in the *para* position, affording products **3Ab–d** in excellent yields and enantioselectivities of ≤96% ee. Additionally, the reaction also tolerates substrates containing different groups (F, OMe, and Me) in the *ortho* (**2e**), *meta* (**2g**), and *ortho, meta* (**2f**) positions, affording the desired products (*R*)-**3Ae–g** in excellent yields and excellent enantioselectivities (89–93% ee). A 1.5 mmol scale reaction (0.5 g) of *rac*-**1A** and **2a** was performed, affording (*R*)-**3Aa** in a similar 82% yield and 95% ee.

**Scheme 2:**
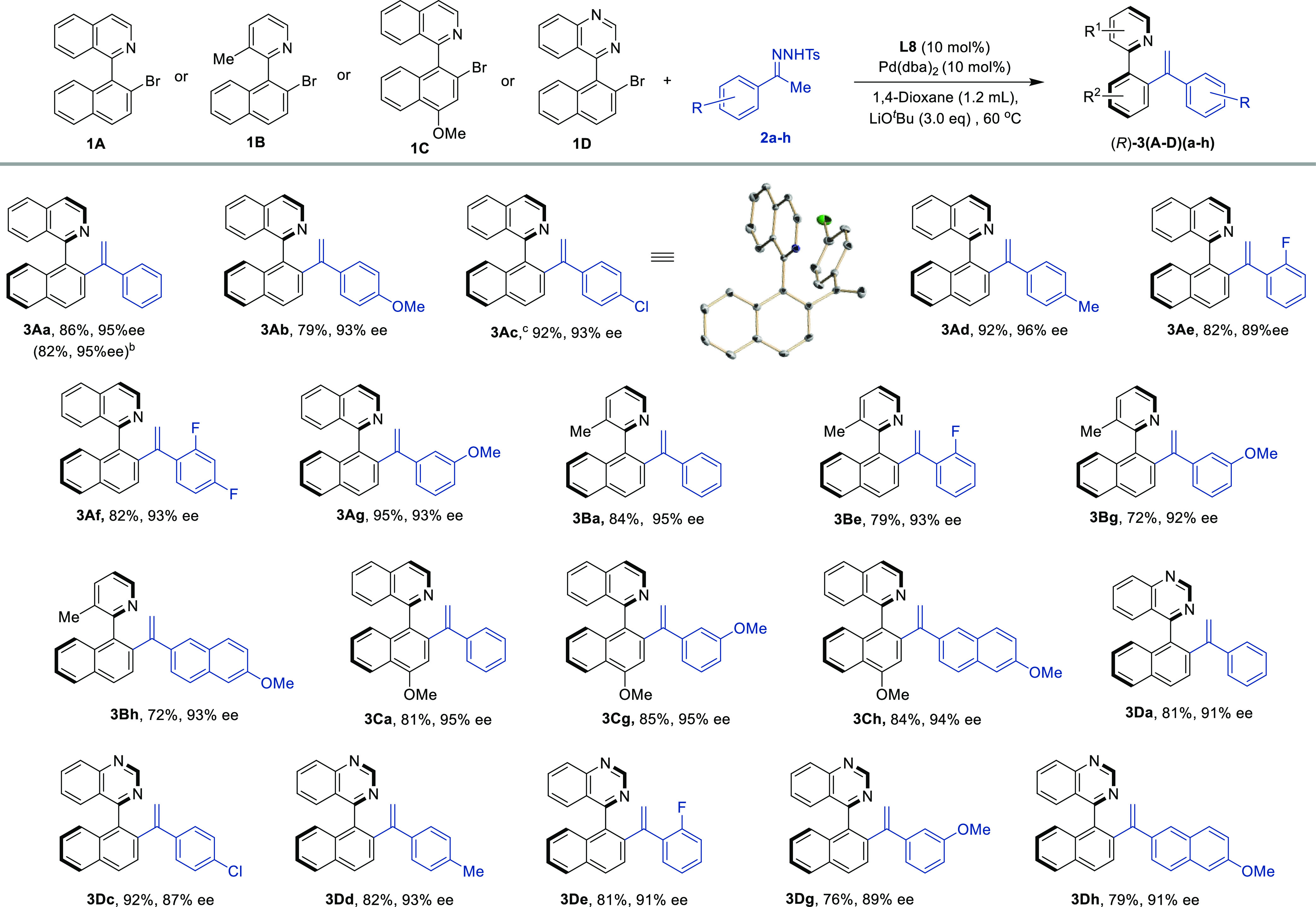
Scope of Hydrazones and Heterobiaryls Reaction conditions: 0.1 mmol of **1A**–**D** in anhydrous 1,4-dioxane (1.2 mL), **2a–k** (0.15 mmol, 1.5 equiv), and 3 equiv of LiO*t*Bu for 24 h at 60 °C. Yields given for isolated products after chromatographic purification. The ee values were determined by HPLC on chiral stationary phases. Reaction performed on a 1.5 mmol (536 mg) scale. Absolute configuration determined by X-ray single-crystal analysis.

Next, we examined the scope of other heterobiaryl bromides **1B–D**. Their reactivity followed a similar pattern. Different naphthyl picoline **1B**, isoquinoline **1C**, and quinazoline **1D** derivatives could be coupled with the model acetophenone tosylhydrazone **2a** and with derivatives **2c–h** containing substituents in the *ortho*, *meta*, or *para* positions to afford the desired products (*R*)-**3B–D** in excellent yields and enantioselectivities of >90% in most cases. The absolute configuration of product (*R*)-**3Ac** could be unambiguously assigned by X-ray diffraction analysis. The absolute configuration of other products (*R*)-**3A–D** was assigned by analogy assuming a uniform reaction pathway.

The nitrogen atom of the isoquinoline unit maintains its reactivity and can be used in quaternization reactions such as *N*-oxide formation with *m*-CPBA (→**4Aa**) and *N*-alkylation with BnBr (→**5Aa**) to yield interesting functionalized products for applications in asymmetric catalysis (Scheme 3).

**Scheme 3:**
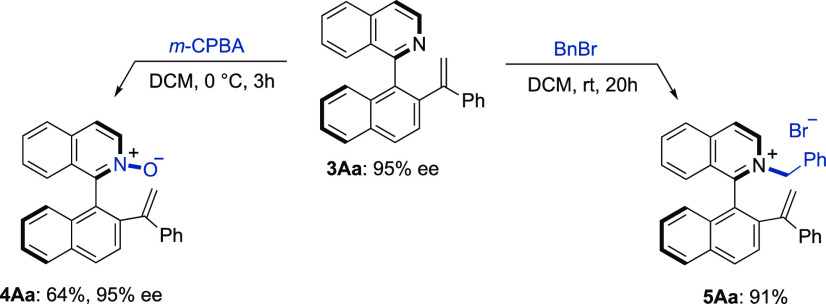
Representative Derivatizations

In summary, we have developed a highly efficient methodology for the synthesis of axially chiral heterobiaryl styrenes based on a dynamic kinetic asymmetric coupling between readily available racemic heterobiaryl bromides and tosyl hydrazones. A broad scope, functional group tolerance, and excellent enantiomeric excesses were obtained using a chiral Pd(dba)_2_/TADDOL-derived phosphoramidite catalytic system.

## Supporting Information Available

Experimental details, spectroscopic and analytical data for new compounds, and HPLC traces (PDF)

## Accession Codes

CCDC 2165277 contains the supplementary crystallographic data for this paper. These data can be obtained free of charge via www.ccdc.cam.ac.uk/data_request/cif, or by emailing data_request@ccdc.cam.ac.uk, or by contacting The Cambridge Crystallographic Data Centre, 12 Union Road, Cambridge CB2 1EZ, UK; fax: +44 1223 336033.
