# Supplementary material for: Pd-Catalyzed Dynamic Kinetic Asymmetric Cross-Coupling of Heterobiaryl Bromides with N-Tosylhydrazones
Source: Org Lett. 2022 May 23;24(21):3812–6. doi: 10.1021/acs.orglett.2c01355 (PMC9490869; doi:10.1021/acs.orglett.2c01355)
Supplement: Supplementary file 1 — ol2c01355_si_001.pdf [file ol2c01355_si_001.pdf]

Supporting Information

Pd-Catalyzed Dynamic Kinetic Asymmetric Cross-Coupling of Heterobiaryl Bromides with *N*-Tosylhydrazones

Shivashankar Kattela,[§] Carlos Roque D. Correia,[¶] Abel Ros,[ʃ,§] Valentín Hornillos[ʃ,§].Javier Iglesias-Sigüenza,[ʃ] Rosario Fernández,*[,ʃ] José M. Lassaletta*[,§]

§ Instituto de Investigaciones Químicas (CSIC-US) and Centro de Innovación en Química Avanzada (ORFEO-CINQA), Avda. Américo Vespucio, 49, 41092 Sevilla, Spain. E-mail: jmlassa@iiq.csic.es

¶ Chemistry Institute, University of Campinas, CEP 13083-970 Campinas, São Paulo, Brazil.

ʃ Departamento de Química Orgánica, Universidad de Sevilla and Centro de Innovación en Química Avanzada (ORFEO-CINQA), C/ Prof. García González, 1, 41012 Sevilla, Spain. E.mail: ffernan@us.es

1. General Information. **S2**

2. Ligands screening (Table S1). **S3**

3. Synthesis of 1D **S4**

4. General procedure and characterization data for compounds 3. **S4**

5. Product transformations. **S12**

6. NMR spectra and HPLC traces. **S14**

7. X-Ray Crystallographic Data. **S61**

1. General Information.

^1H NMR and ^{13}C NMR spectra were recorded at 400 MHz and 100 MHz respectively, using a Bruker DRX-400 spectrometer and $CDCl_3$ as the solvent. Spectra were referenced using the residual protio solvent peaks as internal standard (7.26 ppm for ^1H NMR and 77.0 ppm for ^{13}C NMR experiments). Column chromatography was performed on silica gel (Merck Kieselgel 60). Analytical TLC was performed on aluminum backed plates (1.5 × 5 cm) pre-coated (0.25 mm) with silica gel (Merck, Silica Gel 60 F_{254}). Compounds were visualized by exposure to UV light or by dipping the plates in a solution of 5% $(NH_4)_2Mo_7O_{24}\cdot 4\ H_2O$ in 95% EtOH (w/v) or followed by heating. Enantiomeric excesses were determined by HPLC using chiral columns (Chiralpak® IA) and *n*-Hexane/isopropanol mixtures as eluent.

All chemical reactions were carried out in oven-dried Schlenk tubes under argon atmosphere employing standard techniques. Anhydrous Toluene was obtained using Grubbs-type solvent drying columns, whereas anhydrous 1,4-dioxane was obtained by distilling from sodium/benzophenone under N_2 atmosphere. Ligands **L1-L3**, **L11** and $Pd(OAc)_2$ were purchased from Aldrich and TCI respectively. Phosphoramites **L5-L10, L12-L13, L16-L20**[1] and Oxazoline ligands **L4,L14-L15**[2] were synthesized following procedure described in the literature. Bromide substrates **1A-C**[3] and tosylhydrazones **2a-k**[4] were prepared following procedures described in the literature.

Crystals of suitable size were covered with FOMBLIN oil and mounted on a glass fiber. Data collection has been performed on a Bruker SMART APEX II CCD area detector on a D8 goniometer at 100 K, using a graphite monochromator Cu Kα1 (λ = 1.54178 Å) and a Bruker Cryo-Flex low-temperature device. Data collection was processed with APEX-W2D-NT,[5] cell refinement and data reduction with SAINT-Plus1 and the absorption was corrected by multiscan method applied by SADABS.[6] The structure was solved by direct method and refined on F2 (SHELXTL).[7] Non-hydrogen atoms were refined with anisotropic displacement parameters and hydrogen atoms attached to refined atoms were placed in geometrically idealized positions and refined by using a riding model.

---

[1] a) Alexakis, A.; Burton, J.; Vastra, J.; Benhaim, C.; Fournioux, X.; van den Heuvel, A.; Levêque, J.-M.; Mazeé, F.; Rosset, S. *Eur. J. Org. Chem.* **2000**, 4011-4027. b) Teller, H.; Flügge, S.; Goddard, R.; Fürstner, A. *Angew. Chem. Int. Ed.* **2010**, *49,* 1949-1953.

[2] Khan, I. U.; Kattela, S.; Hassan, A.; Correia, C. R. D. *Org. Biomol. Chem.,* **2016,** *14*, 9476-9480.

[3] a) Pais, V. F.; Alcaide, M. M.; López-Rodríguez, R.; Collado, D.; Nájera, F.; Pérez Inestrosa, E.; Álvarez, E.; Lassaletta, J. M.; Fernández, R.; Ros, A.; Pischel, U. *Chem. Eur. J.* **2015**, *21*, 15369. b) Ramírez-López, P.; Ros, A.; Romero- Arenas, A.; Iglesias-Sigüenza, J.; Fernández, R.; Lassaletta, J. M. *J. Am. Chem. Soc*. **2016**, *138*, 12053.

[4] Feng, J.; Li, B.; He, Y.; Gu, Z. *Angew. Chem. Int. Ed.* **2016**, *55,* 2186-2190.

[5] APEX2 (version 2009.11_0). Program for Bruker CCD X-Ray Diffractometer Control, Bruker AXS Inc., Madison, WI, **2009**.

[6] SADABS, Bruker (2006). APEX 2. Version 2.1. Bruker Analytical X-Ray Solutions, Madison, Wisconsin, USA.

[7] G. M. Sheldrick, SHELXTL, version 6.14. Program for solution and refinement of crystal structures, Universität Göttingen, Germany, **2000**.

2. Ligands screening

Table S1: Full screening of ligands.[a]

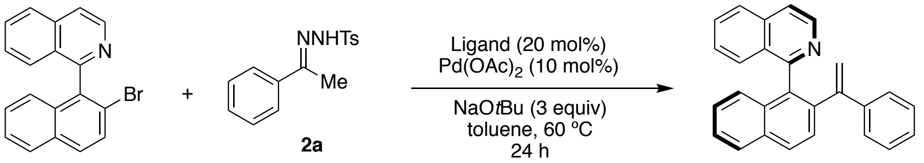

(±)-1A (*R*)-**3Aa**

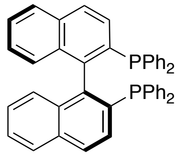

L1, >95% conv, 0% ee

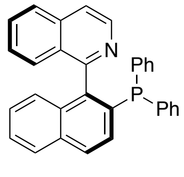

L2, 22% conv, 3% ee

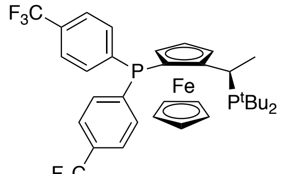

L3, 9% conv, 5% ee

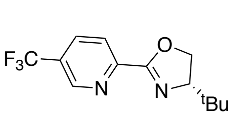

L4, 32% conv, 0% ee

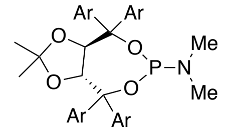

L5, Ar= Ph, 90% conv, 57% ee
L6, Ar= 3,5-Me-C_6H_3, 72% conv, 21% ee
L7, Ar= 4-^{t}Bu-C_6H_4, 82% conv, 57% ee

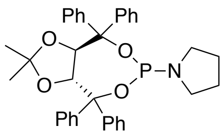

L8, 83% conv, 67% ee

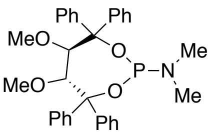

L9, 82% conv, 51% ee

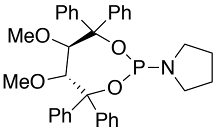

L10, 58% conv, 51% ee

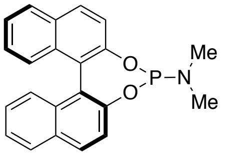

L11, 20% conv, 7% ee

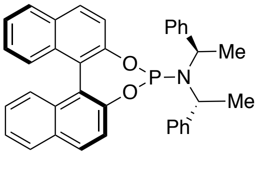

L12, 24% conv, 9% ee

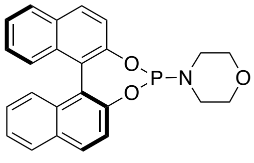

L13, 36% conv, 5% ee

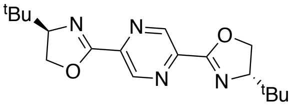

L14, 38% conv, 0% ee

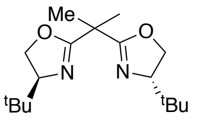

L15, 15% conv, 0% ee

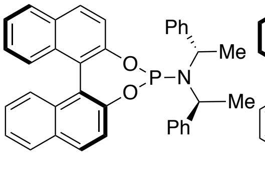

L16, 6% conv, 0% ee

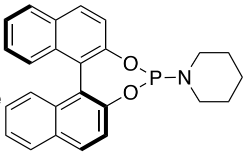

L17, 15% conv, 5% ee

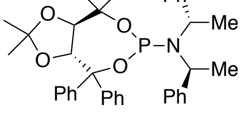

L18, 25% conv, 0% ee

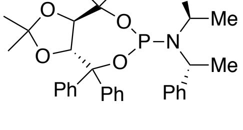

L19, 25% conv, 0% ee

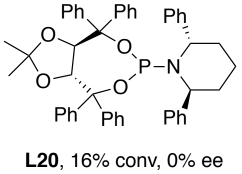

L20, 16% conv, 0% ee

[a]**Reactions conditions**: 0.1 mmol **1A** in anhydrous toluene (1.2 mL), **2a** (0.12 mmol, 1.2 equiv) and 3 equiv of NaO*t*Bu. Ee's were determined by chiral HPLC analysis and conversions by ^{1}H-NMR spectroscopy.

3. Synthesis of 1D.

4-(2-bromonaphthalen-1-yl) quinazoline (1D).

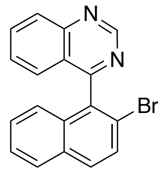

Following a reported procedure,[3] starting from 4-(naphthalen-1-yl)quinazoline (2 mmol, 512 mg), and after purification by column chromatography (*n*-Hexane/EtOAc, 9:1 to 8:2), **1D** (581 mg, 87%) was obtained as a white amorphous solid. **^{1}H NMR (400 MHz, $CDCl_3$)**: δ 9.58 (s, 1H), 8.26 (d, J = 8.5 Hz, 1H), 8.03 – 7.90 (m, 3H), 7.82 (d, J = 8.8 Hz, 1H), 7.55 (ddd, J = 8.1, 5.3, 1.2 Hz, 2H), 7.51 – 7.44 (m, 1H), 7.42 – 7.35 (m, 1H), 7.07 (d, J = 8.5 Hz, 1H). **^{13}C NMR (100 MHz, $CDCl_3$):** δ 168.3, 155.0, 150.6, 134.4, 134.3, 133.1, 132.2, 130.8, 129.8, 128.9, 128.3, 127.7, 126.6, 126.6, 125.4, 124.5, 120.8. **HRMS (ESI)**: calcd. for $C_{18}H_{12}N_2Br$ (M + H^+) 337.0157. Found 337.0158.

4. General procedure and characterization data for compounds 3.

A flamed-dried Schlenk tube was charged with the corresponding bromide **1A-D** (0.1 mmol, 1.0 equiv), hydrazone **2a-j** (0.15 mmol, 1.5 equiv), $Pd(dba)_2$ (0.1 mmol, 10 mol%), **L8** (0.1 mmol, 10 mol%), ^{t}BuOLi (0.3 mmol, 3.0 equiv). After three cycles of vacuum-nitrogen, anhydrous 1,4-dioxane (1.2 mL) were added to a Schlenk tube under nitrogen atmosphere and the resulting mixture was stirred at 60 °C (oil bath) for 24 h. After being cooled to room temperature, water (2 mL) was added and the resulting mixture was extracted with DCM (10 mL). The organic layer was dried over anhydrous Na_2SO_4, filtered, concentrated, and the residue was purified by column chromatography on silica gel using Hexane/AcOEt mixtures.

For the preparation of the racemic compounds, similar procedure was follow but using (±) BiNAP (20 mol%) and ^{t}BuONa (0.2 mmol, 2.0 equiv) as the ligand and the base. In these cases, the reactions were carried at 80 °C (oil bath) for 16h**.** Yields and characterization data for compounds **3** is as follows:

(*R*)-1-(2-(1-phenylvinyl) naphthalen-1-yl) isoquinoline (3Aa).

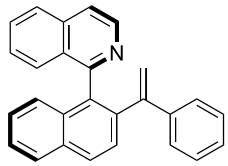

Following the general procedure, purification by column chromatography (*n*-Hexane/EtOAc, 9:1 to 8:2) afforded **3Aa** (31 mg, 86%) as a colorless viscous oil. $[\alpha]^{20}_D$ +116.6 (*c* 1.5, $CHCl_3$) for 95 % *ee*. **^{1}H NMR (400 MHz, $CDCl_3$):** δ 8.52 (d, J = 5.6 Hz, 1H), 8.05 (d, J = 8.4 Hz, 1H), 7.99 (d, J = 8.2 Hz, 1H), 7.79 (d, J = 8.2 Hz, 1H), 7.67 – 7.55 (m, 3H), 7.50 (t, J = 7.8 Hz, 2H), 7.35 – 7.27 (m, 2H), 7.20 (d, J = 8.4 Hz, 1H), 7.01 (s, 5H), 5.36 (s, 1H), 5.24 (s, 1H). **^{13}C NMR (100 MHz, $CDCl_3$)**: δ 159.6, 149.3, 141.8, 141.1, 139.6, 135.9, 134.8, 133.0, 132.8, 130.0, 128.62, 128.58, 128.3, 128.0, 127.9, 127.7, 127.6, 127.2, 127.0, 126.8, 126.63, 126.58,

126.5, 125.9, 119.9, 117.5. **HRMS (ESI)** calcd. for $C_{27}H_{19}N$ (M + H^+) 358.1590. Found 358.1593. **HPLC** (IA column, Isopropanol/n-hexane = 10/90, flow rate = 1.0 mL/min, T= 30°C, l = 254 nm) t_R = 5.74 min (major), 8.62 min (minor).

Note: When the reaction was carried out at 1.5 mmol scale, *(Ra)*-**3AA** was obtained with 82% yield (439 mg) and 95% ee.

(*R*)-1-(2-(1-(4-methoxyphenyl)vinyl) naphthalen-1-yl) isoquinoline (3Ab).

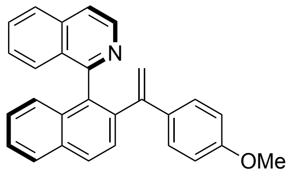

Following the general procedure, purification by column chromatography (*n*-Hexane/EtOAc, 4:1 to 7:3) afforded **3Ab** (31 mg, 79%) as a colorless viscous oil**.** $[\alpha]^{20}_D$ +175.0 (*c* 0.6, $CHCl_3$) for 93 % *ee*. **^{1}H NMR (400 MHz, $CDCl_3$)**: *δ* 8.48 (d, *J* = 5.6 Hz, 1H), 7.99 (d, *J* = 8.5 Hz, 1H), 7.93 (d, *J* = 8.2 Hz, 1H), 7.77 (d, *J* = 8.2 Hz, 1H), 7.58 – 7.52 (m, 3H), 7.47-7.42 (m, 2H), 7.30 – 7.26 (m, 2H), 7.13 (d, *J* = 8.5 Hz, 1H), 6.88 (d, *J* = 8.3 Hz, 2H), 6.51 (d, *J* = 8.3 Hz, 2H), 5.21 (s, 1H), 5.05 (s, 1H), 3.67 (s, 3H). **^{13}C NMR (101 MHz, $CDCl_3$)**: *δ* 159.8, 158.6, 148.7, 142.1, 139.9, 135.8, 135.0, 133.9, 133.0, 132.8, 129.8, 128.7, 128.4 (3 x C), 128.2, 127.90, 127.88, 126.63, 126.56, 126.5, 127.0, 125.8, 119.8, 115.8, 113.0 (2 x C), 55.2. **HRMS (ESI**) calcd. for $C_{28}H_{21}ON$ (M + H^+) 388.1696. Found 388.1693. **HPLC** (IA column, Isopropanol/n-hexane = 10/90, flow rate = 1.0 mL/min, T= 30°C, l = 254 nm) t_R = 7.88 min (major), 12.14 min (minor).

(*R*)-1-(2-(1-(4-chlorophenyl)vinyl) naphthalen-1-yl) isoquinoline (3Ac).

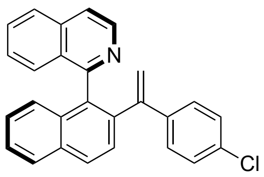

Following the general procedure, purification by column chromatography (*n*-Hexane/EtOAc, 9:1 to 4:1) afforded **3Ac** (36 mg, 92%) as a white amorphous solid**.** $[\alpha]^{20}_D$ +91.1 (*c* 0.7, $CHCl_3$) for 93 % *ee.* **^{1}H NMR (400 MHz, $CDCl_3$)**: *δ* 8.50 (d, *J* = 5.6 Hz, 1H), 8.04 (d, *J* = 8.5 Hz, 1H), 7.98 (d, *J* = 8.2 Hz, 1H), 7.80 (d, *J* = 8.2 Hz, 1H), 7.66 – 7.55 (m, 3H), 7.50 (t, *J* = 7.4 Hz, 1H), 7.42 (d, *J* = 8.4 Hz, 1H), 7.32 (d, *J* = 7.9 Hz, 2H), 7.19 (d, *J* = 8.5 Hz, 1H), 6.91 (d, *J* = 8.2 Hz, 2H), 6.84 (d, *J* = 8.2 Hz, 2H), 5.31 (s, 1H), 5.25 (s, 1H). **^{13}C NMR (101 MHz, $CDCl_3$)**: *δ* 159.4, 148.5, 141.9, 139.6, 139.2, 135.9, 134.9, 133.1, 132.7, 130.0, 128.7, 128.5, 128.4, 128.1, 128.0, 127.8, 127.6, 126.9, 126.7, 126.4, 126.1, 120.0, 117.8. **HRMS (ESI)** calcd. for $C_{27}H_{18}NCl$ (M + H^+) 392.1201. Found 392.1197. **HPLC** (IA column, Isopropanol/n-hexane = 10/90, flow rate = 1.0 mL/min, T= 30°C, l = 254 nm) t_R = 6.34 min (major), 9.23min (minor). X-ray quality crystals were obtained by slow evaporation of a solution of **3Ac** in hexane.

(*R*)-1-(2-(1-(*p*-tolyl)vinyl) naphthalen-1-yl) isoquinoline (3Ad).

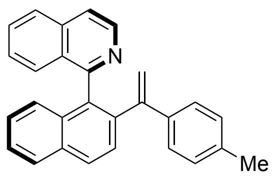

Following the general procedure, purification by column chromatography (*n*-Hexane/EtOAc, 9:1 to 4:1) afforded **3Ad** (34 mg, 92%) as a light yellow viscous oil**.** $[\alpha]^{20}_D$ +80.3 (*c* 0.4, $CHCl_3$) for 96 % *ee*. **^{1}H NMR (400 MHz, $CDCl_3$)**: *δ* 8.53 (d, *J* = 5.4 Hz, 1H), 7.99 (dd, *J* = 19.2, 8.3 Hz, 2H), 7.79 (d, *J* = 8.1 Hz, 1H), 7.59 (t, *J* = 6.9 Hz, 3H), 7.49 (t, *J* = 7.3 Hz, 2H), 7.36 – 7.27 (m, 2H), 7.17 (d, *J* = 8.4 Hz, 1H), 6.91 (d, *J* = 7.6 Hz, 2H), 6.82 (d, *J* = 7.7 Hz, 2H), 5.30 (s, 1H), 5.14 (s, 1H), 2.20 (s, 3H). **^{13}C NMR (101 MHz, $CDCl_3$)**: δ 159.7, 148.9, 141.8, 139.9, 138.4, 136.7, 135.9, 134.8, 132.9, 132.8, 129.9, 129.3, 129.1, 128.7, 128.5, 128.34, 128.29, 128.0, 127.2, 126.8, 126.6, 126.52, 126.49, 125.9, 120.0, 116.7, 21.0. **HRMS (ESI)** calcd. for $C_{28}H_{21}N$ (M + H^+) 372.1747. Found 372.1742. **HPLC** (IA column, Isopropanol/n-hexane = 10/90, flow rate = 1.0 mL/min, T= 30°C, l = 254 nm) t_R = 6.21 min (major), 9.40 min (minor).

(*R*)-1-(2-(1-(2-fluorophenyl)vinyl) naphthalen-1-yl) isoquinoline (3Ae).

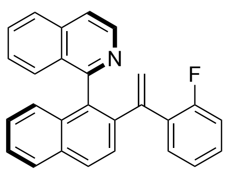

Following the general procedure, purification by column chromatography (*n*-Hexane/EtOAc, 9:1 to 4:1) afforded **3Ae** (31 mg, 82%) as a white amorphous solid**.** $[\alpha]^{20}_D$ +194.5 (*c* 0.4, $CHCl_3$) for 89 % *ee.* **^{1}H NMR (400 MHz, $CDCl_3$):** *δ* 8.48 (d, *J* = 5.7 Hz, 1H), 8.06 (d, *J* = 8.5 Hz, 1H), 7.97 (d, *J* = 8.2 Hz, 1H), 7.75 (dd, *J* = 8.4, 4.0 Hz, 2H), 7.61 – 7.50 (m, 2H), 7.47 (t, *J* = 7.5 Hz, 1H), 7.39 (d, *J* = 8.4 Hz, 1H), 7.27 (dd, *J* = 13.1, 5.6 Hz, 2H), 7.09 (d, *J* = 8.6 Hz, 1H), 6.82 – 6.72 (m, 1H), 6.60 (p, *J* = 7.5 Hz, 2H), 6.53 – 6.45 (m, 1H), 5.58 (s, 1H), 5.40 (s, 1H). **^{13}C NMR (101 MHz, $CDCl_3$)**: δ 159.4, 159.3 (d, *J* = 249 Hz), 144.7, 141.7, 139.7, 136.0, 133.1, 132.6, 130.2, 129.1, 128.6, 128.3 (d, *J* = 8 Hz), 128.1, 128.0, 127.0, 126.6 (d, *J* = 9 Hz), 126.4, 126.0, 123.3 (d, *J* = 3 Hz), 121.6, 120.3, 114.9 (d, *J* = 22 Hz). **^{19}F NMR (377 MHz, $CDCl_3$)**: δ –113.1. **HRMS (ESI**) calcd. for $C_{27}H_{18}NF$ (M + H^+) 376.1496. Found 376.1492. **HPLC** (IA column, Isopropanol/n-hexane = 10/90, flow rate = 1.0 mL/min, T= 30°C, l = 254 nm) t_R = 6.29 min (major), 10.98 min (minor).

(*R*)-1-(2-(1-(2,4-difluorophenyl)vinyl) naphthalen-1-yl) isoquinoline (3Af).

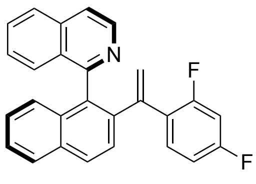

Following the general procedure, purification by column chromatography (*n*-Hexane/EtOAc, 9:1 to 4:1) afforded **3Af** (32 mg, 82%) as a white amorphous solid**.** $[\alpha]^{20}_D$ +88.7 (*c* 0.4, $CHCl_3$) for 93 % *ee.* **^{1}H NMR (400 MHz, $CDCl_3$)**: *δ* 8.50 (d, *J* = 5.7 Hz, 1H), 8.06 (d, *J* = 8.5 Hz, 1H), 7.97 (d, *J* = 8.2 Hz, 1H), 7.76 (dd, *J* = 13.1, 8.4 Hz, 2H), 7.66 – 7.54 (m, 2H), 7.48 (t, *J* = 7.5 Hz, 1H), 7.37 (d, *J* = 8.4 Hz, 1H), 7.33 – 7.23 (m, 2H), 7.10 (d, *J* = 8.5 Hz, 1H), 6.55 (dd, *J* = 15.3, 8.6 Hz, 1H), 6.30 (td, *J* = 8.4, 2.4 Hz, 1H), 6.26 – 6.17 (m, 1H), 5.58 (s, 1H),

5.37 (s, 1H). **^{13}C NMR (100 MHz, CDCl$_3$)**: *δ* 161.4 (dd, *J* = 235, 12 Hz), 159.3, 159.2 (dd, *J* = 238, 12 Hz), 143.9, 141.6, 139.5, 136.0, 133.7, 133.2, 132.6, 130.9, 130.8 (dd, *J* = 9, 5 Hz), 129.2, 128.5, 128.1, 128.0, 127.9, 127.2, 126.8, 126.7, 126.4, 126.1, 125.4 (dd, *J* = 13, 4 Hz), 121.7, 120.4, 110.4 (dd, *J* = 21, 3 Hz), 103.1 (t, *J* = 26 Hz). **^{19}F NMR (377 MHz, CDCl$_3$)**: δ –108.9 (d, *J* = 7.9 Hz), –112.5 (d, *J* = 7.9 Hz). **HRMS (ESI)** calcd. for $C_{27}H_{17}NF_2$ (M + H$^+$) 394.1402. Found 394.1399. **HPLC** (IA column, Isopropanol/n-hexane = 10/90, flow rate = 1.0 mL/min, T= 30°C, l = 254 nm) t_R = 6.18 min (major), 10.09 min (minor).

(*R*)-1-(2-(1-(3-methoxyphenyl)vinyl) naphthalen-1-yl) isoquinoline (3Ag).

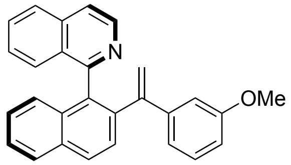

Following the general procedure, purification by column chromatography (*n*-Hexane/EtOAc, 4:1 to 7:3) afforded **3Ag** (37 mg, 95%) as a viscous oil. $[\alpha]^{20}_D$ +28.8 (*c* 0.5, CHCl$_3$) for 93 % *ee*. **^{1}H NMR (400 MHz, CDCl$_3$):** *δ* 8.53 (d, *J* = 5.7 Hz, 1H), 8.03 (d, *J* = 8.5 Hz, 1H), 7.97 (d, *J* = 8.2 Hz, 1H), 7.79 (d, *J* = 8.2 Hz, 1H), 7.64 (d, *J* = 8.5 Hz, 1H), 7.61 – 7.55 (m, 2H), 7.49 (t, *J* = 7.1 Hz, 1H), 7.43 (d, *J* = 8.4 Hz, 1H), 7.33 – 7.26 (m, 2H), 7.16 (d, *J* = 8.5 Hz, 1H), 6.87 (t, *J* = 7.9 Hz, 1H), 6.56 (d, *J* = 7.7 Hz, 1H), 6.50 (dd, *J* = 8.2, 2.5 Hz, 1H), 6.46 – 6.41 (m, 1H), 5.34 (d, *J* = 1.0 Hz, 1H), 5.26 (s, 1H), 3.63 (s, 3H). **^{13}C NMR (100 MHz, CDCl$_3$)**: *δ* 159.7, 158.7, 149.4, 142.7, 142.1, 139.6, 135.8, 135.1, 133.0, 132.7, 129.8, 129.3, 128.6, 128.5, 128.2, 127.9, 127.8, 126.6, 126.5, 125.9, 120.1, 119.9, 117.6, 112.7, 112.6, 55.0. **HRMS (ESI)** calcd. for $C_{28}H_{21}ON$ (M + H$^+$) 388.1696. Found 388.1693. **HPLC** (IA column, Isopropanol/n-hexane = 10/90, flow rate = 1.0 mL/min, T= 30°C, l = 254 nm) t_R = 7.14 min (major), 10.49 min (minor).

(*R*)-3-methyl-2-(2-(1-phenylvinyl) naphthalen-1-yl) pyridine (3Ba).

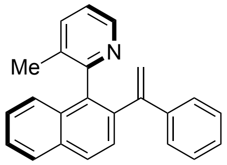

Following the general procedure, purification by column chromatography (*n*-Hexane/EtOAc, 9:1 to 4:1) afforded **3Ba** (27 mg, 84%) as a colorless viscous oil. $[\alpha]^{20}_D$ +50.1 (*c* 0.4, CHCl$_3$) for 95 % *ee.* **^{1}H NMR (400 MHz, CDCl$_3$)**: *δ* 8.40 (d, *J* = 4.3 Hz, 1H), 7.99 – 7.87 (m, 2H), 7.55 (d, *J* = 8.4 Hz, 1H), 7.49 (t, *J* = 7.5 Hz, 1H), 7.39 (dd, *J* = 17.1, 8.2 Hz, 2H), 7.22 (d, *J* = 8.4 Hz, 1H), 7.18 – 7.06 (m, 6H), 5.51 (s, 1H), 5.28 (s, 1H), 1.90 (s, 3H). **^{13}C NMR (100 MHz, CDCl$_3$):** *δ* 157.3, 149.4, 146.6, 141.6, 138.5, 137.2, 136.2, 133.12, 133.08, 131.9, 128.4, 128.04, 127.97, 127.7, 127.1, 126.5, 125.9, 125.8, 122.1, 117.2, 19.0. **HRMS (ESI)** calcd. for $C_{24}H_{19}ON$ (M + H$^+$) 322.1590. Found 322.1591. **HPLC** (IA column, Isopropanol/n-hexane = 10/90, flow rate = 1.0 mL/min, T= 30°C, l = 254 nm) t_R = 5.16 min (major), 6.68 min (minor).

(*R*)-2-(2-(1-(2-fluorophenyl)vinyl)naphthalen-1-yl)-3-methylpyridine (3Be).

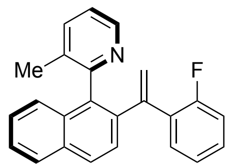

Following the general procedure, purification by column chromatography (*n*-Hexane/EtOAc, 9:1 to 4:1) afforded **3Be** (27 mg, 79%) as a viscous oil. $[\alpha]^{20}_{D}$ +62.7 (*c* 0.5, $CHCl_3$) for 93% *ee.* **^{1}H NMR (400 MHz, $CDCl_3$):** δ 8.33 (d, *J* = 4.6 Hz, 1H), 7.95 (dd, *J* = 16.4, 8.3 Hz, 2H), 7.67 (d, *J* = 8.5 Hz, 1H), 7.47 (t, *J* = 7.5 Hz, 1H), 7.36 (t, *J* = 7.7 Hz, 2H), 7.18 (d, *J* = 8.4 Hz, 1H), 7.12–7.01 (m, 2H), 6.89 – 6.74 (m, 3H), 5.59 (s, 1H), 5.54 (s, 1H), 1.86 (s, 3H). **^{13}C NMR (100 MHz, $CDCl_3$)**: δ 159.5 (d, *J* = 249 Hz), 156.9, 146.4, 144.7, 138.7, 137.5, 135.2, 133.6, 133.2, 131.7, 130.4 (d, *J* = 3 Hz), 129.7 (d, *J* = 13 Hz), 128.6, 128.5, 128.2, 126.7, 125.8 (d, *J* = 14 Hz), 123.6 (d, *J* = 3 Hz), 122.3, 121.5, 115.2 (d, *J* = 22 Hz), 18.8. **^{19}F NMR (377 MHz, $CDCl_3$)**: δ –113.8. **HRMS (ESI)** calcd. for $C_{24}H_{18}NF$ (M + H^+) 340.1496. Found 340.1496. **HPLC** (IA column, Isopropanol/n-hexane = 10/90, flow rate = 1.0 mL/min, T= 30°C, l = 254 nm) t_R = 10.24 min (major), 12.31 min (minor).

(*R*)-2-(2-(1-(3-methoxyphenyl)vinyl)naphthalen-1-yl)-3-methylpyridine (3Bg).

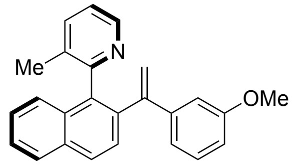

Following the general procedure, purification by column chromatography (*n*-Hexane/EtOAc, 9:1 to 4:1) afforded **3Bg** (25 mg, 72%) as a light yellow viscous oil. $[\alpha]^{20}_{D}$ +30.3 (*c* 0.3, $CHCl_3$) for 92 % *ee.* **^{1}H NMR (400 MHz, $CDCl_3$):** δ 8.40 (d, *J* = 4.7 Hz, 1H), 7.90 (d, *J* = 8.3 Hz, 2H), 7.55 (d, *J* = 8.5 Hz, 1H), 7.46 (t, *J* = 7.4 Hz, 1H), 7.38 – 7.33 (m, 2H), 7.19 (d, *J* = 8.5 Hz, 1H), 7.08 (m, 1H), 7.03 (d, *J* = 8.0 Hz, 1H), 6.70–6.66 (m, 2H), 6.60 (s, 1H), 5.46 (d, *J* = 1.4 Hz, 1H), 5.23 (d, *J* = 1.4 Hz, 1H), 3.70 (s, 3H), 1.86 (s, 3H). **^{13}C NMR (100 MHz, $CDCl_3$):** δ 159.0, 157.3, 149.2, 146.4, 143.2, 138.5, 137.2, 136.2, 133.2, 133.0, 131.9, 129.3, 128.7, 128.3, 128.1, 128.0, 126.5, 125.84, 125.79, 122.1, 120.0, 117.6, 112.8, 112.6, 55.2, 19.1. **HRMS (ESI)** calcd. for $C_{25}H_{21}ON$ (M + H^+) 352.1696. Found 352.1696. **HPLC** (IA column, Isopropanol/n-hexane = 10/90, flow rate = 1.0 mL/min, T= 30°C, l = 254 nm) t_R = 5.90 min (major), 7.52 min (minor).

(*R*)-2-(2-(1-(6-methoxynaphthalen-2-yl)vinyl)naphthalen-1-yl)-3-ethylpyridine (3Bh).

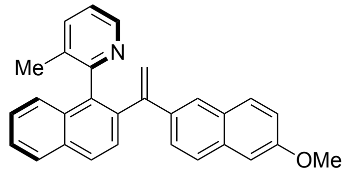

Following the general procedure, purification by column chromatography (*n*-Hexane/EtOAc, 4:1 to 3:2) afforded **3Bh** (29 mg, 72%) as a viscous oil. $[\alpha]^{20}_{D}$ +90.4 (*c* 0.2, $CHCl_3$) for 93 % *ee.* **^{1}H NMR (400 MHz, $CDCl_3$):** δ 8.31 (dd, *J* = 4.7, 1.6 Hz, 1H), 7.93 (d, *J* = 8.5 Hz, 1H), 7.92 (d, *J* = 8.2 Hz, 1H), 7.56 (d, *J* = 8.5 Hz, 1H), 7.55 – 7.45 (m, 3H), 7.42 (s, 1H), 7.36 (d, *J* = 7.3 Hz, 1H), 7.29 – 7.24 (m, 2H), 7.20 (d, *J* = 8.5 Hz, 1H), 7.07 – 7.04 (m, 2H), 6.98 (dd, *J* = 7.8, 4.8 Hz, 1H) 5.57 (d, *J* = 1.4 Hz, 1H), 5.25 (d, *J* = 1.4 Hz, 1H), 3.90 (s, 3H), 1.86 (s, 3H). **^{13}C NMR (100 MHz, $CDCl_3$):** δ 157.6, 157.4, 149.1, 146.4, 138.6, 137.1,

136.8, 136.4, 133.8, 133.1, 132.0, 129.7, 128.4 (2 x C), 128.1, 127.9, 126.5, 126.14, 126.09, 125.92, 125.85, 125.8, 122.0, 118.6, 117.0, 105.5, 55.3, 19.1. **HRMS (ESI)** calcd. for $C_{29}H_{23}ON$ (M + H^+) 402.1852. Found 402.1851. **HPLC** (IA column, Isopropanol/n-hexane = 10/90, flow rate = 1.0 mL/min, T= 30°C, l = 254 nm) t_R = 7.01 min (major), 8.93 min (minor).

(*R*)-1-(4-methoxy-2-(1-phenylvinyl)naphthalen-1-yl)isoquinoline (3Ca).

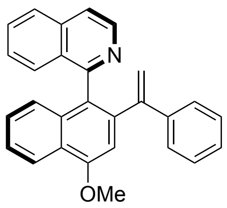

Following the general procedure, purification by column chromatography (*n*-Hexane/EtOAc, 4:1 to 7:3) afforded **3Ca** (31 mg, 81%) as a light yellow viscous oil. $[\alpha]^{20}_D$ +147.3 (*c* 0.6, $CHCl_3$) for 95% *ee.* **^{1}H NMR (400 MHz, $CDCl_3$)**: δ 8.48 (d, *J* = 5.6 Hz, 1H), 8.39 (d, *J* = 8.6 Hz, 1H), 7.78 (d, *J* = 8.2 Hz, 2H), 7.63–7.53 (m, 2H), 7.52–7.45 (m, 2H), 7.32-7.27 (m, 2H), 7.10 (d, *J* = 8.4 Hz, 1H), 7.01–6.93 (m, 5H), 5.35 (s, 1H), 5.25 (s, 1H), 4.10 (s, 3H).**^{13}C NMR (100 MHz, CDCl3):** δ 159.5, 158.7, 155.7, 149.5, 142.4, 140.2, 136.0, 133.7, 130.2, 128.9, 128.5, 128.2, 127.1, 126.8, 126.5, 126.1, 125.3, 125.0, 122.0, 120.1, 117.7, 112.7, 112.5, 106.4, 55.8, 55.0. **HRMS (ESI)** calcd. for $C_{28}H_{21}ON$ (M + H^+) 388.1696. Found 388.1693. **HPLC** (IA column, Isopropanol/n-hexane = 10/90, flow rate = 1.0 mL/min, T= 30°C, l = 254 nm) t_R = 7.68 min (major), 9.27 min (minor).

(*R*)-1-(4-methoxy-2-(1-(3-methoxyphenyl)vinyl)naphthalen-1-yl)isoquinoline (3Cg).

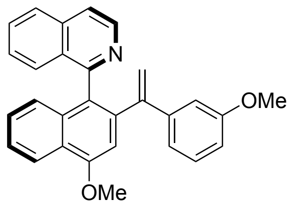

Following the general procedure, purification by column chromatography (*n*-Hexane/EtOAc, 4:1 to 3:2) afforded **3Cg** (35 mg, 85%) as a light yellow viscous oil. $[\alpha]^{20}_D$ +175.0 (*c* 0.4, $CHCl_3$) for 95% *ee.* **^{1}H NMR (400 MHz, $CDCl_3$)**: δ 8.47 (d, *J* = 5.7 Hz, 1H), 8.36 (d, *J* = 8.0 Hz, 1H), 7.73 (d, *J* = 8.2 Hz, 1H), 7.55–7.51 (m, 2H), 7.46–7.42 (m, 2H), 7.29–7.22 (m, 2H), 7.08 (d, *J* = 8.5 Hz, 1H), 6.93 (s, 1H), 6.85 (t, *J* = 7.9 Hz, 1H), 6.56 (d, *J* = 7.7 Hz, 1H), 6.46 (d, *J* = 8.2 Hz, 1H), 6.43 (s, 1H), 5.30 (s, 1H), 5.22 (s, 1H), 4.08 (s, 3H), 3.61 (s, 3H). **^{13}C NMR (100 MHz, $CDCl_3$)**: δ 159.9, 158.7, 155.4, 149.8, 142.5, 142.1, 135.8, 133.7, 129.6, 128.9, 128.4, 128.0, 127.7, 127.0, 126.4 (2 x C), 126.2, 125.2, 125.0, 121.9, 120.1, 119.6, 117.3, 112.7, 112.5, 106.3, 55.7, 55.0. **HRMS (ESI)** calcd. for $C_{29}H_{23}O_2N$ (M + H^+) 418.1802. Found 418.1803. **HPLC** (IA column, Isopropanol/n-hexane = 5/95, flow rate = 1.0 mL/min, T= 30°C, l = 254 nm) t_R = 17.53 min (minor), 18.98 min (major).

(*R*)-1-(4-methoxy-2-(1-(6-methoxynaphthalen-2-yl)vinyl)naphthalen-1-yl)isoquinoline (3Ch).

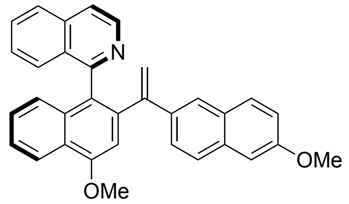

Following the general procedure, purification by column chromatography (*n*-Hexane/EtOAc, 4:1 to 3:2) afforded **3Ch** (47 mg, 84%) as a light yellow viscous oil. $[\alpha]^{20}_{D}$ +152.0 (*c* 0.4, $CHCl_3$) for 94% *ee.* **^{1}H NMR (400 MHz, $CDCl_3$)**: δ 8.46 – 8.38 (m, 2H), 7.66 (d, *J* = 8.3 Hz, 1H), 7.57 – 7.43 (m, 5H), 7.39 (d, *J* = 8.6 Hz, 1H), 7.36 – 7.30 (m, 2H), 7.26 – 7.13 (m, 3H), 7.06 (dd, *J* = 8.9, 2.5 Hz, 1H), 7.00 – 6.96 (m, 2H), 5.43 (s, 1H), 5.27 (s, 1H), 4.09 (s, 3H), 3.91 (s, 3H). **^{13}C NMR (100 MHz, $CDCl_3$)**: δ 159.8, 157.6, 155.5, 149.5, 141.8, 140.1, 136.2, 135.8, 133.8, 133.6, 129.73, 129.67, 129.3, 129.0, 128.3, 127.9, 127.1, 126.6, 126.42, 126.39, 126.3, 126.0, 125.9, 125.3, 125.1, 122.0, 119.7, 118.5, 116.9, 106.4, 105.4, 55.8, 55.3. **HRMS (ESI)** calcd. for $C_{33}H_{25}O_2N$ (M + H$^+$) 468.1958. Found 468.1954. **HPLC** (IA column, Isopropanol/n-hexane = 2/98, flow rate = 1.0 mL/min, T= 30°C, l = 254 nm) t_R = 47.04 min (major), 56.56 min (minor).

(*R*)-4-(2-(1-phenylvinyl)naphthalen-1-yl)quinazoline (3Da).

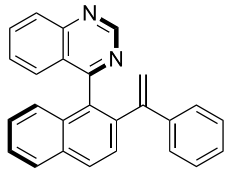

Following the general procedure, purification by column chromatography (*n*-Hexane/EtOAc, 4:1 to 7:3) afforded **3Da** (29 mg, 81%) as a colorless viscous oil. $[\alpha]^{20}_{D}$ +100.4 (*c* 0.75, $CHCl_3$) for 91% *ee.* **^{1}H NMR (400 MHz, $CDCl_3$)**: δ 9.26 (s, 1H), 8.08 (d, *J* = 8.5 Hz, 1H), 8.02 (dd, *J* = 16.1, 8.4 Hz, 2H), 7.83 (t, *J* = 7.6 Hz, 1H), 7.66 (d, *J* = 8.5 Hz, 1H), 7.53 (t, *J* = 7.5 Hz, 1H), 7.46 (d, *J* = 8.3 Hz, 1H), 7.36 (dd, *J* = 16.2, 8.2 Hz, 2H), 7.17 (d, *J* = 8.5 Hz, 1H), 6.96 (dd, *J* = 30.0, 5.8 Hz, 5H), 5.39 (s, 1H), 5.25 (s, 1H). **^{13}C NMR (100 MHz, $CDCl_3$)**: δ 169.0, 154.3, 149.8, 149.0, 140.7, 139.6, 133.8, 132.9, 132.4, 131.9, 129.4, 128.3, 128.23, 128.18, 127.7, 127.5, 127.4, 127.3, 127.2, 127.0, 126.2, 125.9, 125.3, 117.9. **HRMS (ESI)** calcd. for $C_{26}H_{18}N_2$ (M + H$^+$) 359.1542. Found 359.1543. **HPLC** (IA column, Isopropanol/n-hexane = 10/90, flow rate = 1.0 mL/min, T= 30°C, l = 254 nm) t_R = 6.90 min (major), 9.28 min (minor).

(*R*)-4-(2-(1-(4-chlorophenyl)vinyl) naphthalen-1-yl)quinazoline (3Dc).

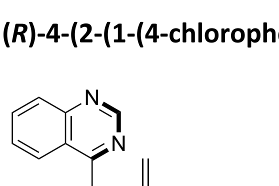

Following the general procedure, purification by column chromatography (*n*-Hexane/EtOAc, 4:1 to 7:3) afforded **3Dc** (36 mg, 92%) as a colorless viscous oil. $[\alpha]^{20}_{D}$ +90.1 (*c* 0.7, $CHCl_3$) for 87% *ee.* **^{1}H NMR (400 MHz, $CDCl_3$)**: δ 9.28 (s, 1H), 8.07 (dd, *J* = 12.6, 8.5 Hz, 2H), 8.00 (d, *J* = 8.2 Hz, 1H), 7.85 (t, *J* = 7.3 Hz, 1H), 7.63 (d, *J* = 8.5 Hz, 1H), 7.53 (t, *J* = 7.5 Hz, 1H), 7.44 – 7.32 (m, 3H), 7.17 (d, *J* = 8.5 Hz, 1H), 6.94 (d, *J* = 8.1 Hz, 2H), 6.85 (d, *J* = 8.2 Hz, 2H), 5.37 (s, 1H), 5.25 (s, 1H). **^{13}C NMR (100 MHz, $CDCl_3$)**: δ 168.8, 154.3, 149.8,

147.9, 139.2, 139.1, 133.9, 133.2, 132.9, 132.5, 131.8, 129.6, 128.5, 128.2, 128.0, 127.9, 127.5, 127.4, 127.2, 126.4, 125.8, 125.3, 118.2. **HRMS (ESI)** calcd. for $C_{26}H_{17}N_2Cl$ (M + H^+) 393.1153. Found 393.1150. **HPLC** (IA column, Isopropanol/n-hexane = 10/90, flow rate = 1.0 mL/min, T= 30°C, l = 254 nm) t_R = 7.61 min (major), 9.65 min (minor).

(*R*)-4-(2-(1-(p-tolyl)vinyl)naphthalen-1-yl)quinazoline (3Dd).

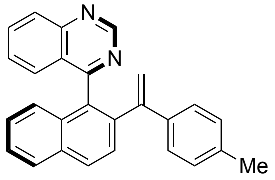

Following the general procedure, purification by column chromatography (*n*-Hexane/EtOAc, 4:1 to 7:3) afforded **3Dd** (31 mg, 82%) as a viscous oil. $[\alpha]^{20}_D$ +122.5 (*c* 0.4, $CHCl_3$) for 93% *ee.* **^{1}H NMR (400 MHz, $CDCl_3$)**: *δ* 9.27 (s, 1H), 8.05 (dd, *J* = 12.4, 8.5 Hz, 2H), 7.99 (d, *J* = 8.2 Hz, 1H), 7.83 (t, *J* = 7.6 Hz, 1H), 7.65 (d, *J* = 8.5 Hz, 1H), 7.52 (t, *J* = 7.5 Hz, 1H), 7.46 (d, *J* = 8.1 Hz, 1H), 7.40 – 7.31 (m, 2H), 7.16 (d, *J* = 8.5 Hz, 1H), 6.81 (q, *J* = 8.2 Hz, 4H), 5.35 (s, 1H), 5.18 (s, 1H), 2.18 (s, 3H). **^{13}C NMR (100 MHz, $CDCl_3$)**: *δ* 169.1, 154.4, 149.8, 148.8, 139.9, 137.9, 137.1, 133.6, 132.8, 132.4, 131.9, 129.3, 128.42, 128.37, 128.2, 128.1, 127.6, 127.3, 127.2, 127.0, 126.2, 125.9, 125.4, 117.1, 20.9. **HRMS (ESI)** calcd. for $C_{27}H_{21}N_2$ (M + H^+) 373.1699. Found 373.1694. **HPLC** (IA column, Isopropanol/n-hexane = 10/90, flow rate = 1.0 mL/min, T= 30°C, l = 254 nm) t_R = 7.02 min (major), 9.32 min (minor).

(*R*)-4-(2-(1-(2-fluorophenyl)vinyl) naphthalen-1-yl)quinazoline (3De).

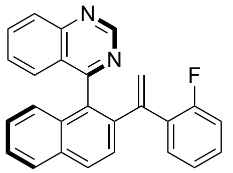

Following the general procedure, purification by column chromatography (*n*-Hexane/EtOAc, 4:1 to 7:3) afforded **3De** (31 mg, 81%) as a light yellow viscous oil. $[\alpha]^{20}_D$ +91.8 (*c* 0.4, $CHCl_3$) for 91% *ee.* **^{1}H NMR (400 MHz, $CDCl_3$):** *δ* 9.25 (s, 1H), 8.10 (d, *J* = 8.5 Hz, 1H), 7.99 (d, *J* = 8.3 Hz, 2H), 7.83 – 7.74 (m, 2H), 7.50 (t, *J* = 7.5 Hz, 1H), 7.42 – 7.30 (m, 3H), 7.09 (d, *J* = 8.5 Hz, 1H), 6.87 – 6.79 (m, 1H), 6.67 – 6.58 (m, 2H), 6.50 (dd, *J* = 10.2, 8.6 Hz, 1H), 5.62 (s, 1H), 5.46 (s, 1H). **^{13}C NMR (100 MHz, $CDCl_3$)**: δ 168.9, 159.3 (d, *J* = 249 Hz), 154.5, 149.9, 144.4, 139.5, 133.8, 133.0, 131.8, 131.7, 130.2 (d, *J* = 3 Hz), 129.8, 128.9 (d, *J* = 8 Hz), 128.7, 128.4, 128.2, 128.0, 127.5 (d, *J* = 5 Hz), 127.1, 126.3, 125.9, 125.3, 123.6 (d, *J* = 3 Hz), 122.0, 115.1 (d, *J* = 22 Hz). **^{19}F NMR (377 MHz, $CDCl_3$)**: δ –112.8. **HRMS (ESI**) calcd. for $C_{26}H_{17}N_2F$ (M + H^+) 377.1449. Found 377.1447. **HPLC** (IA column, Isopropanol/n-hexane = 10/90, flow rate = 1.0 mL/min, T= 30°C, l = 254 nm) t_R = 7.13 min (major), 10.00 min (minor).

(*R*)-4-(2-(1-(3-methoxyphenyl)vinyl) naphthalen-1-yl)quinazoline (3Dg).

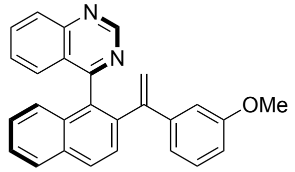

Following the general procedure, purification by column chromatography (*n*-Hexane/EtOAc, 4:1 to 7:3) afforded **3Dg** (30 mg, 76%) as a light yellow viscous oil. $[\alpha]^{20}_{D}$ +49.7 (*c* 0.8, $CHCl_3$) for 89% *ee.* **^{1}H NMR (400 MHz, $CDCl_3$):** δ 9.30 (s, 1H), 8.06 (dd, *J* = 13.6, 8.5 Hz, 2H), 7.99 (d, *J* = 8.2 Hz, 1H), 7.82 (t, *J* = 7.6 Hz, 1H), 7.67 (d, *J* = 8.5 Hz, 1H), 7.52 (t, *J* = 7.5 Hz, 1H), 7.43 (d, *J* = 8.3 Hz, 1H), 7.35 (dd, *J* = 15.3, 7.2 Hz, 2H), 7.16 (d, *J* = 8.5 Hz, 1H), 6.89 (t, *J* = 7.9 Hz, 1H), 6.52 (d, *J* = 7.8 Hz, 2H), 6.41 (s, 1H), 5.39 (s, 1H), 5.28 (s, 1H), 3.63 (s, 3H). **^{13}C NMR (100 MHz, $CDCl_3$)**: δ 169.1, 158.8, 154.2, 149.6, 148.9, 142.1, 139.7, 133.8, 132.9, 132.4, 131.9, 129.4, 128.7, 128.24, 128.20, 128.18, 127.5, 127.3, 127.0, 126.2, 125.9, 125.3, 120.1, 118.1, 112.9, 112.8, 55.1. **HRMS (ESI)** calcd. for $C_{27}H_{20}ON_2$ (M + H^+) 389.1648. Found 389.1643. **HPLC** (IA column, Isopropanol/n-hexane = 10/90, flow rate = 1.0 mL/min, T= 30°C, l = 254 nm) t_R = 8.40 min (major), 10.82 min (minor).

(*R*)-4-(2-(1-(6-methoxynaphthalen-2-yl)vinyl)naphthalen-1-yl)quinazoline (3Dh).

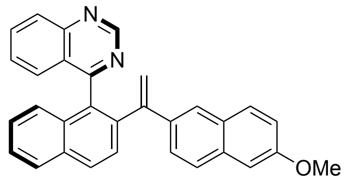

Following the general procedure, purification by column chromatography (*n*-Hexane/EtOAc, 4:1 to 3:2) afforded **3Dh** (35 mg, 79%) as a viscous oil. $[\alpha]^{20}_{D}$ +133.7 (*c* 0.4, $CHCl_3$) for 91% *ee.* **^{1}H NMR (400 MHz, $CDCl_3$)**: δ 9.19 (s, 1H), 8.10 (d, *J* = 8.5 Hz, 1H), 8.02 (d, *J* = 8.2 Hz, 1H), 7.90 (d, *J* = 8.5 Hz, 1H), 7.69 (dd, *J* = 14.7, 7.9 Hz, 2H), 7.54 (t, *J* = 7.5 Hz, 1H), 7.45 (dd, *J* = 8.4, 4.1 Hz, 2H), 7.36 (dd, *J* = 14.9, 7.8 Hz, 2H), 7.26 (t, *J* = 7.6 Hz, 2H), 7.17 (d, *J* = 8.5 Hz, 1H), 7.11 – 7.02 (m, 2H), 6.96 (d, *J* = 2.3 Hz, 1H), 5.48 (s, 1H), 5.28 (s, 1H), 3.90 (s, 3H). **^{13}C NMR (100 MHz, $CDCl_3$)**: δ 168.9, 157.7, 154.4, 149.7, 148.8, 139.9, 135.9, 133.6, 133.5, 132.9, 132.6, 131.9, 129.6, 129.4, 128.3, 128.2, 128.1, 127.3, 127.0, 126.6, 126.3, 126.2, 125.9, 125.7, 125.3, 118.7, 117.6, 105.4, 55.3. **HRMS (ESI)** calcd. for $C_{31}H_{22}ON_2$ (M + H^+) 439.1804. Found 439.1802. **HPLC** (IA column, Hex:Isop 90:10, T= 30°C, F= 1.0 mL/min): t_R 10.87 min (major) and 17.15 min (minor).

5. Product transformations.

(R_a)-**4Aa.**

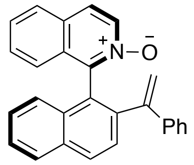

Over a cooled (0 °C) solution of *(R_a)*-**3Aa** (0.16 mmol, 57 mg) in DCM (6 mL), *m*-CPBA (77% purity, 0.22 mmol, 54.2 mg) was added in portions during a 30 min period. After 3h at this temperature the reaction was stopped by dilution with DCM (10 mL) and

addition of 10 mL $NaHCO_3$ (sat. aq.). The organic phase separated, washed with $NaHCO_3$ (sat. aq., 3 x 5 mL), dried over $MgSO_4$, filtered and concentrated. Purification by flash chromatography (EtOAc) afforded *(R_a)*-**4Aa** (38 mg, 64%) as a light yellow foam. $[\alpha]^{20}_{D}$ –16 (*c* 1.0, $CHCl_3$) for 95 % e.e. **^{1}H NMR (400 MHz, $CDCl_3$):** δ 8.10 (d, *J* = 7.2 Hz, 1H), 8.03 (d, *J* = 8.5 Hz, 1H), 7.96 (d, *J* = 8.2 Hz, 1H), 7.72 (d, *J* = 8.1 Hz, 1H), 7.58-7.56 (m, 2H), 7.49 (t, *J* = 8.2 Hz, 1H), 7.44 (t, *J* = 8.1 Hz, 1H), 7.36 (t, *J* = 8.2 Hz, 1H), 7.28 (t, *J* = 8.2 Hz, 1H), 7.15 (d, *J* = 8.1 Hz, 1H), 7.10-7.05 (m, 6H), 5.47 (d, *J* = 0.9 Hz, 1H), 5.35 (d, *J* = 0.9 Hz, 1H) ppm. **^{13}C NMR (100 MHz, $CDCl_3$):** δ 148.3, 145.3, 140.6, 140.5, 136.9, 133.1, 131.7, 129.9, 129.6, 128.7, 128.4 (2xC), 128.1 (2xC), 127.7 (2xC), 127.34, 127.29, 127.1 (2xC), 127.0, 126.5, 126.3, 125.6, 125.4, 123.5, 116.9 ppm. **HRMS (ESI**) calcd. for $C_{27}H_{20}NO$ (M + H^+) 374.1539. Found 374.1538.

(R_a)-**5Aa.**

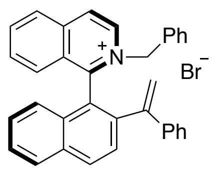

Over a solution of *(R_a)*-**3Aa** (0.1 mmol, 36 mg) in DCM (1 mL), benzyl bromide (0.2 mmol, 24 μL) was added and the resulting solution was stirred for 20 hours. Then, the reaction mixture was concentrated in the rotavapor and the resulting crude was triturated and washed with pentane to give *(R_a)*-**5Aa** (48 mg, 91%) as a light yellow foam. $[\alpha]^{20}_{D}$ +47 (*c* 1.0, $CHCl_3$) for 95 % e.e. **^{1}H NMR (400 MHz, $CDCl_3$):** δ 9.83 (d, *J* = 6.9 Hz, 1H), 8.42 (d, *J* = 6.8 Hz, 1H), 8.34 (d, *J* = 8.4 Hz, 1H), 8.04 (d, *J* = 8.3 Hz, 1H), 7.96 (t, *J* = 8.4 Hz, 1H), 7.93 (d, *J* = 8.4 Hz, 1H), 7.83 (t, *J* = 7.6 Hz, 1H), 7.51 (t, *J* = 8.0 Hz, 1H), 7.43 (t, *J* = 7.7 Hz, 1H), 7.20 (d, *J* = 8.6 Hz, 1H), 7.13 (t, *J* = 8.0 Hz, 1H), 7.00 (t, *J* = 7.6 Hz, 1H), 6.91 (t, *J* = 7.6 Hz, 2H), 6.82-6.78 (m, 4H), 6.68-6.64 (m, 3H), 6.42 (d, *J* = 8.0 Hz, 1H), 6.38 (d, *J* = 14 Hz, 1H), 5.70 (s, 1H), 5.60 (s, 1H), 5.36 (d, *J* = 14 Hz, 1H) ppm. **^{13}C NMR (100 MHz, $CDCl_3$):** δ 157.1, 148.2, 141.8, 138.4, 138.0, 137.1, 135.7, 132.9, 132.6, 132.2, 131.4, 130.4, 130.1, 129.2 (2xC), 129.1, 128.7 (2xC), 128.6, 128.4, 128.3, 127.9 (2xC), 127.6 (2xC), 127.2, 126.9, 126.6 (2xC), 124.8, 123.9, 121.6, 62.4 ppm. **HRMS (ESI)** calcd. for $C_{34}H_{26}N^+$ (M^+) 448.2060. Found 448.2045.

6. NMR spectra and HPLC traces.

^{1}H NMR (400MHz, $CDCl_3$) of **1D.**

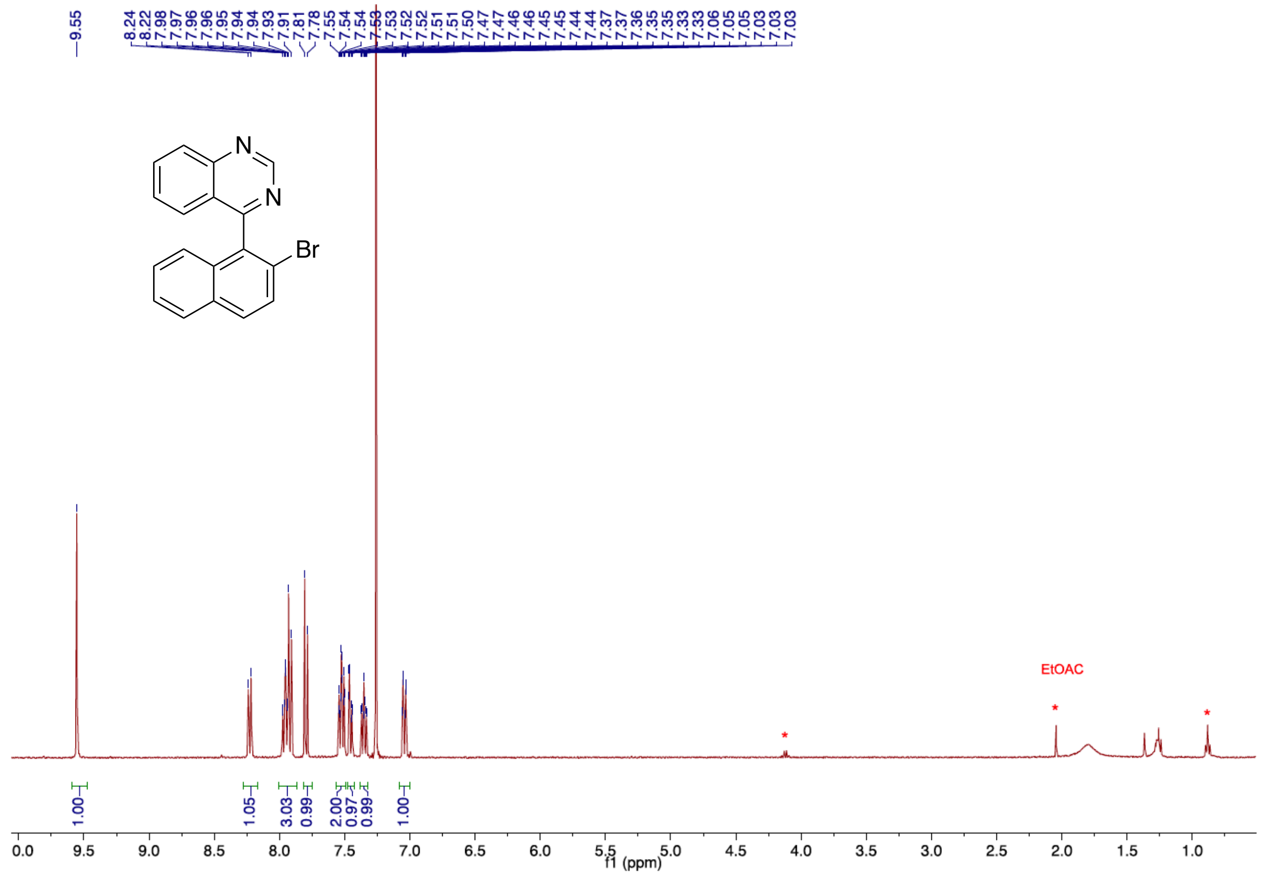

^{13}C NMR (100MHz, $CDCl_3$) of **1D.**

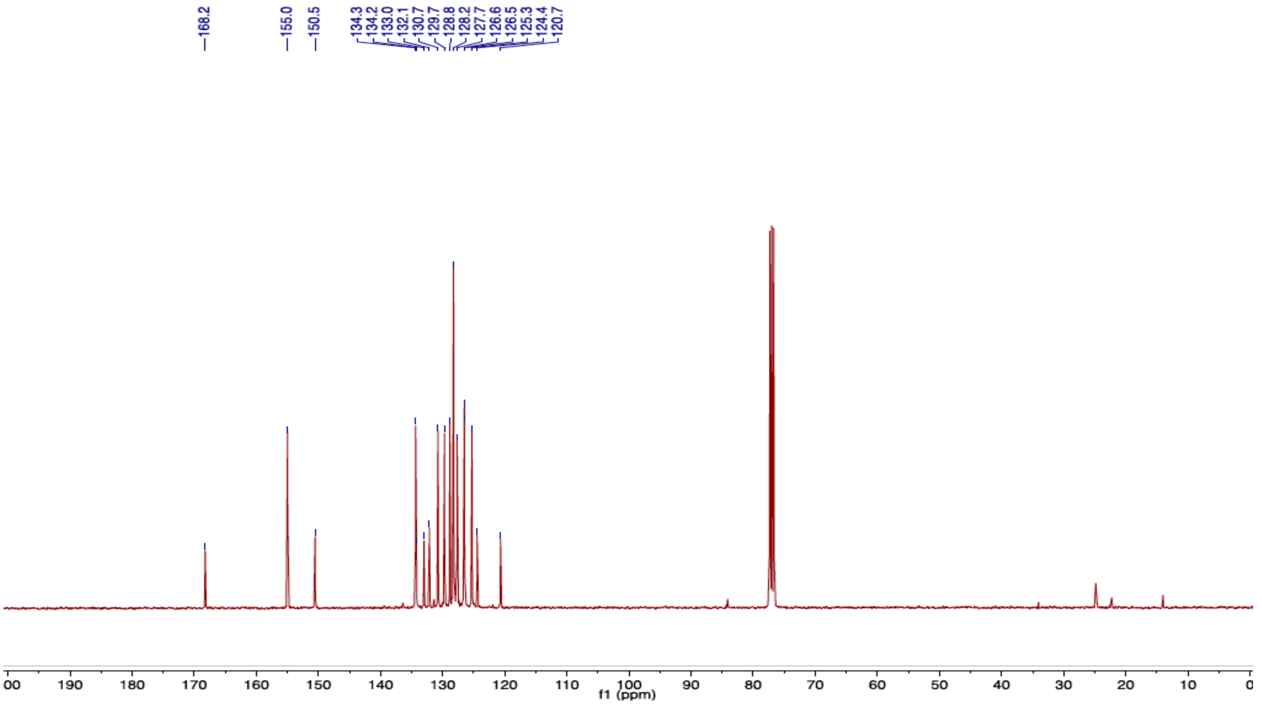

^{1}H NMR (400MHz, $CDCl_3$) of **3Aa.**

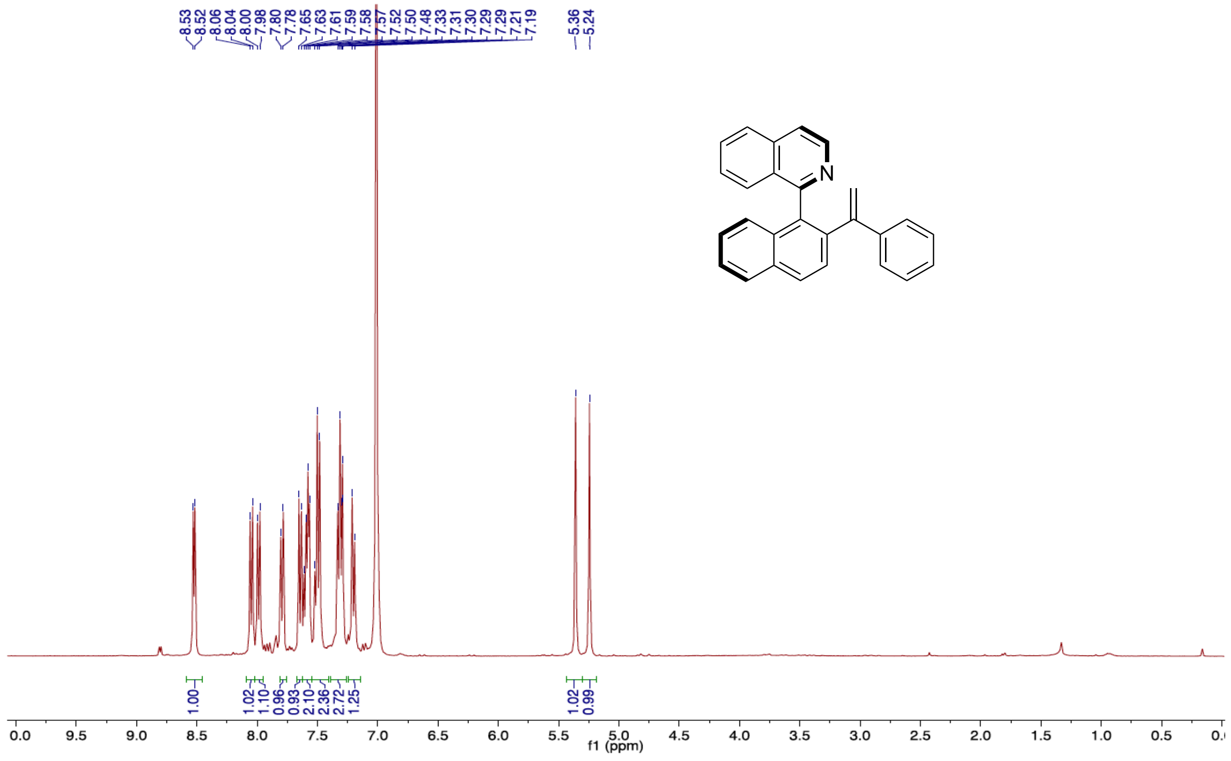

^{13}C NMR (100MHz, $CDCl_3$) of **3Aa.**

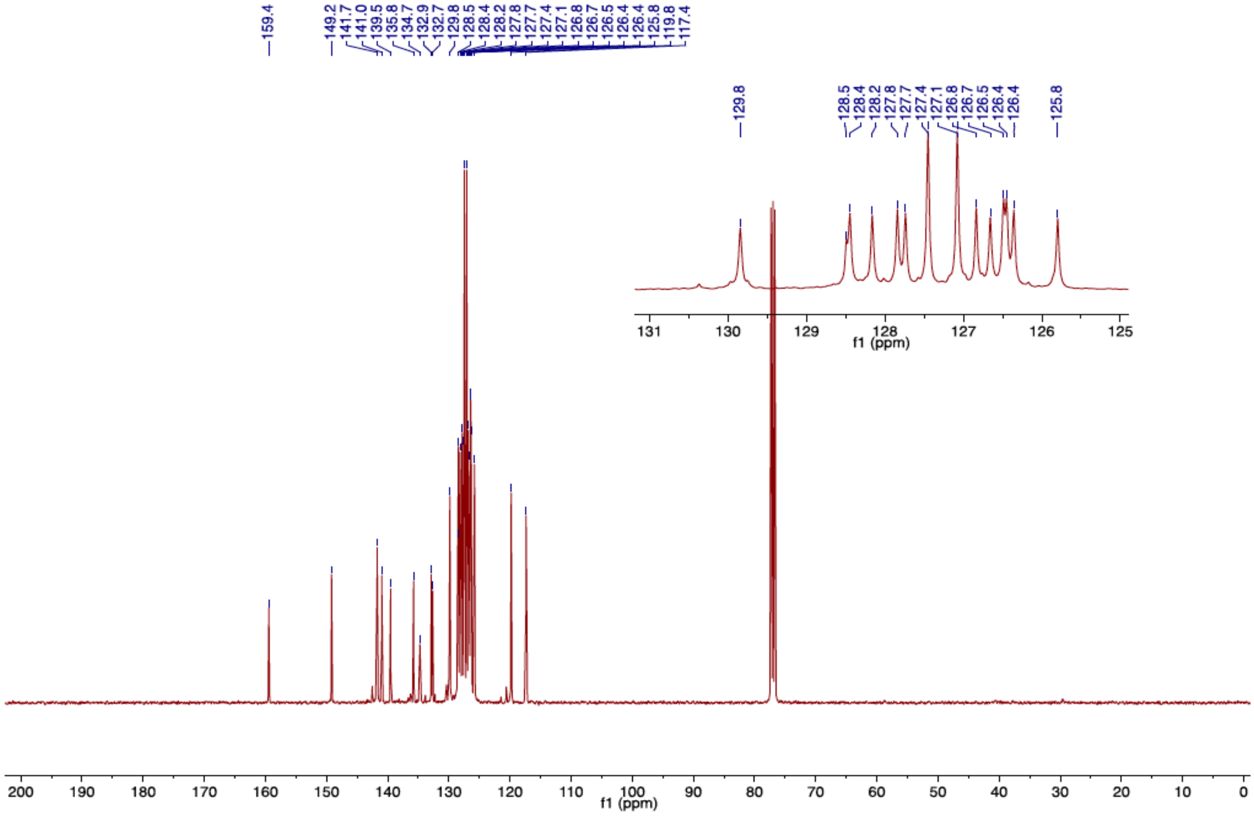

Racemic sample of 3Aa: IA column, Hex:Isop 90:10, T= 30°C, F= 1.0 mL/min.

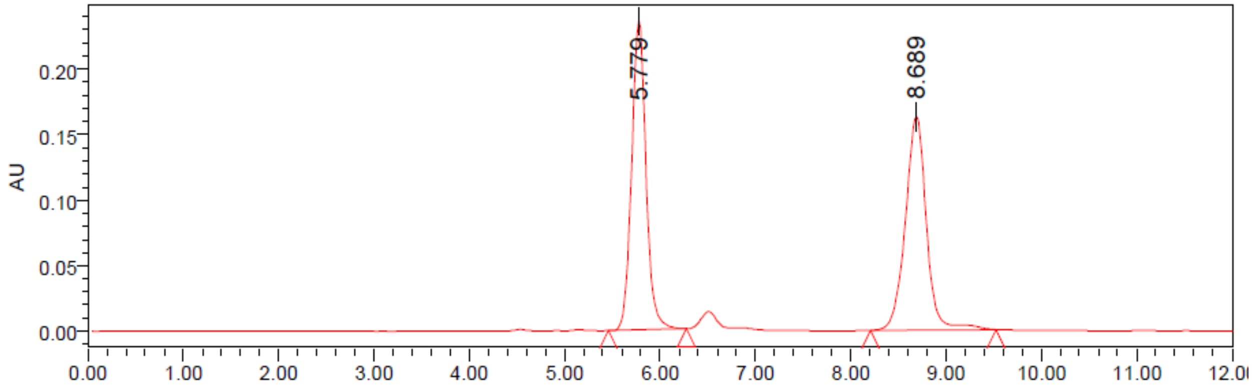

	Processed Channel	Retention Time (min)	Area	% Area	Height
1	PDA 249.8 nm	5.779	2449638	49.51	235797
2	PDA 249.8 nm	8.689	2497803	50.49	162912

Enantioriched sample of 3Aa.

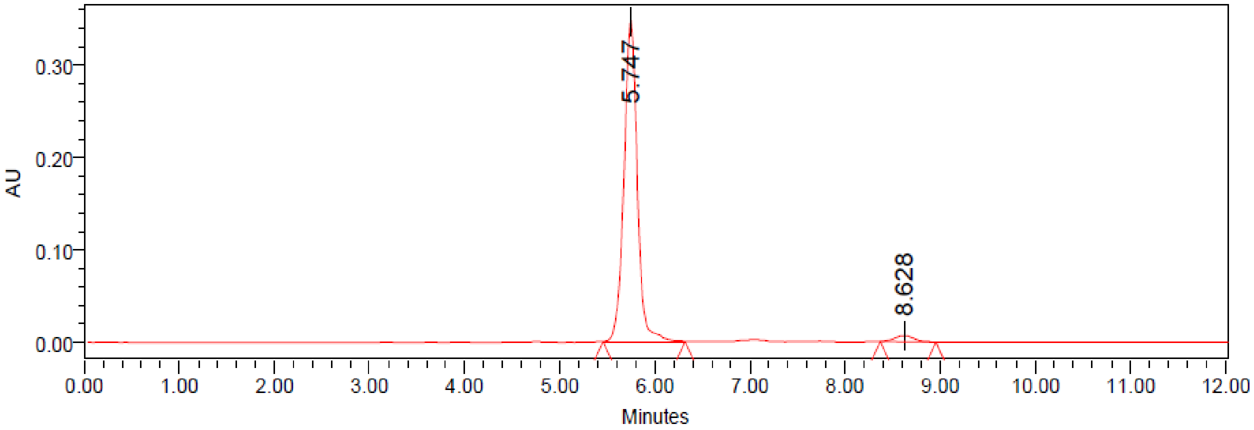

Processed Channel: PDA 252.7 nm

	Processed Channel	Retention Time (min)	Area	% Area	Height
1	PDA 252.7 nm	5.747	3374292	97.40	347898
2	PDA 252.7 nm	8.628	90059	2.60	6722

^{1}H NMR (400MHz, $CDCl_3$) of **3Ab.**

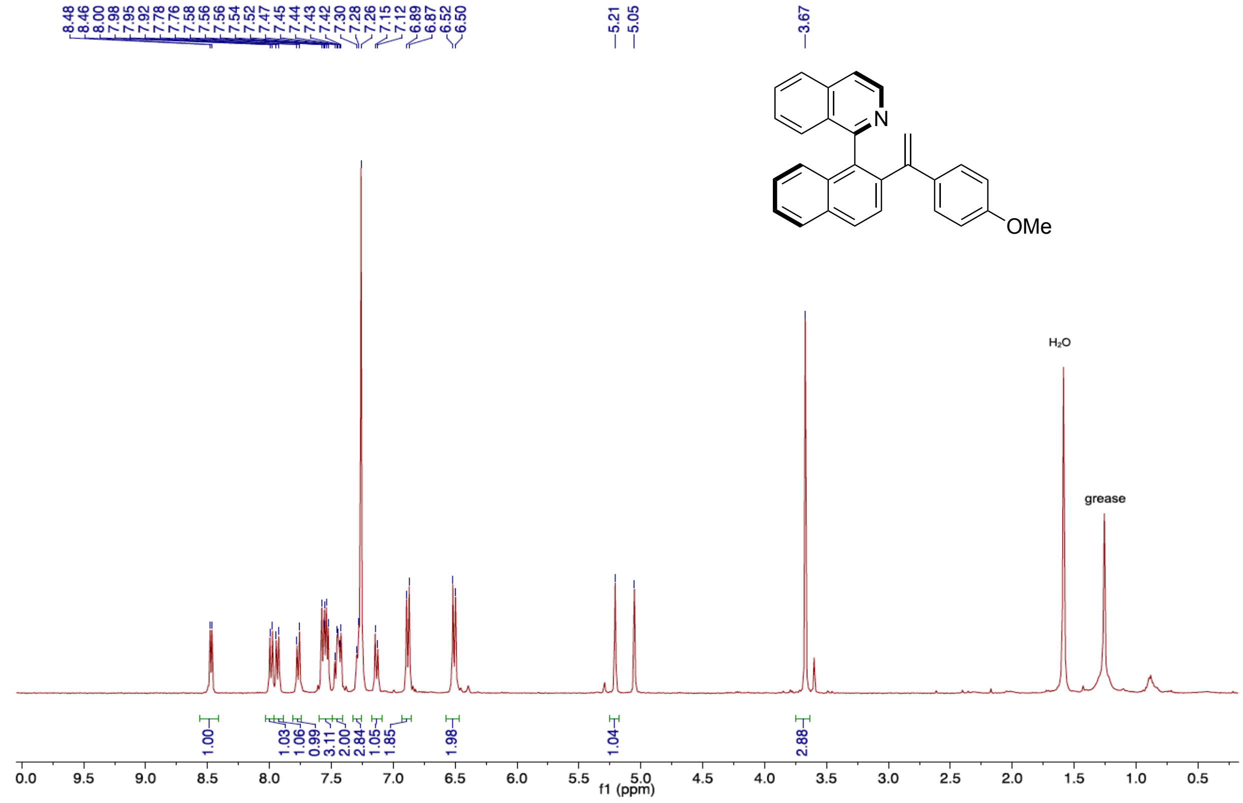

^{13}C NMR (100MHz, $CDCl_3$) of **3Ab.**

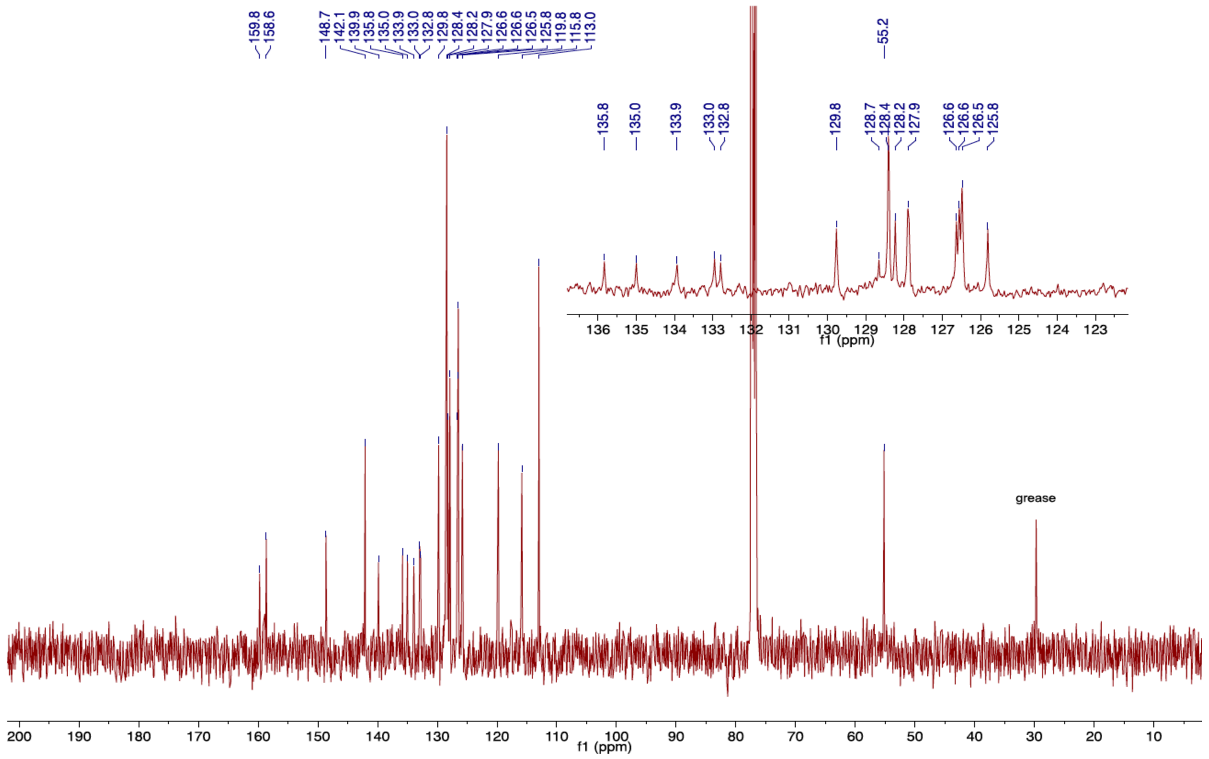

Racemic sample of 3Ab: IA column, Hex:Isop 90:10, T= 30°C, F= 1.0 mL/min.

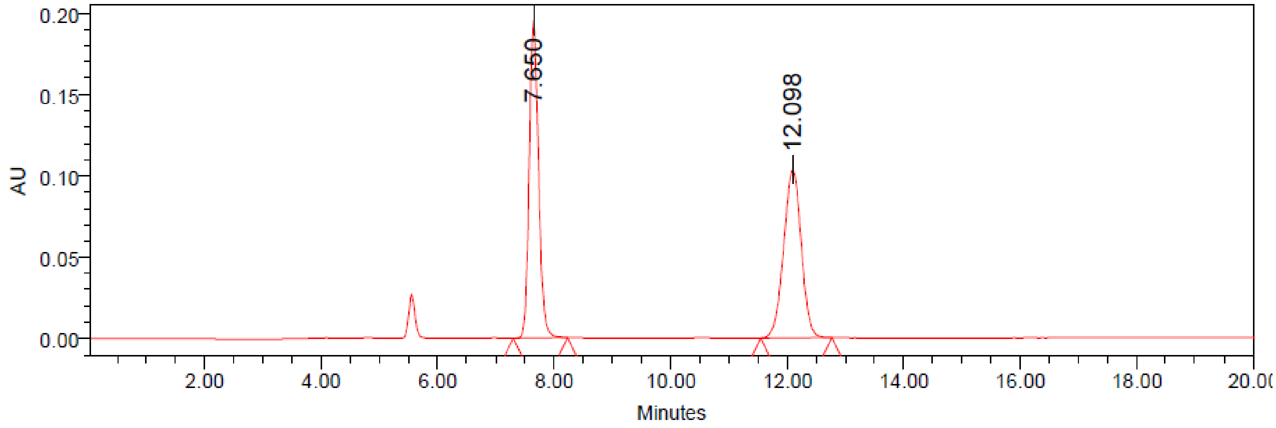

Processed Channel: PDA 327.3 nm

	Processed Channel	Retention Time (min)	Area	% Area	Height
1	PDA 327.3 nm	7.650	2156984	49.98	194853
2	PDA 327.3 nm	12.098	2159072	50.02	103195

Enantioriched sample of 3Ab:

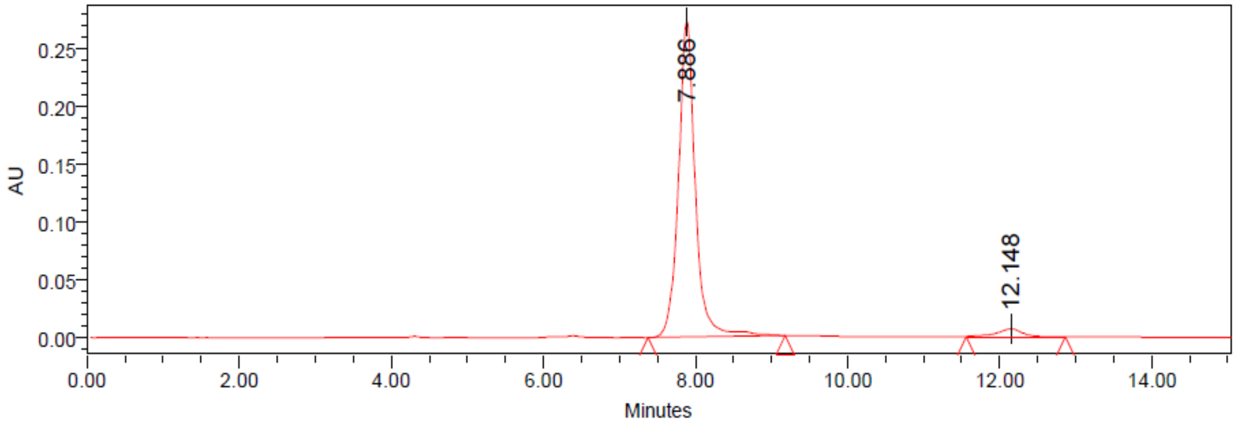

Processed Channel: PDA 257.0 nm

	Processed Channel	Retention Time (min)	Area	% Area	Height
1	PDA 257.0 nm	7.886	4233051	96.55	273734
2	PDA 257.0 nm	12.148	151220	3.45	6637

^{1}H NMR (400MHz, $CDCl_3$) of **3Ac.**

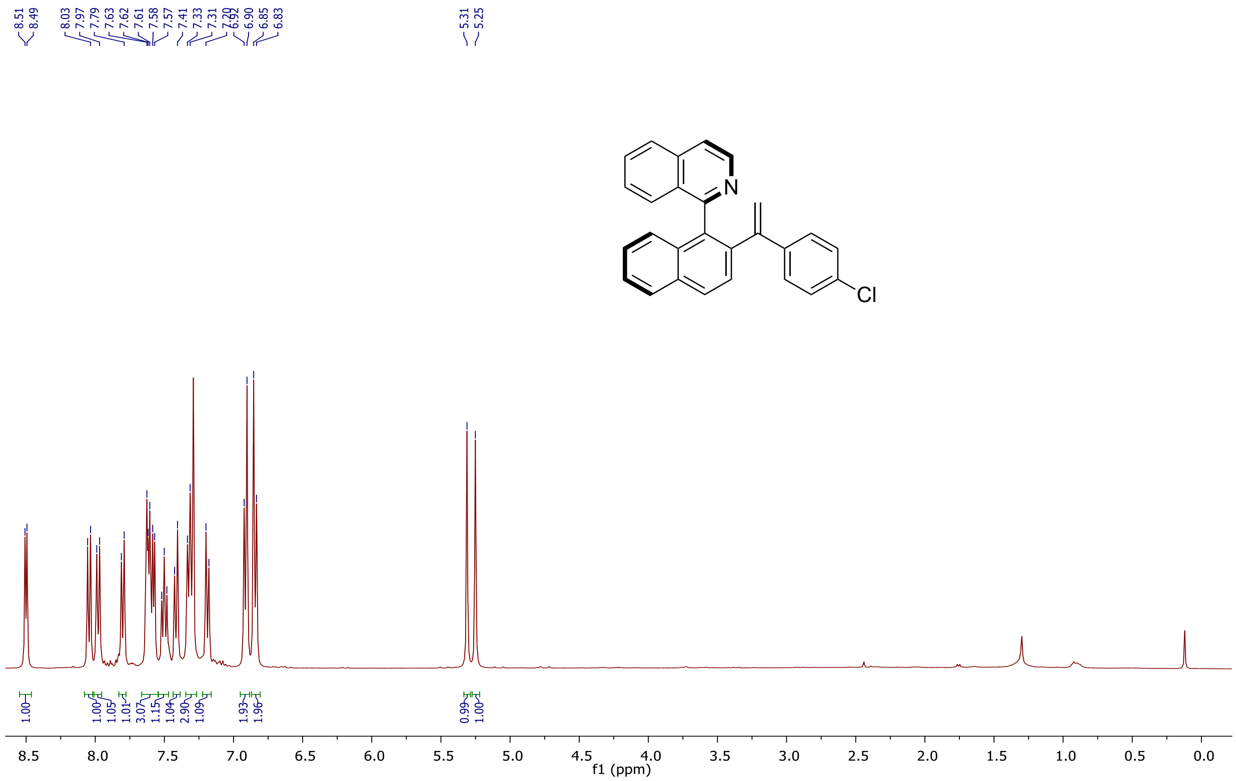

^{13}C NMR (100MHz, $CDCl_3$) of **3Ac.**

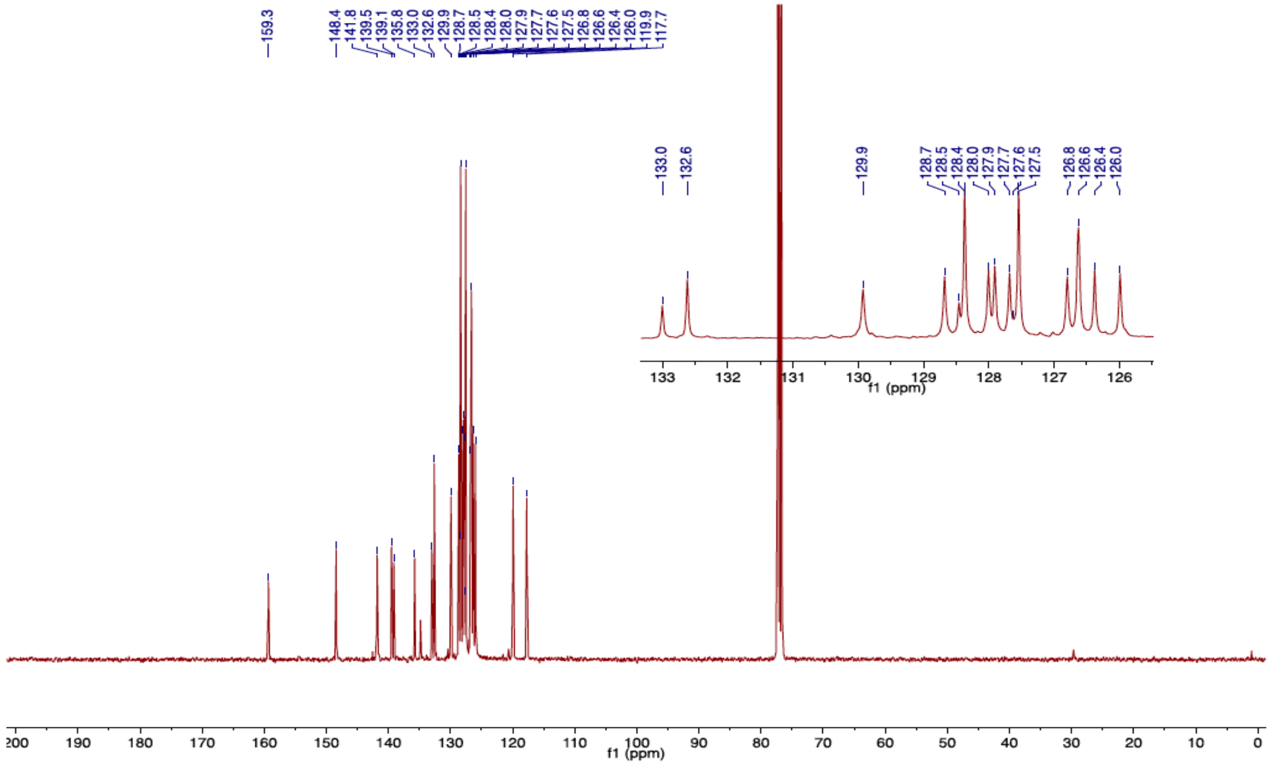

Racemic sample of 3Ac: IA column, Hex:Isop 90:10, T= 30°C, F= 1.0 mL/min.

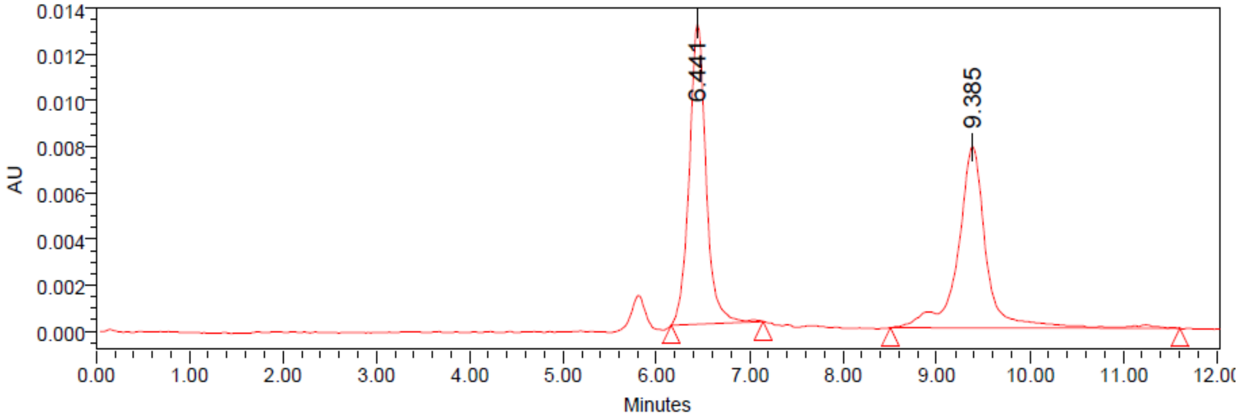

Processed Channel: PDA 338.0 nm

	Processed Channel	Retention Time (min)	Area	% Area	Height
1	PDA 338.0 nm	6.441	162612	49.11	13041
2	PDA 338.0 nm	9.385	168532	50.89	7859

Enantioriched sample of 3Ac:

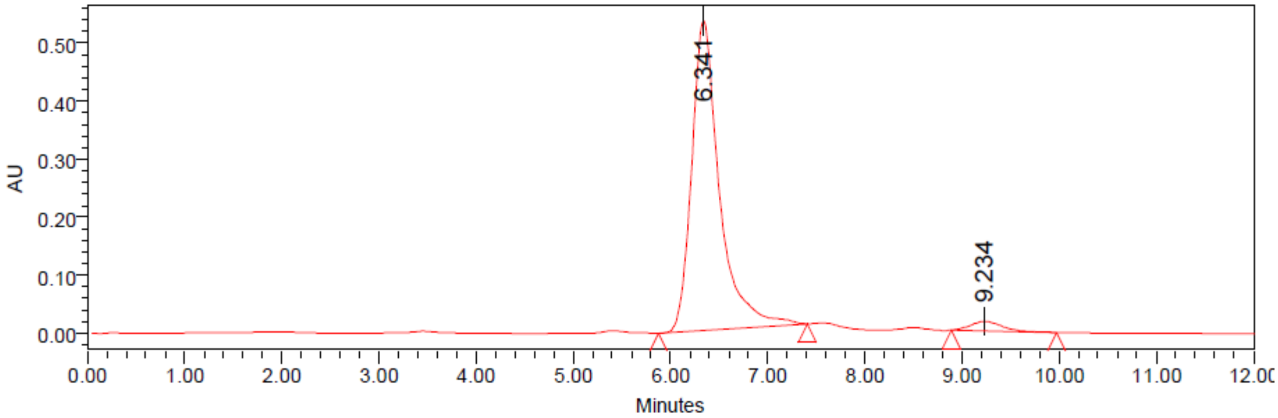

Processed Channel: PDA 253.7 nm

	Processed Channel	Retention Time (min)	Area	% Area	Height
1	PDA 253.7 nm	6.341	10569372	96.60	532949
2	PDA 253.7 nm	9.234	371703	3.40	16501

^{1}H NMR (400MHz, $CDCl_3$) of **3Ad.**

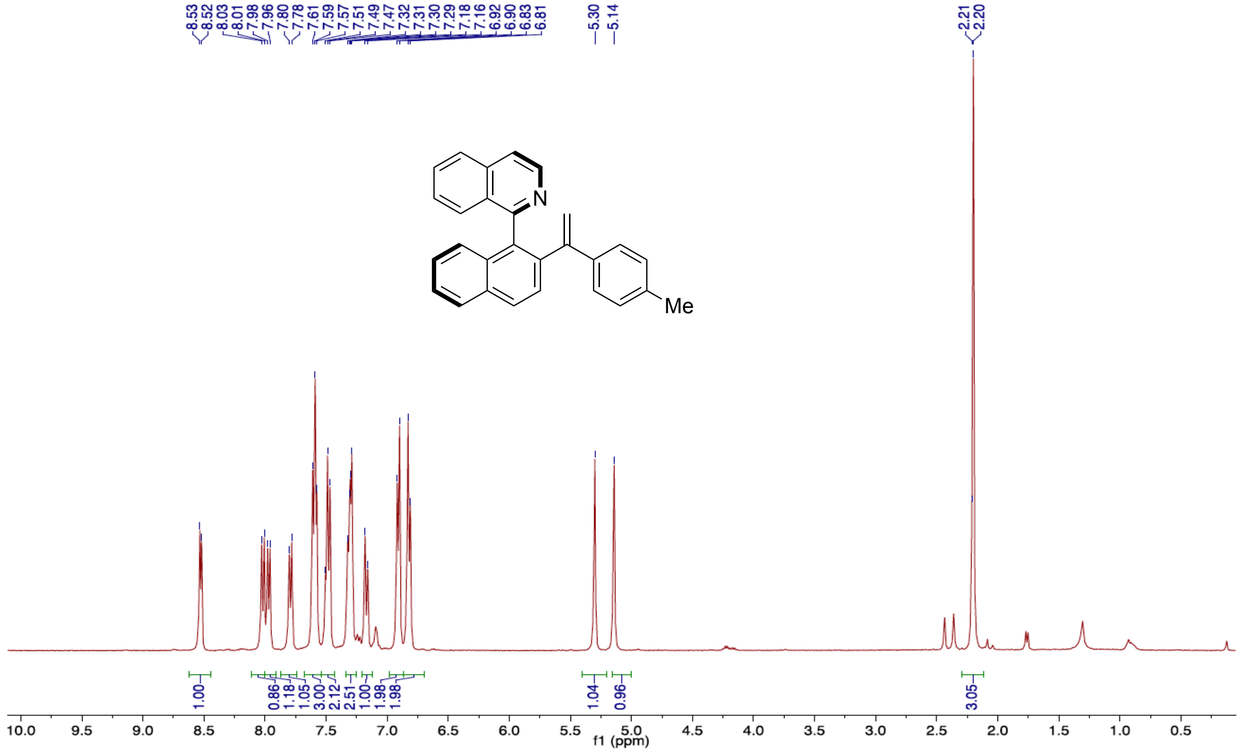

^{13}C NMR (100MHz, $CDCl_3$) of **3Ad.**

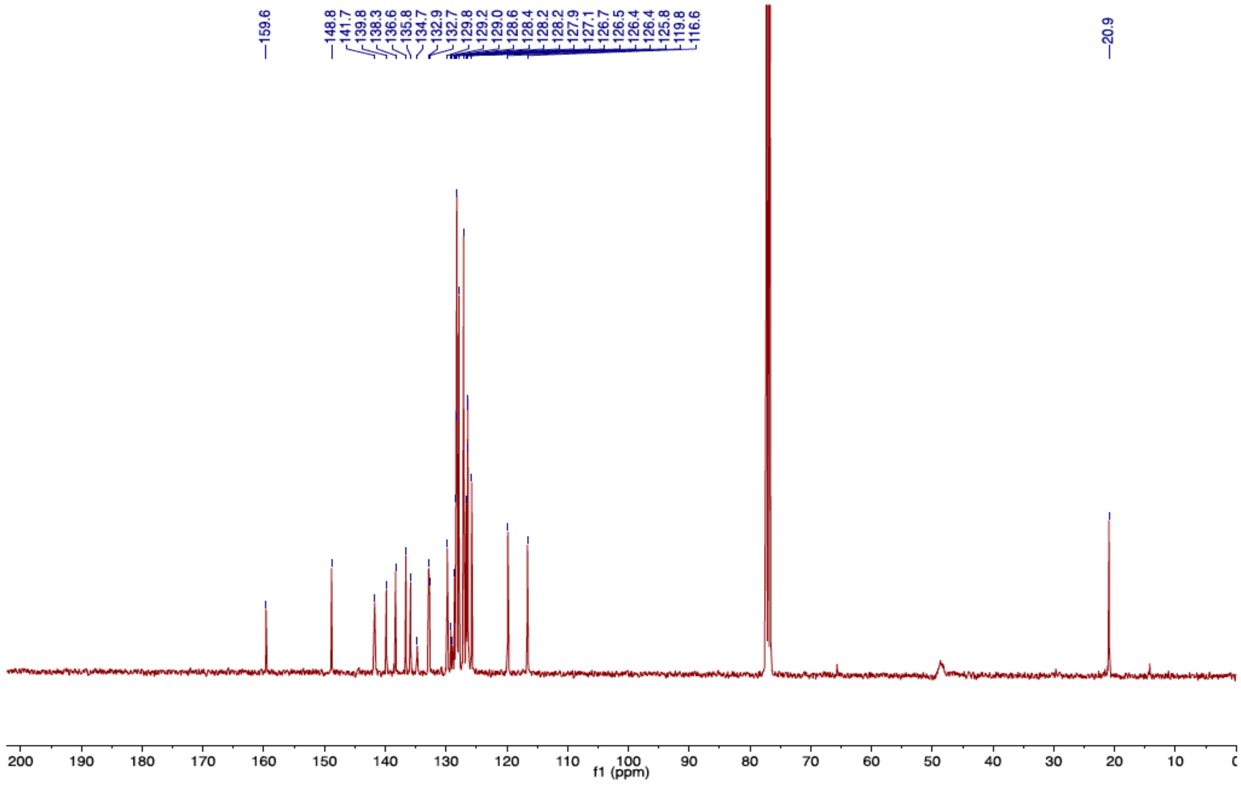

Racemic sample of 3Ad: IA column, Hex:Isop 90:10, T= 30°C, F= 1.0 mL/min.

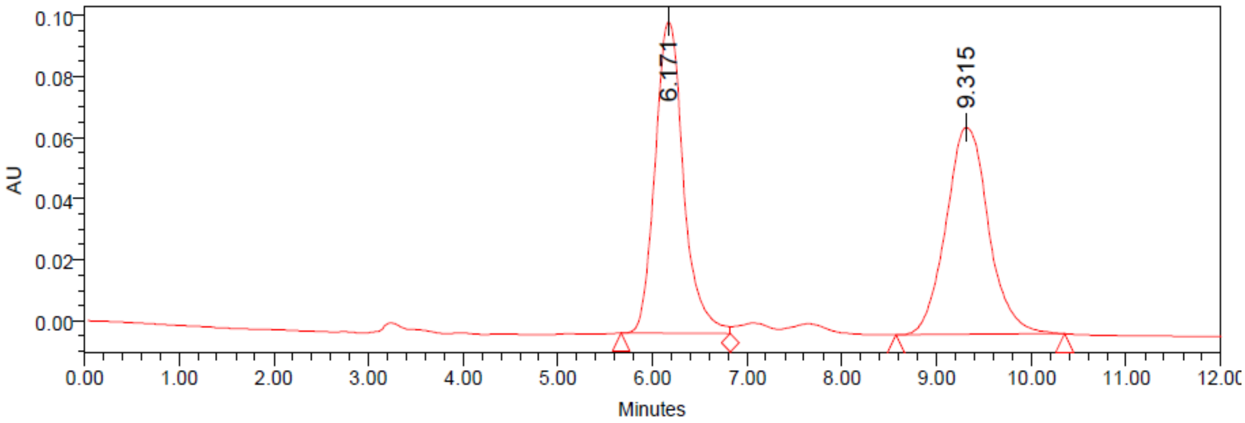

Processed Channel: PDA 221.5 nm

	Processed Channel	Retention Time (min)	Area	% Area	Height
1	PDA 221.5 nm	6.171	2131977	50.08	101951
2	PDA 221.5 nm	9.315	2125208	49.92	67830

Enantioriched sample of 3Ad:

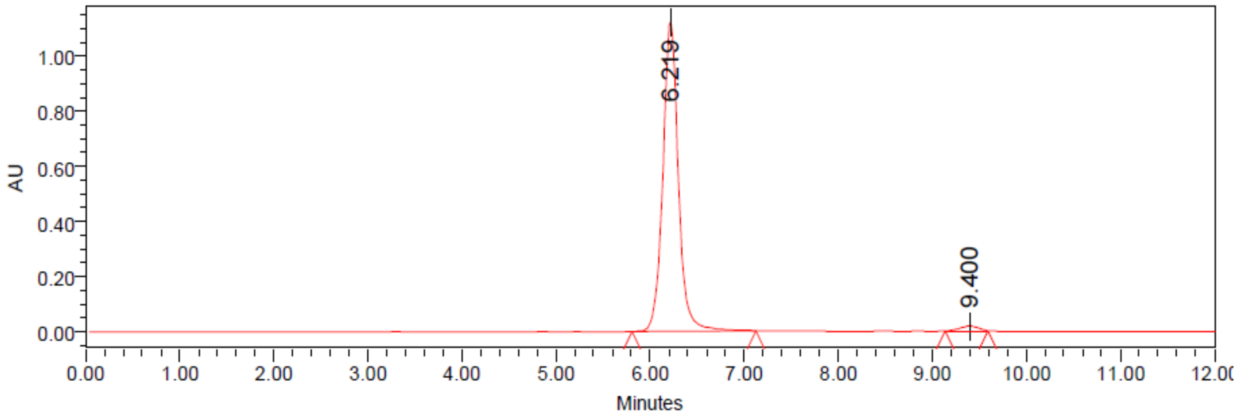

Processed Channel: PDA 248.0 nm

	Processed Channel	Retention Time (min)	Area	% Area	Height
1	PDA 248.0 nm	6.219	12780098	98.30	1123951
2	PDA 248.0 nm	9.400	221031	1.70	16640

^{1}H NMR (400MHz, $CDCl_3$) of **3Ae.**

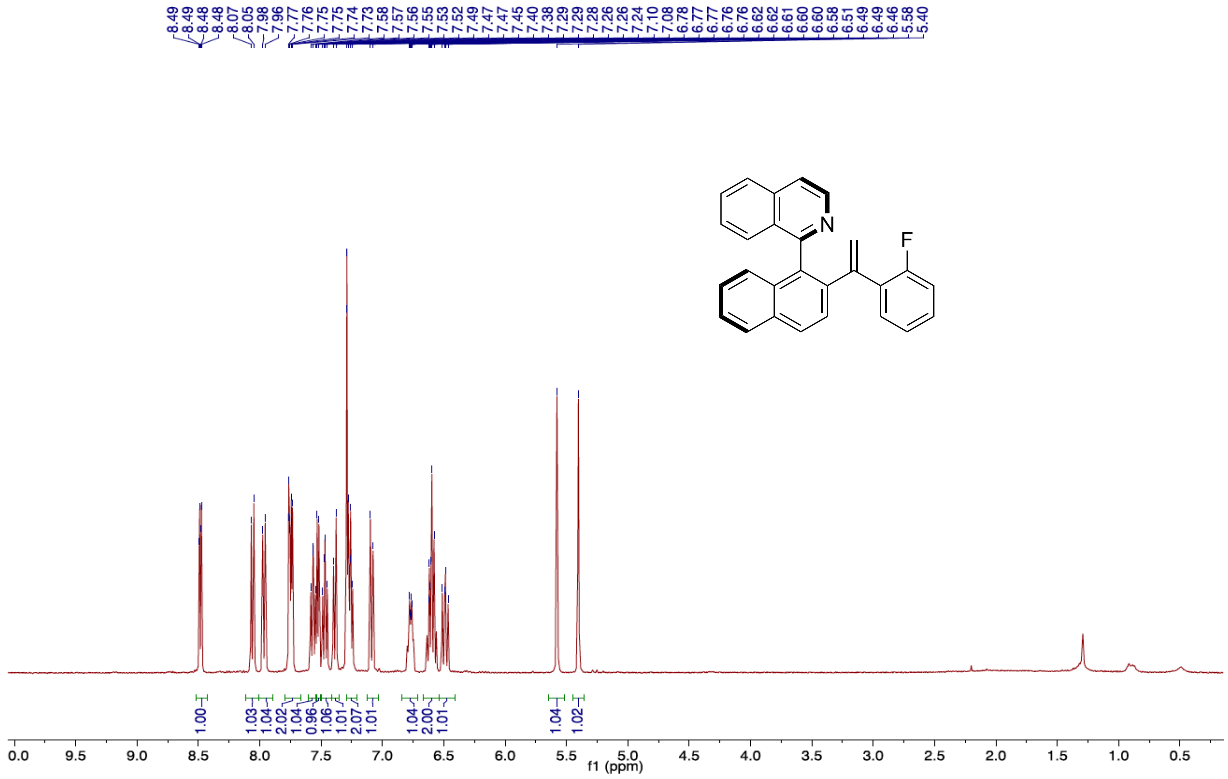

^{13}C NMR (100MHz, $CDCl_3$) of **3Ae.**

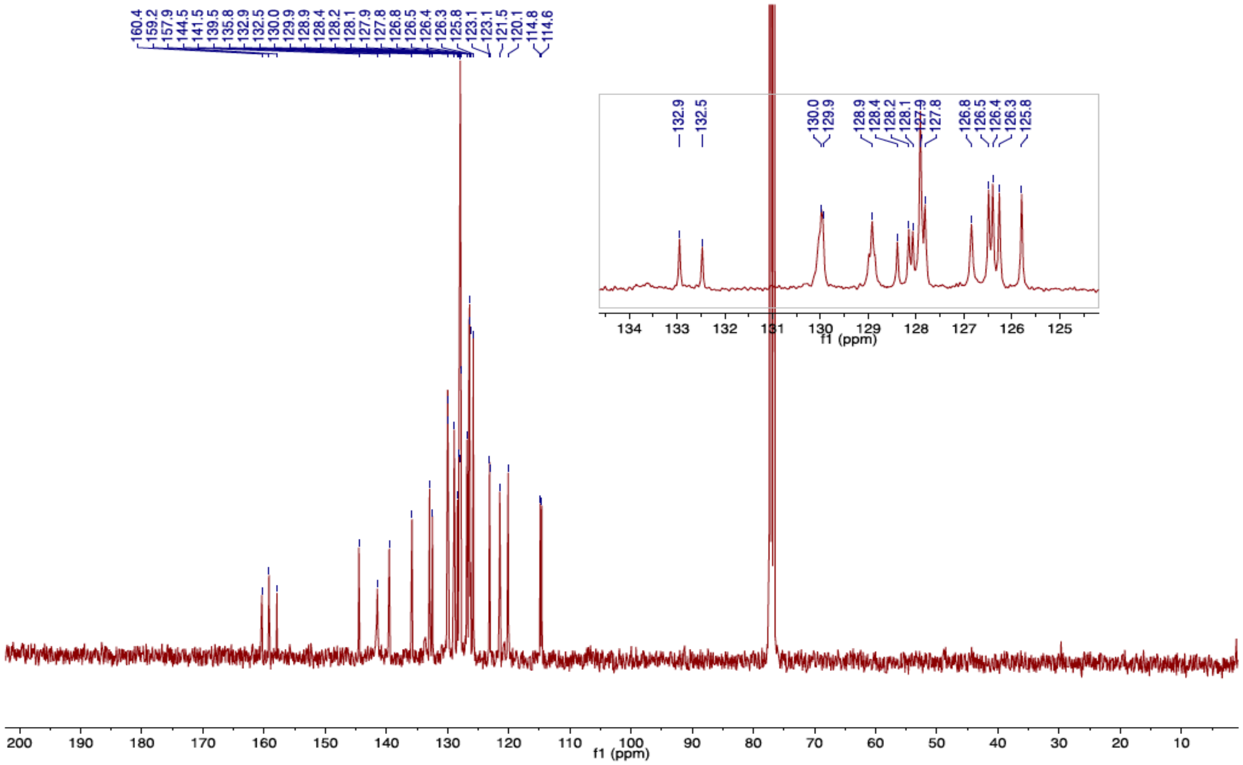

^{19}F NMR (377 MHz, $CDCl_3$) of **3Ae.**

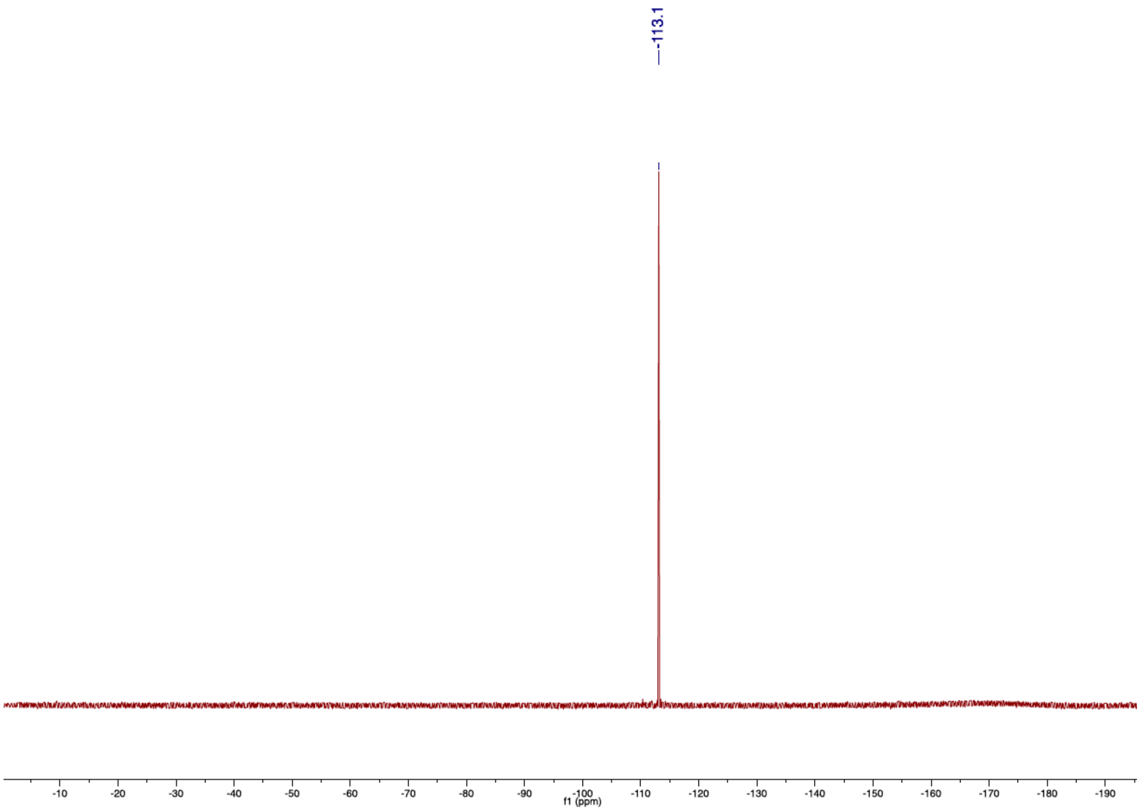

Racemic sample of 3Ae: IA column, Hex:Isop 90:10, T= 30°C, F= 1.0 mL/min.

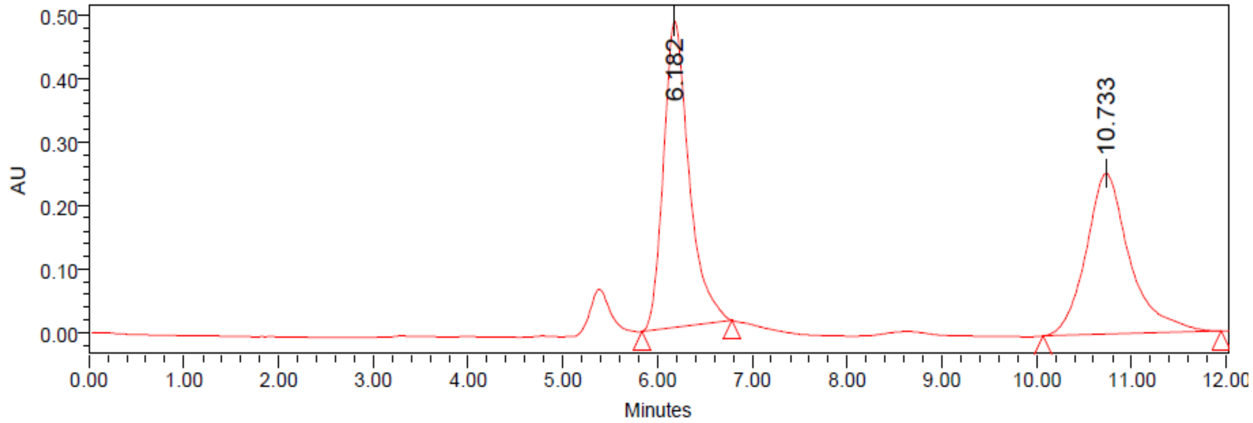

Processed Channel: PDA 221.5 nm

	Processed Channel	Retention Time (min)	Area	% Area	Height
1	PDA 221.5 nm	6.182	9099704	52.91	483412
2	PDA 221.5 nm	10.733	8098377	47.09	253431

Enantioriched sample of 3Ae:

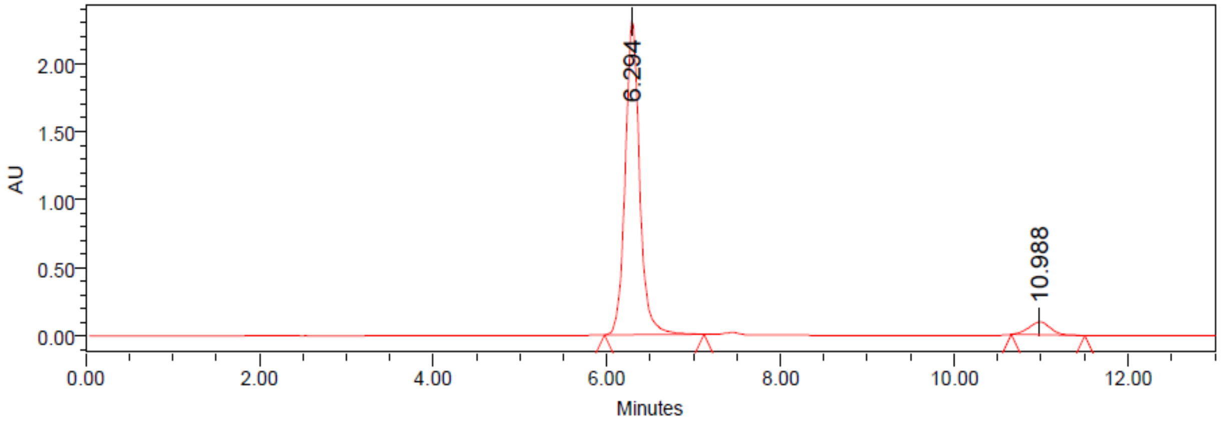

Processed Channel: PDA 241.9 nm

	Processed Channel	Retention Time (min)	Area	% Area	Height
1	PDA 241.9 nm	6.294	26549577	94.23	2304850
2	PDA 241.9 nm	10.988	1625759	5.77	94514

^{1}H NMR (400MHz, $CDCl_3$) of **3Af.**

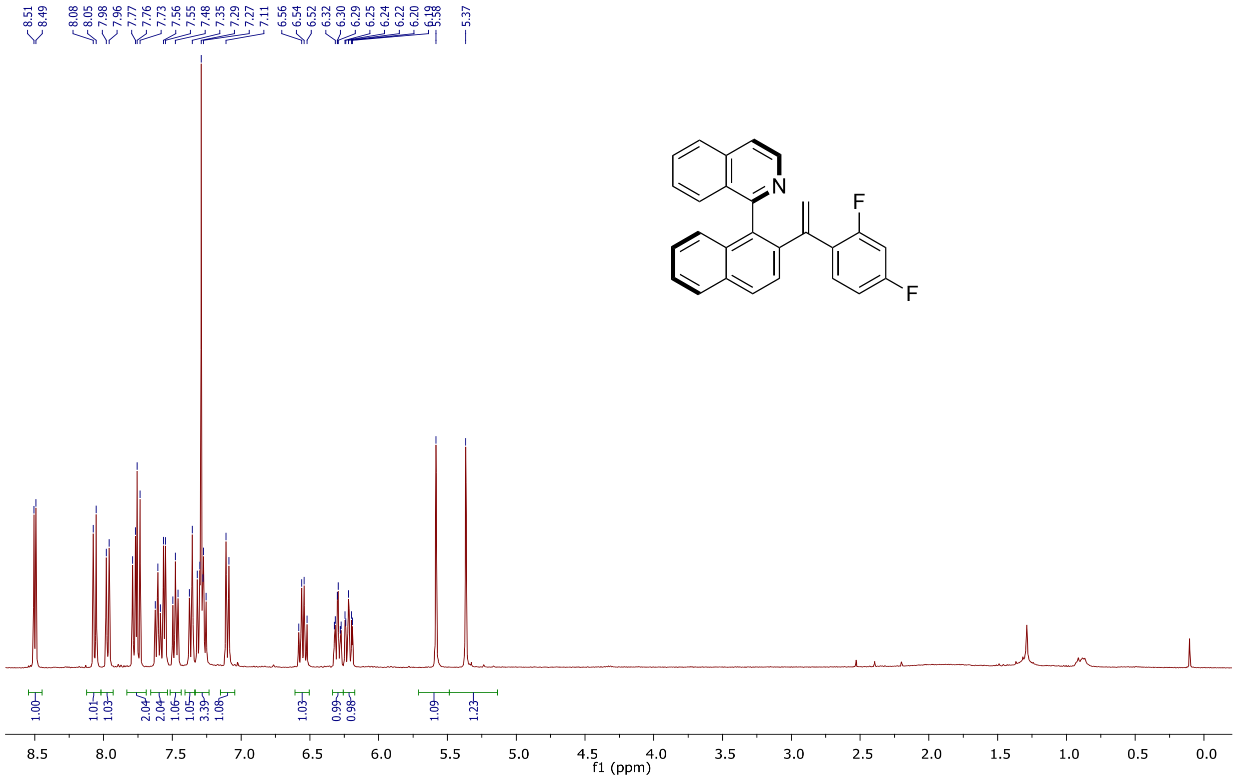

^{13}C NMR (100 MHz, $CDCl_3$) of **3Af.**

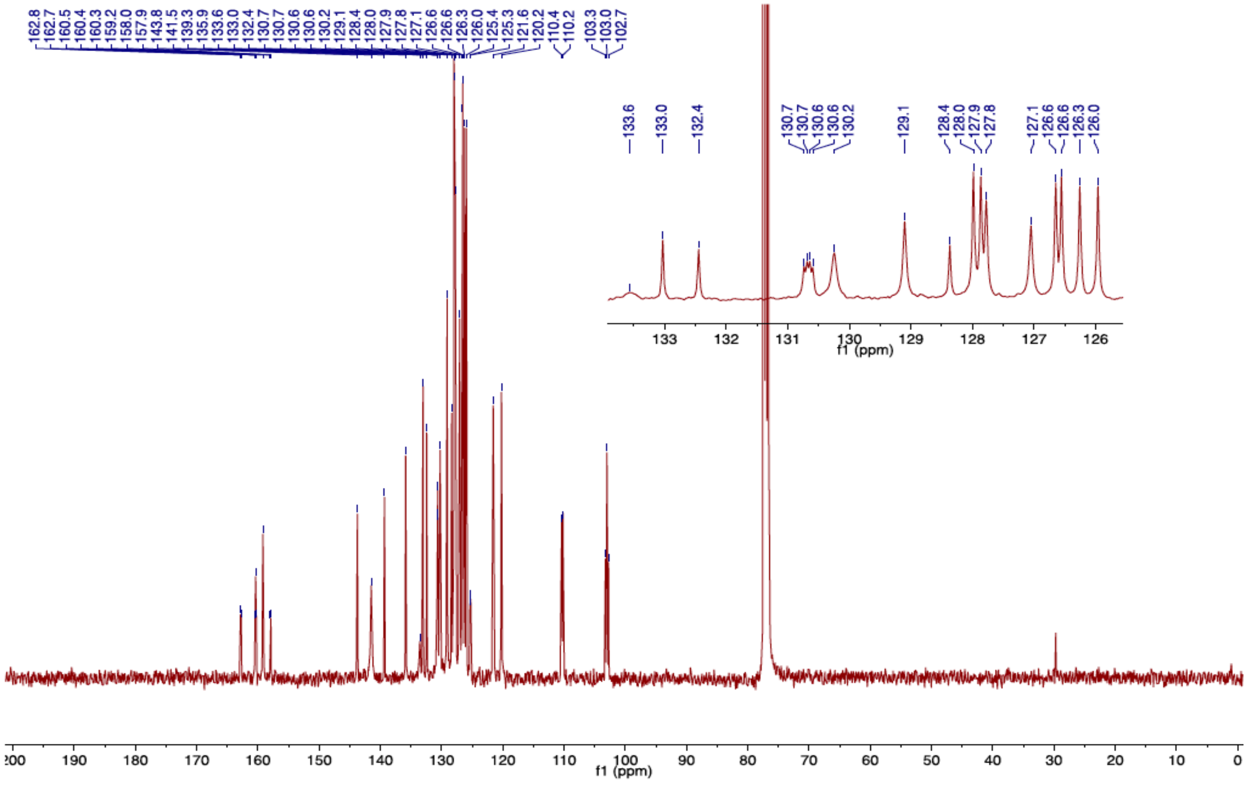

^{19}F NMR (377 MHz, $CDCl_3$) of **3Af.**

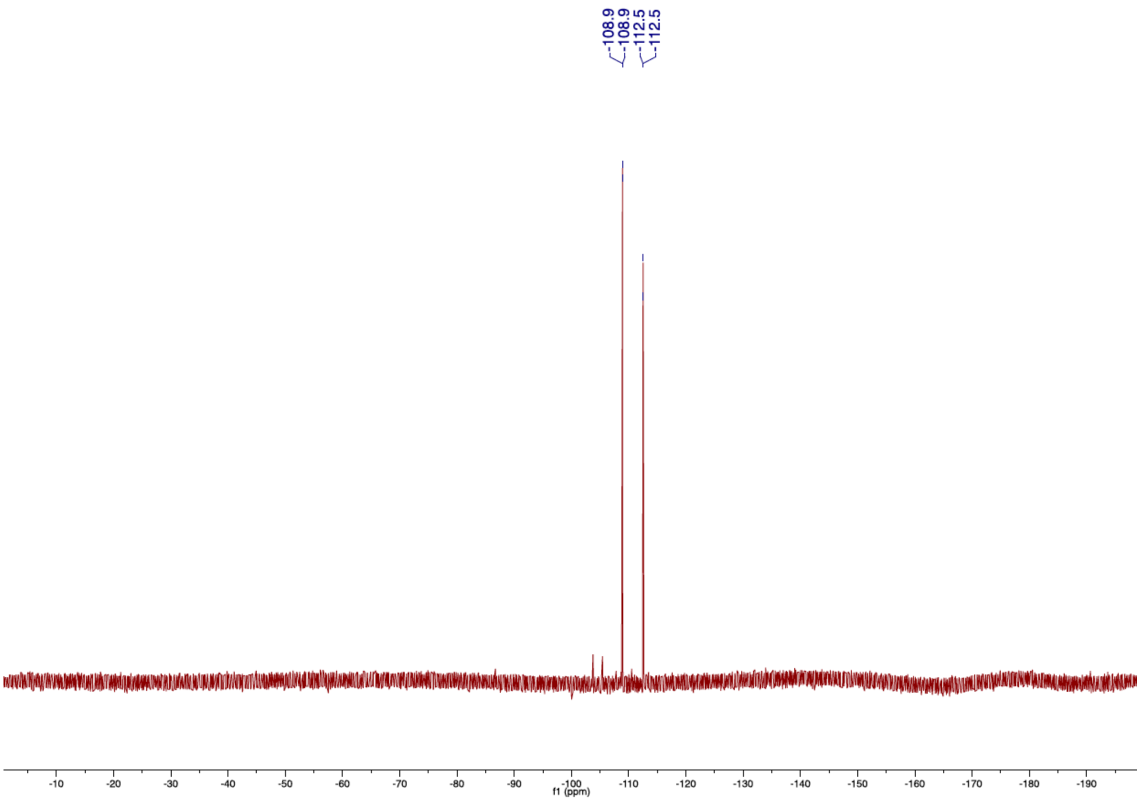

Racemic sample of 3Af: IA column, Hex:Isop 90:10, T= 30°C, F= 1.0 mL/min.

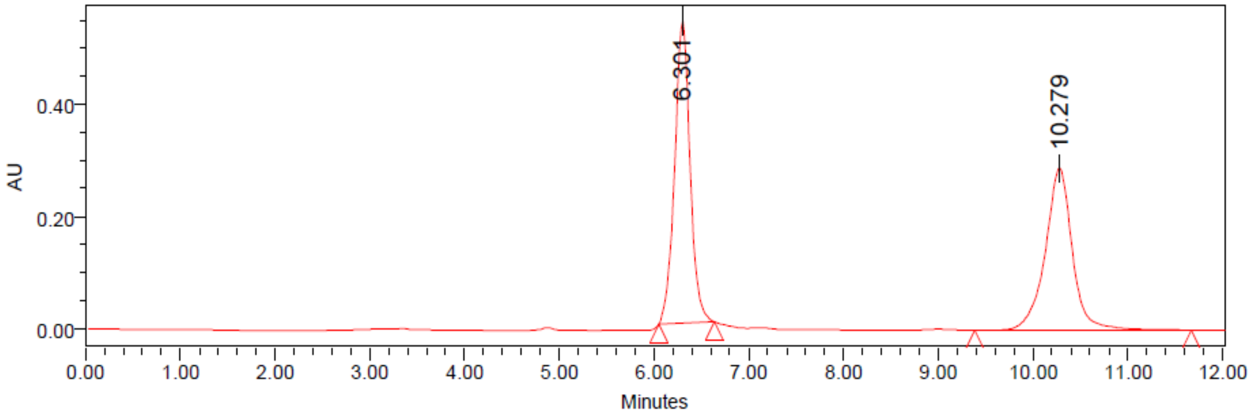

Processed Channel: PDA 221.5 nm

	Processed Channel	Retention Time (min)	Area	% Area	Height
1	PDA 221.5 nm	6.301	6095060	51.46	537858
2	PDA 221.5 nm	10.279	5748446	48.54	288945

Enantioriched sample of 3Af:

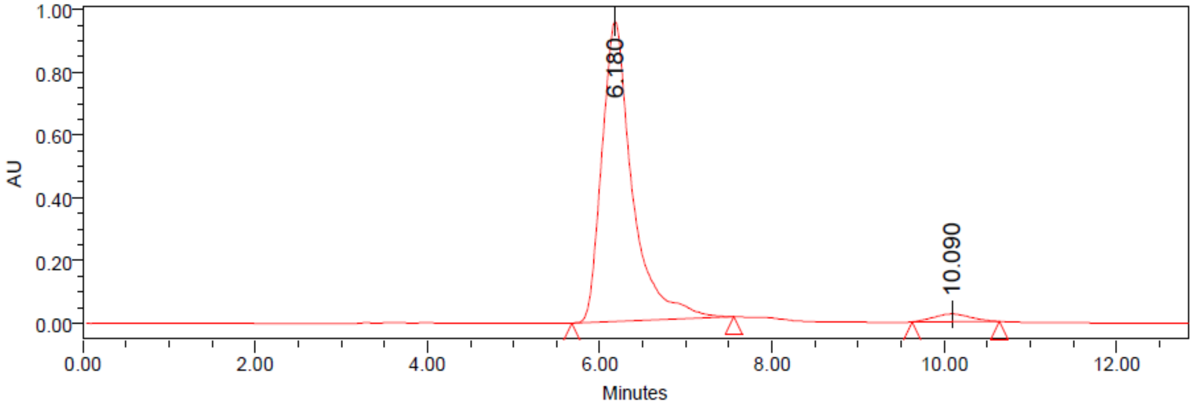

Processed Channel: PDA 209.7 nm

	Processed Channel	Retention Time (min)	Area	% Area	Height
1	PDA 209.7 nm	6.180	23465760	96.98	955043
2	PDA 209.7 nm	10.090	729538	3.02	24418

^{1}H NMR (400MHz, $CDCl_3$) of **3Ag.**

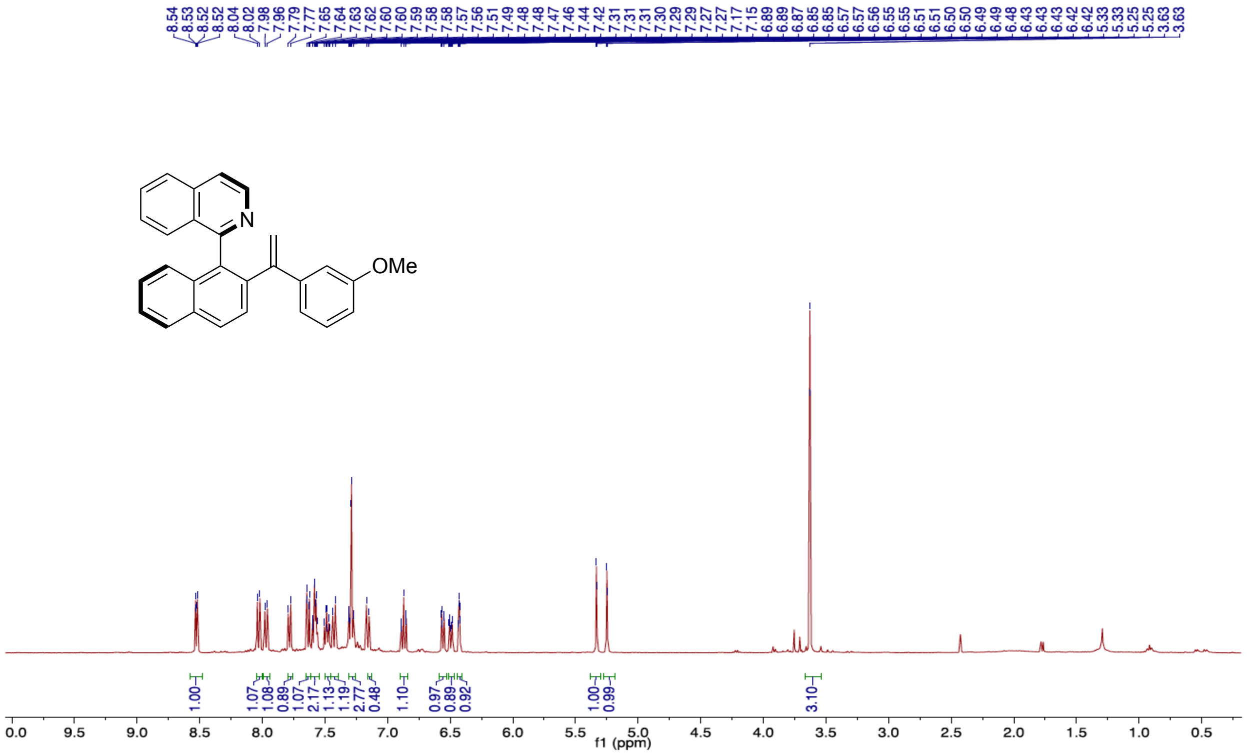

^{13}C NMR (100MHz, $CDCl_3$) of **3Ag.**

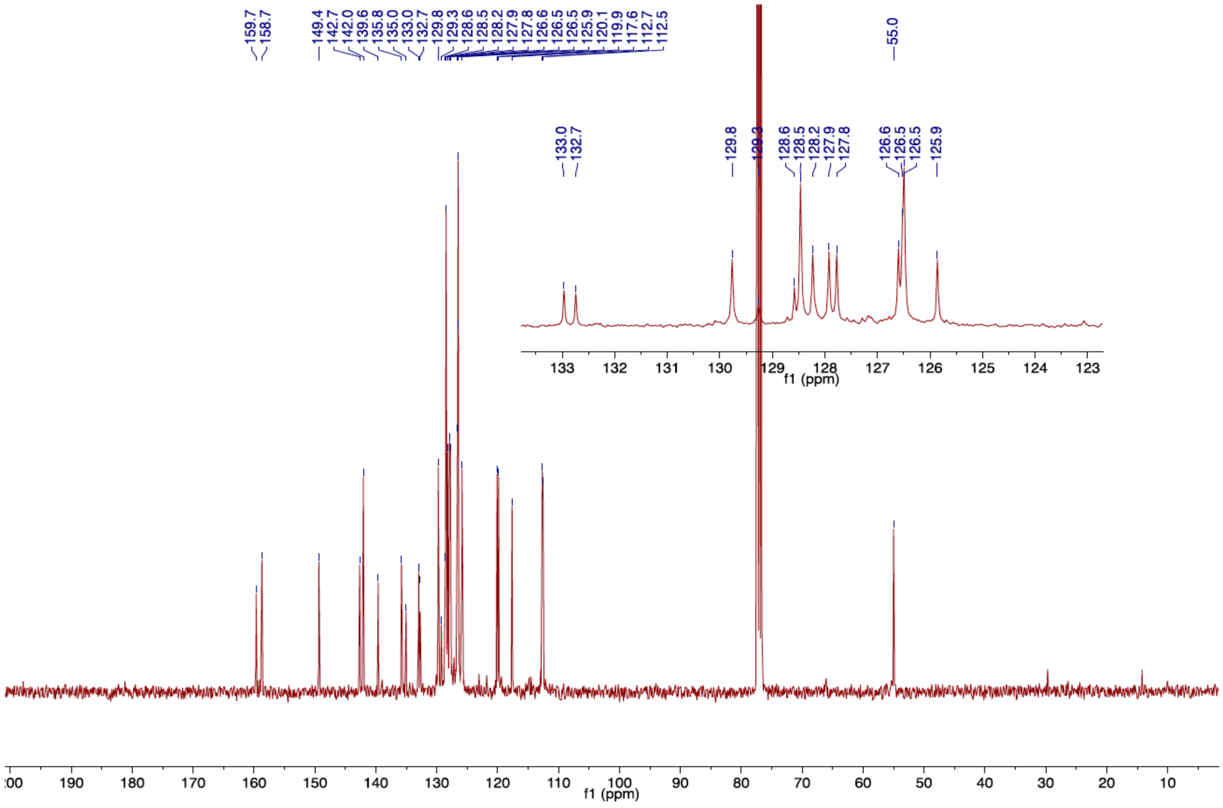

Racemic sample of 3Ag: IA column, Hex:Isop 90:10, T= 30°C, F= 1.0 mL/min.

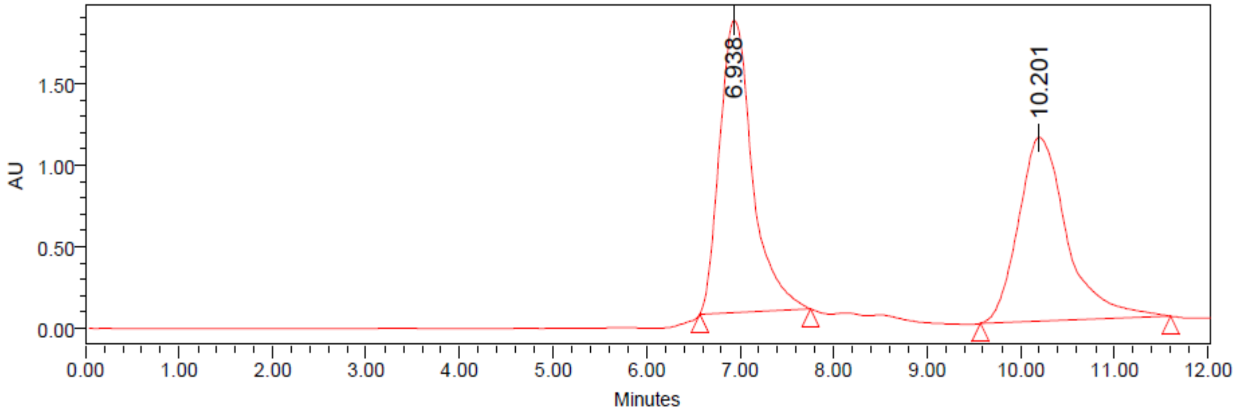

Processed Channel: PDA 221.5 nm

	Processed Channel	Retention Time (min)	Area	% Area	Height
1	PDA 221.5 nm	6.938	43838548	51.81	1790838
2	PDA 221.5 nm	10.201	40769622	48.19	1125556

Enantioriched sample of 3Ag:

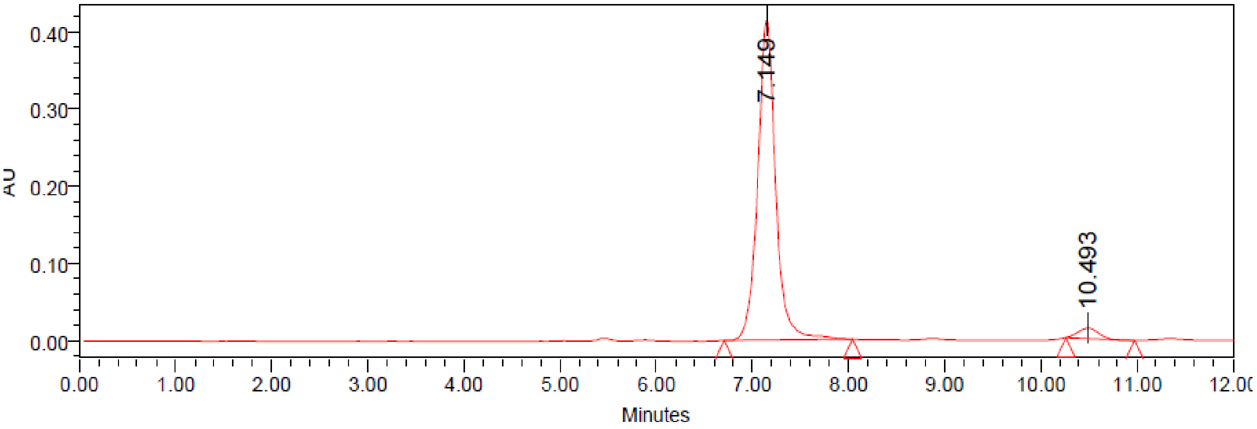

Processed Channel: PDA 259.1 nm

	Processed Channel	Retention Time (min)	Area	% Area	Height
1	PDA 259.1 nm	7.149	5382041	96.53	414488
2	PDA 259.1 nm	10.493	193511	3.47	13102

^{1}H NMR (400MHz, $CDCl_3$) of **3Ba.**

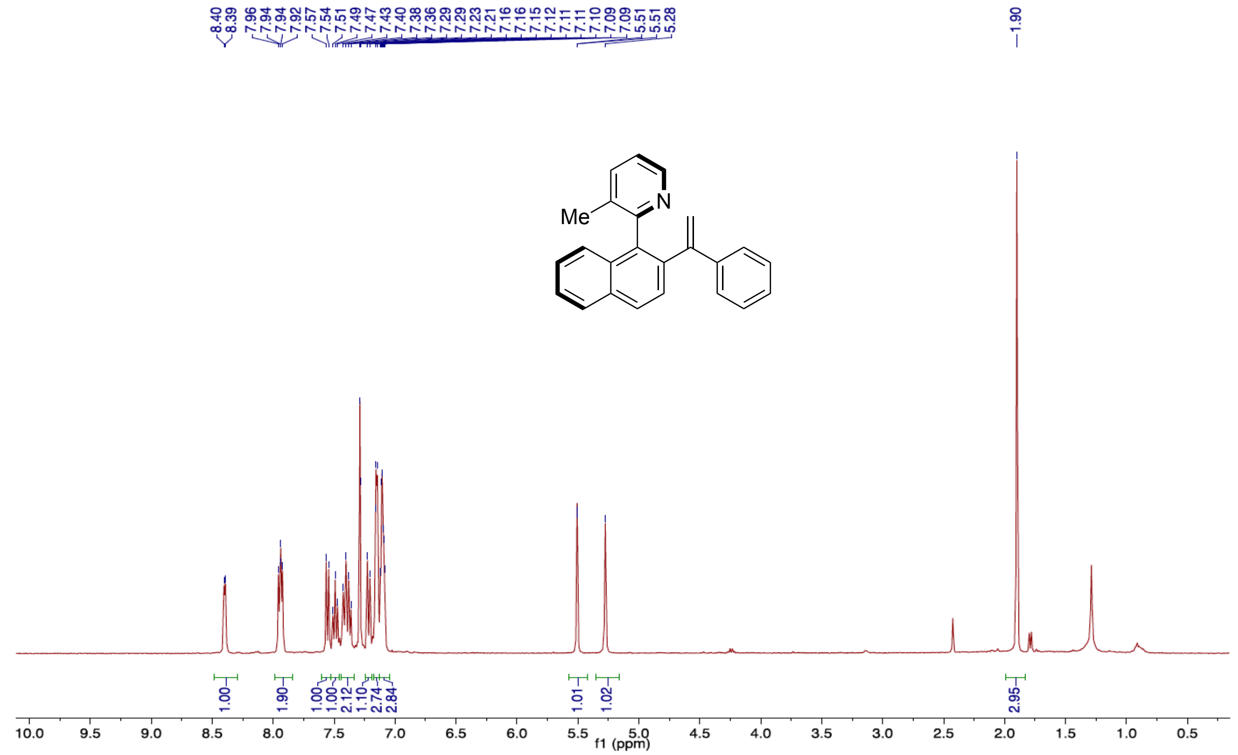

^{13}C NMR (100MHz, $CDCl_3$) of **3Ba.**

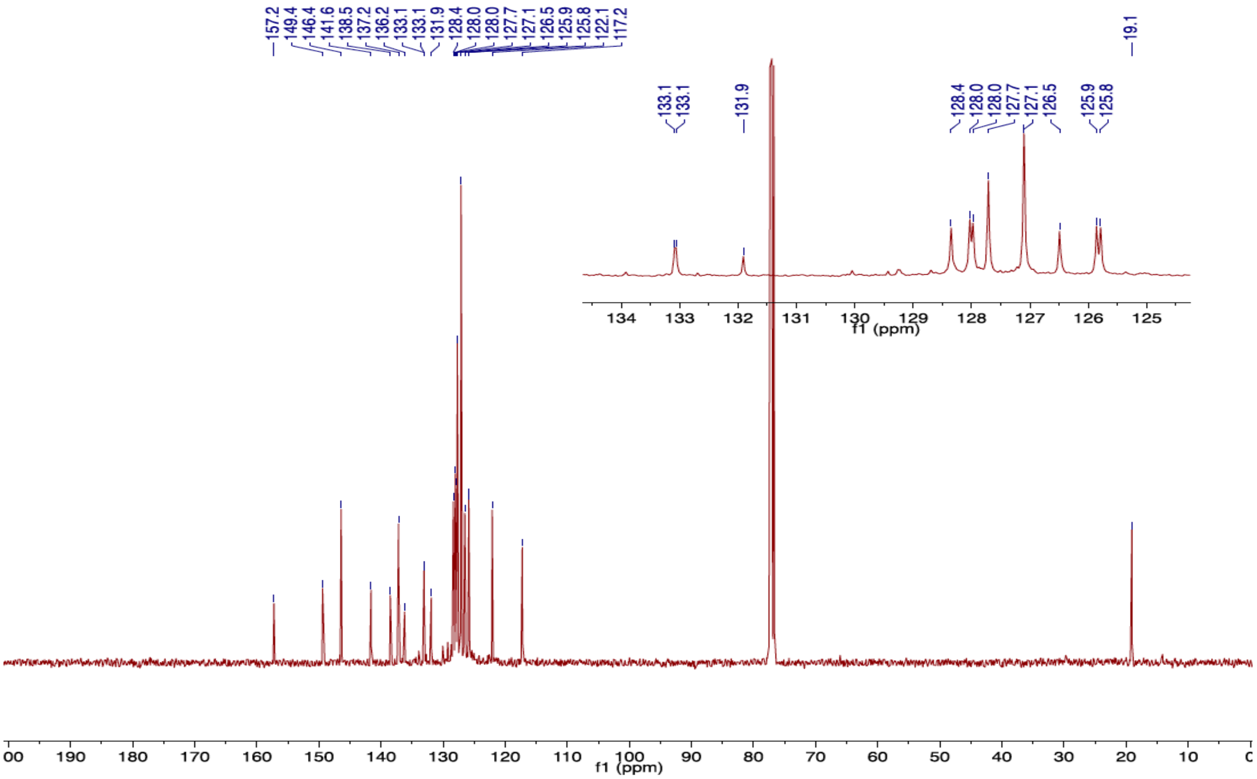

Racemic sample of 3Ba: IA column, Hex:Isop 90:10, T= 30°C, F= 1.0 mL/min.

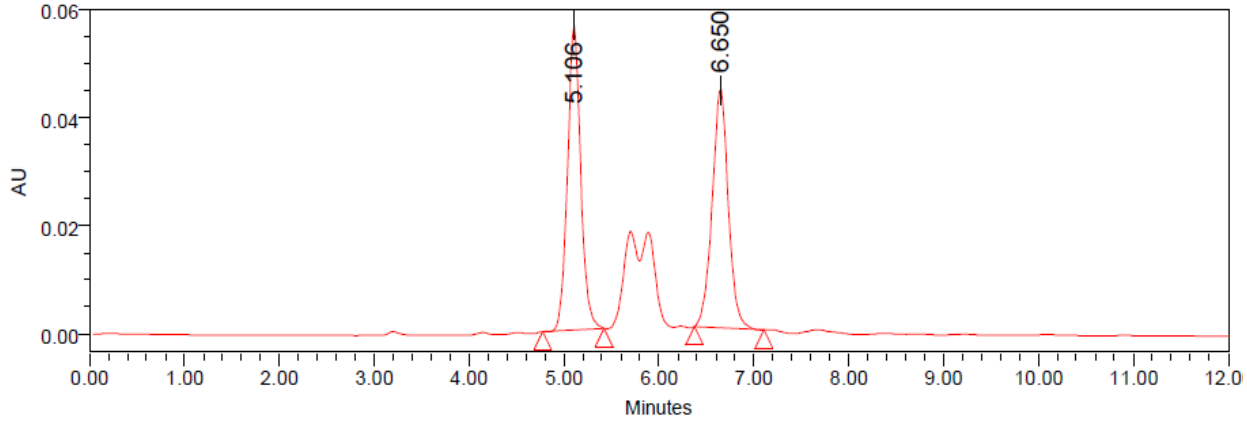

Processed Channel: PDA 244.5 nm

	Processed Channel	Retention Time (min)	Area	% Area	Height
1	PDA 244.5 nm	5.106	540954	50.76	56324
2	PDA 244.5 nm	6.650	524794	49.24	44239

Enantioriched sample of 3Ba:

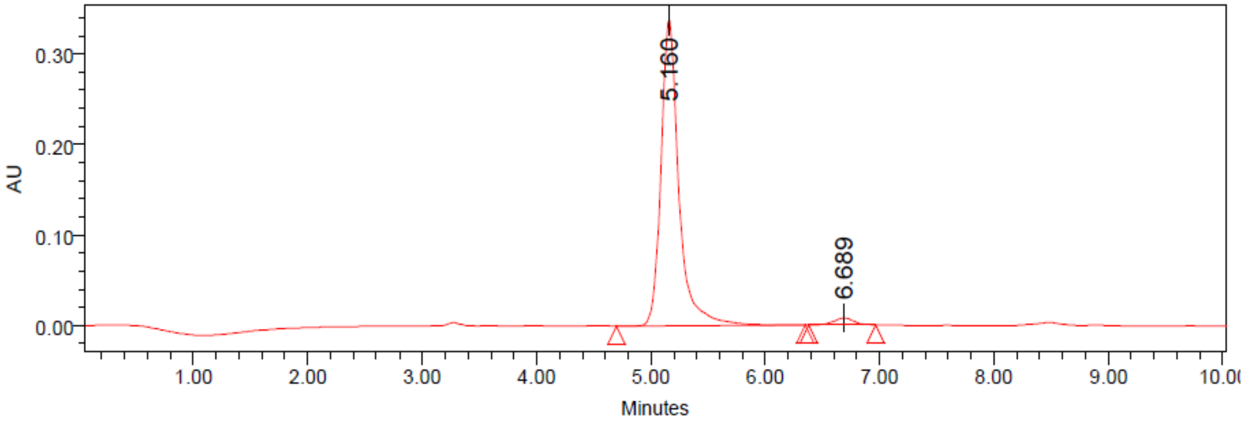

Processed Channel: PDA 221.5 nm

	Processed Channel	Retention Time (min)	Area	% Area	Height
1	PDA 221.5 nm	5.160	3687064	97.46	339080
2	PDA 221.5 nm	6.689	96103	2.54	7728

^{1}H NMR (400MHz, $CDCl_3$) of **3Be.**

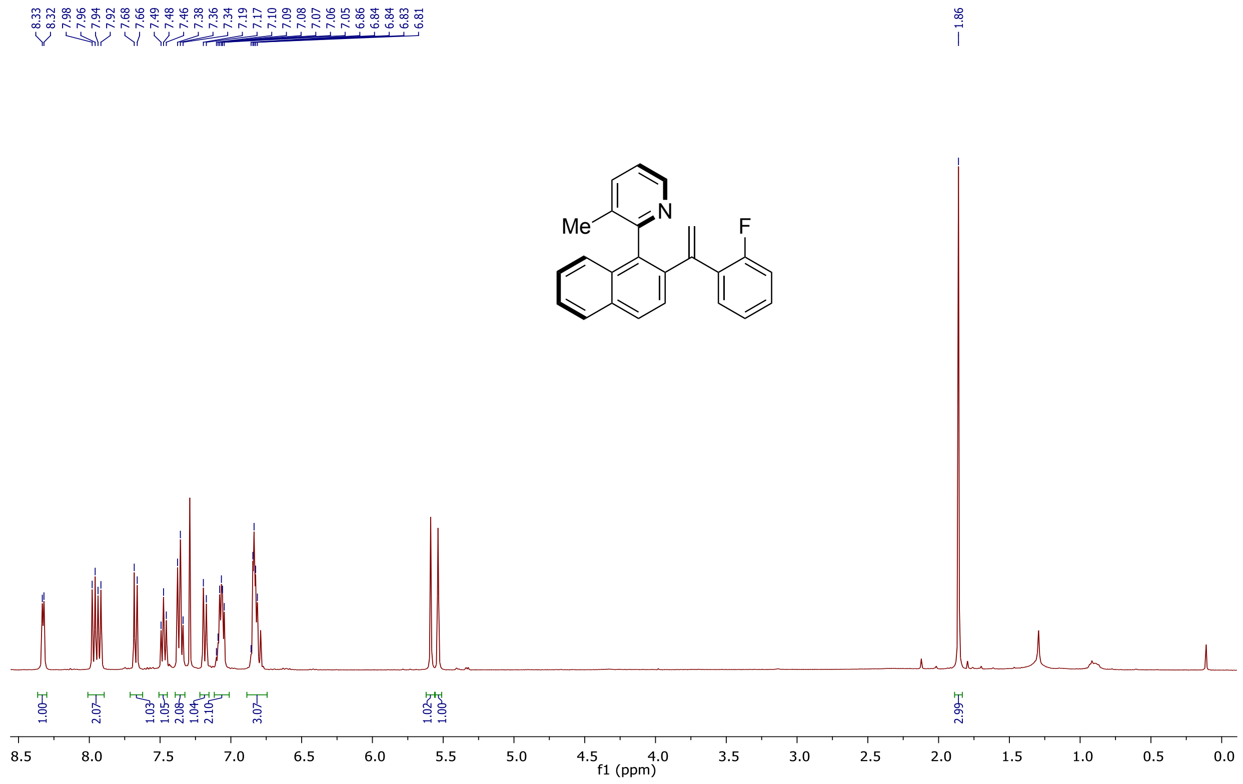

^{13}C NMR (100MHz, $CDCl_3$) of **3Be.**

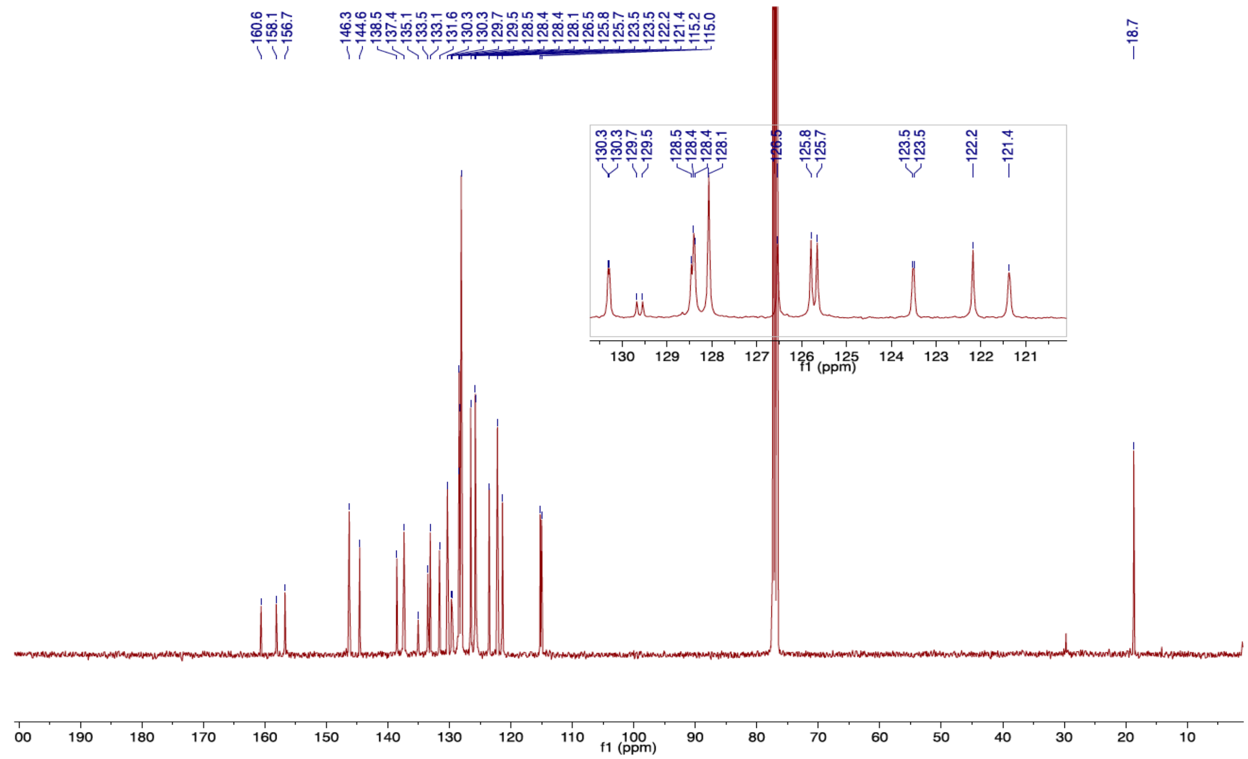

^{19}F NMR (377 MHz, $CDCl_3$) of **3Be:**

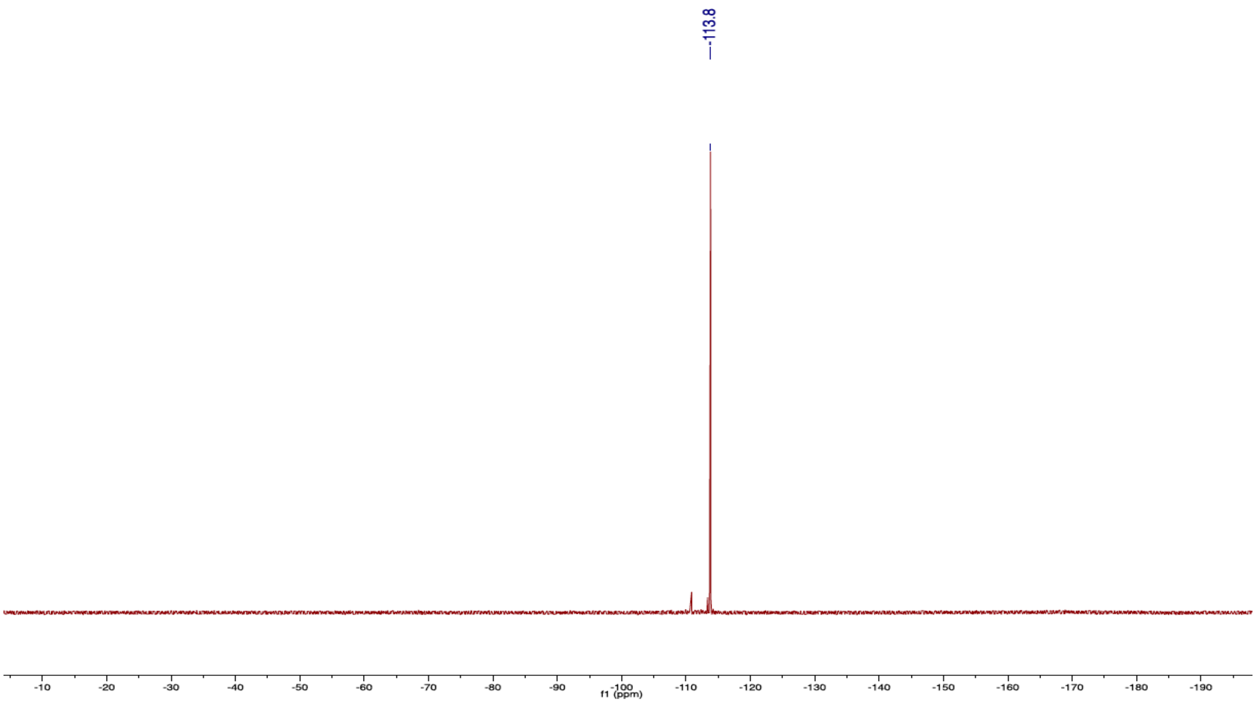

Racemic sample of 3Be: IA column, Hex:Isop 90:10, T= 30°C, F= 1.0 mL/min.

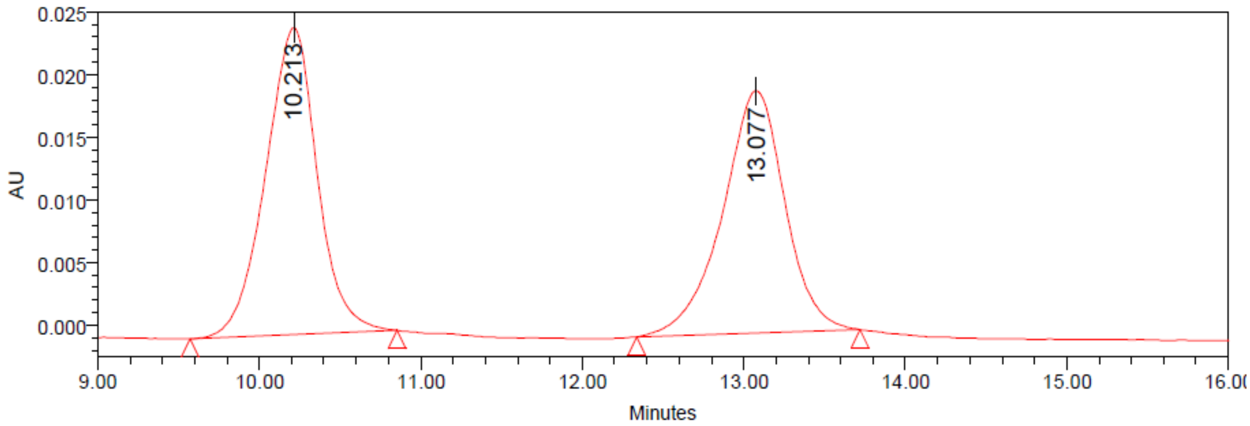

Processed Channel: PDA 221.5 nm

	Processed Channel	Retention Time (min)	Area	% Area	Height
1	PDA 221.5 nm	10.213	545994	51.30	24549
2	PDA 221.5 nm	13.077	518293	48.70	19334

Enantioriched sample of 3Be:

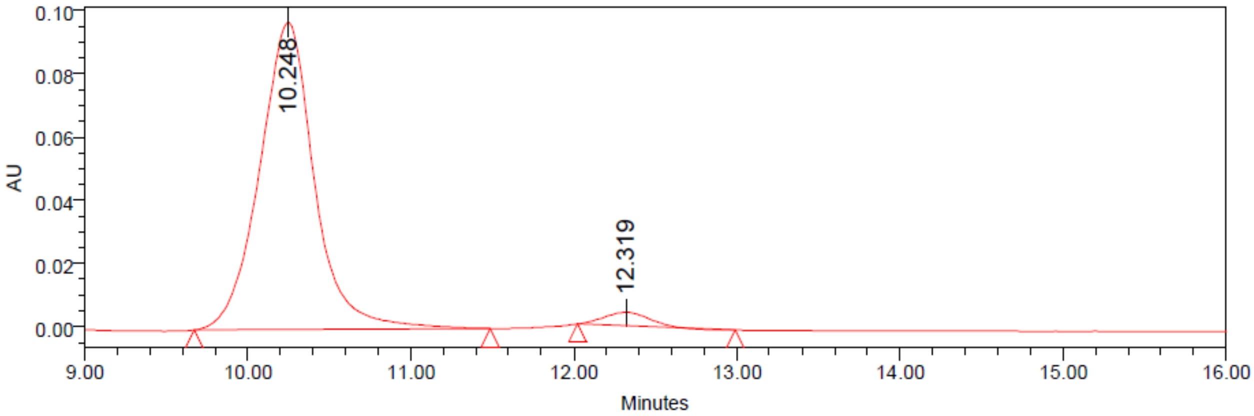

Processed Channel: PDA 221.5 nm

	Processed Channel	Retention Time (min)	Area	% Area	Height
1	PDA 221.5 nm	10.248	2272875	96.39	97225
2	PDA 221.5 nm	12.319	85169	3.61	4274

^{1}H NMR (400MHz, $CDCl_3$) of **3Bg.**

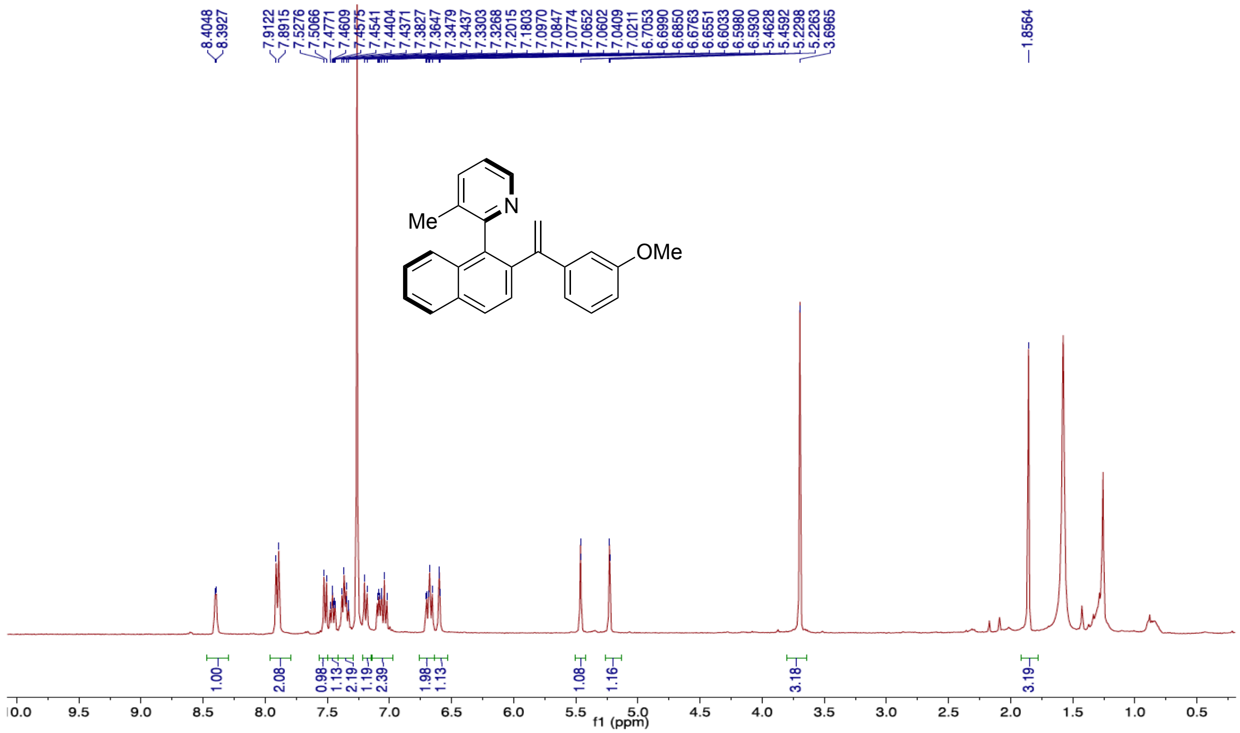

^{13}C NMR (100MHz, $CDCl_3$) of **3Bg.**

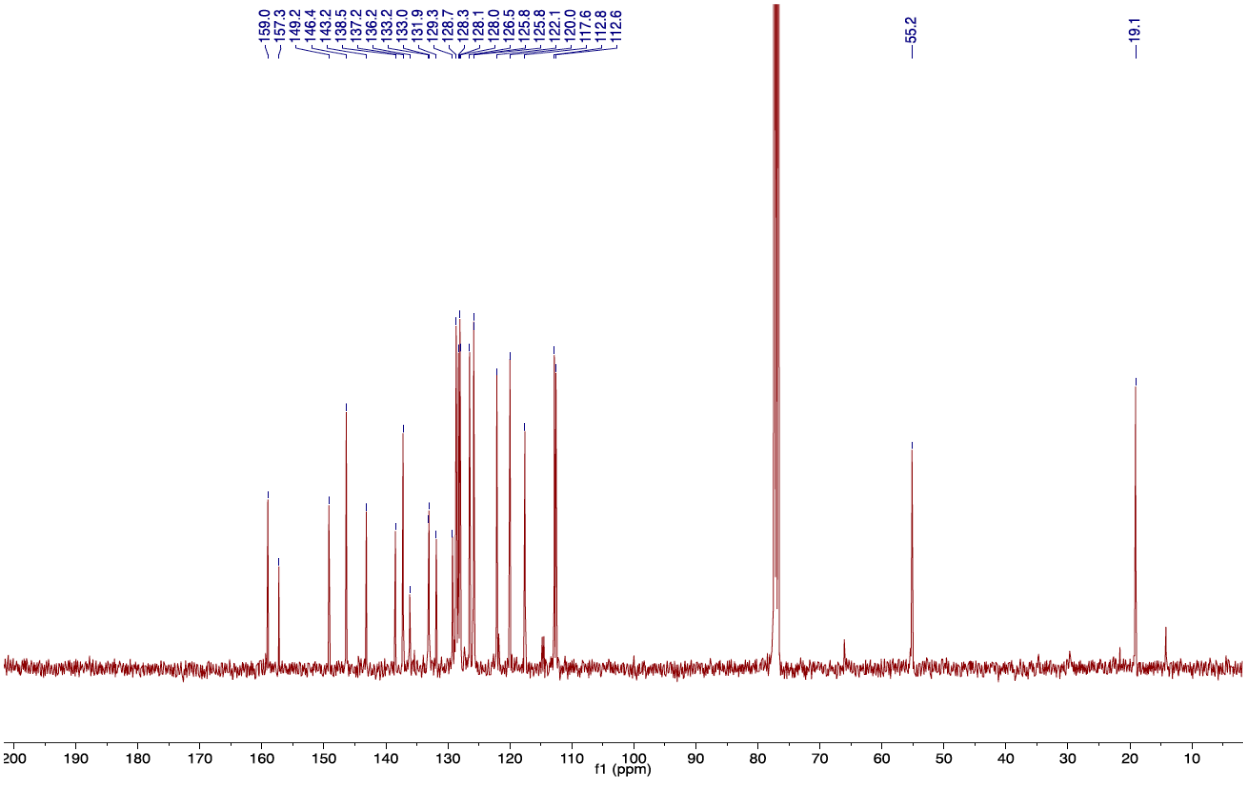

Racemic sample of 3Bg: IA column, Hex:Isop 90:10, T= 30°C, F= 1.0 mL/min.

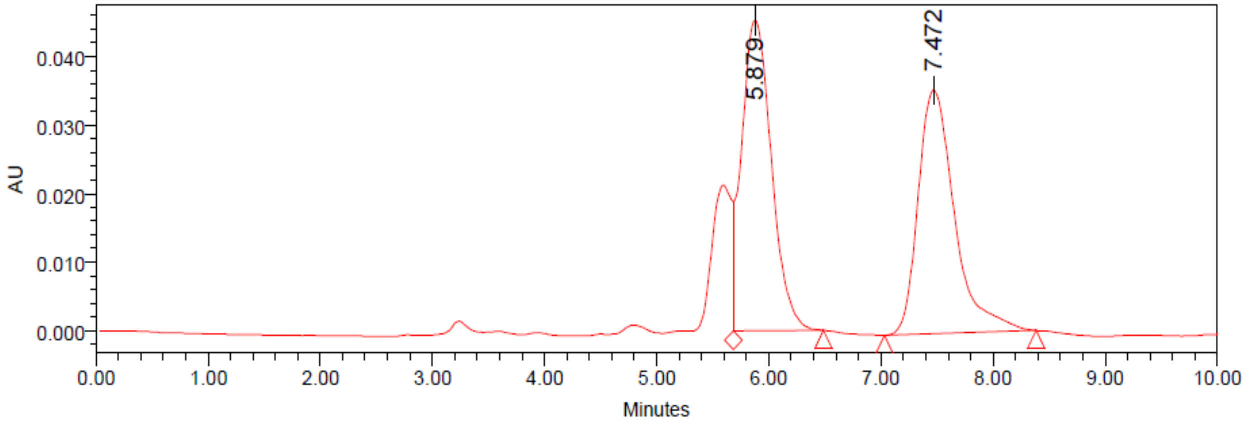

Processed Channel: PDA 221.5 nm

	Processed Channel	Retention Time (min)	Area	% Area	Height
1	PDA 221.5 nm	5.879	839971	51.07	45327
2	PDA 221.5 nm	7.472	804912	48.93	35631

Enantioriched sample of 3Bg:

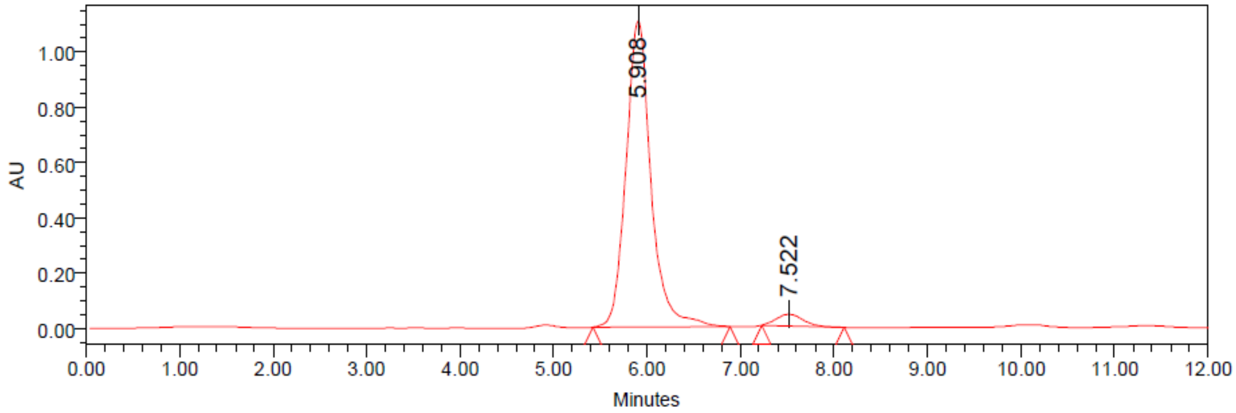

Processed Channel: PDA 221.5 nm

	Processed Channel	Retention Time (min)	Area	% Area	Height
1	PDA 221.5 nm	5.908	20471335	95.98	1107931
2	PDA 221.5 nm	7.522	856459	4.02	42313

^{1}H NMR (400MHz, $CDCl_3$) of **3Bh.**

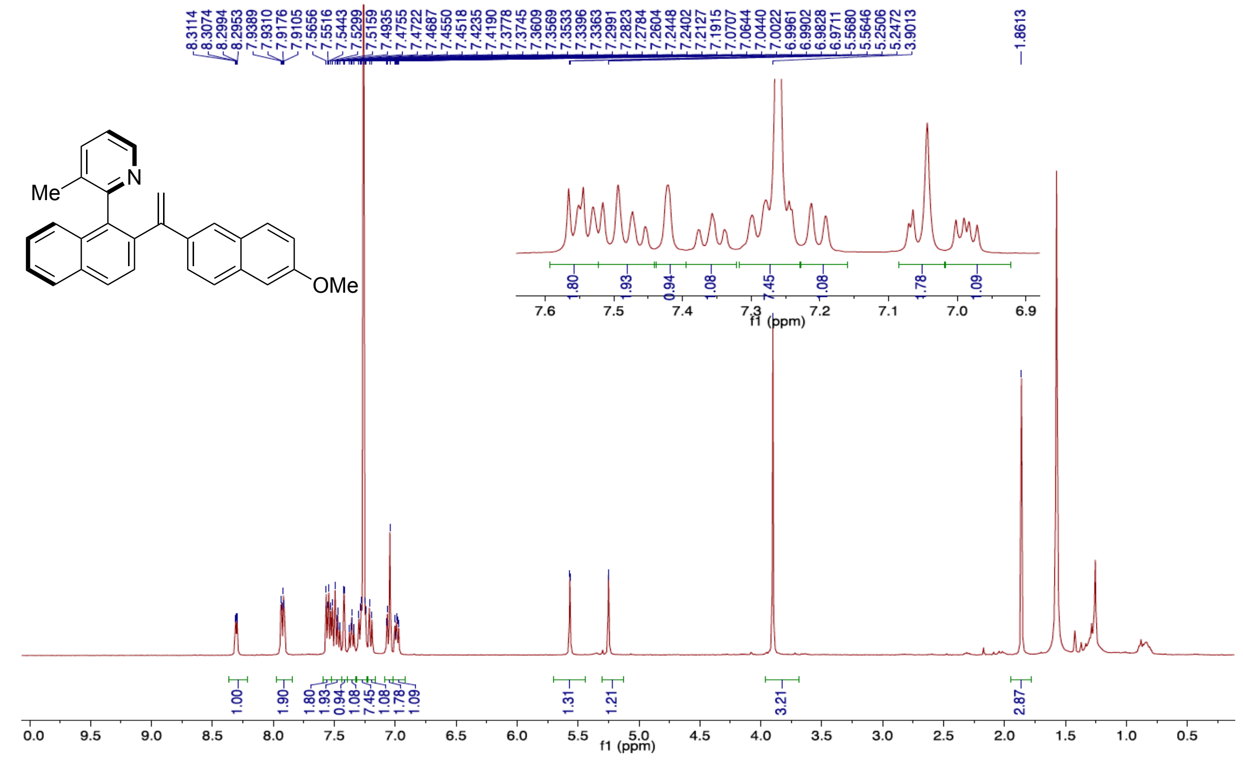

^{13}C NMR (100MHz, $CDCl_3$) of **3Bh.**

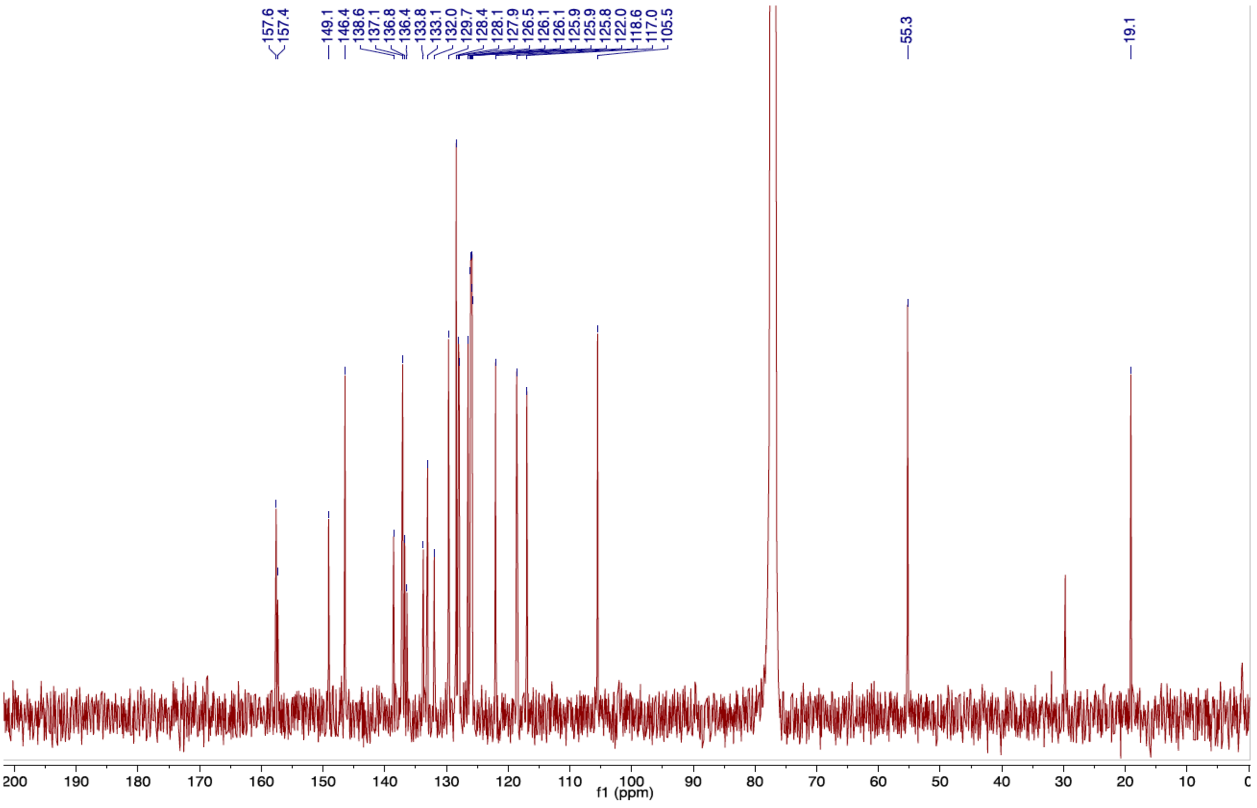

Racemic sample of 3Bh: IA column, Hex:Isop 90:10, T= 30°C, F= 1.0 mL/min.

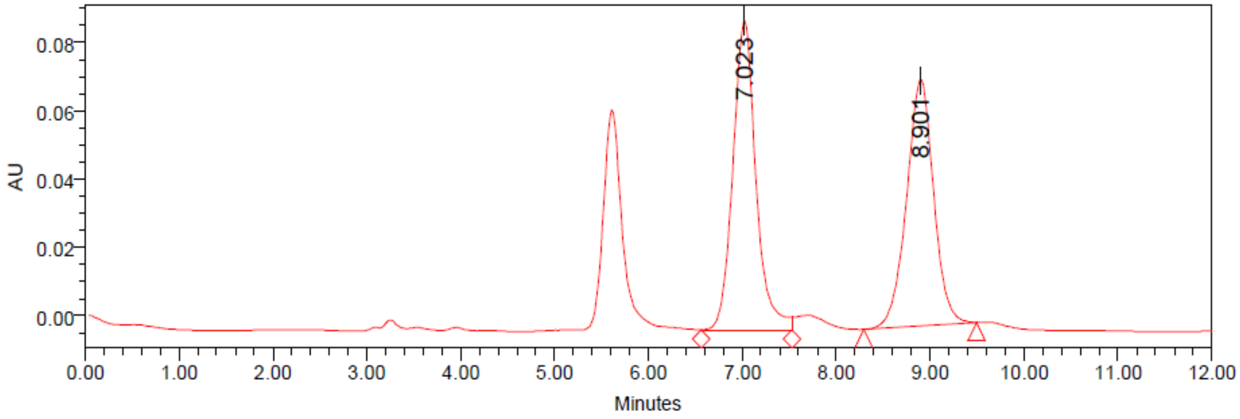

Processed Channel: PDA 221.5 nm

	Processed Channel	Retention Time (min)	Area	% Area	Height
1	PDA 221.5 nm	7.023	1542094	51.58	90739
2	PDA 221.5 nm	8.901	1447497	48.42	72158

Enantioriched sample of 3Bh:

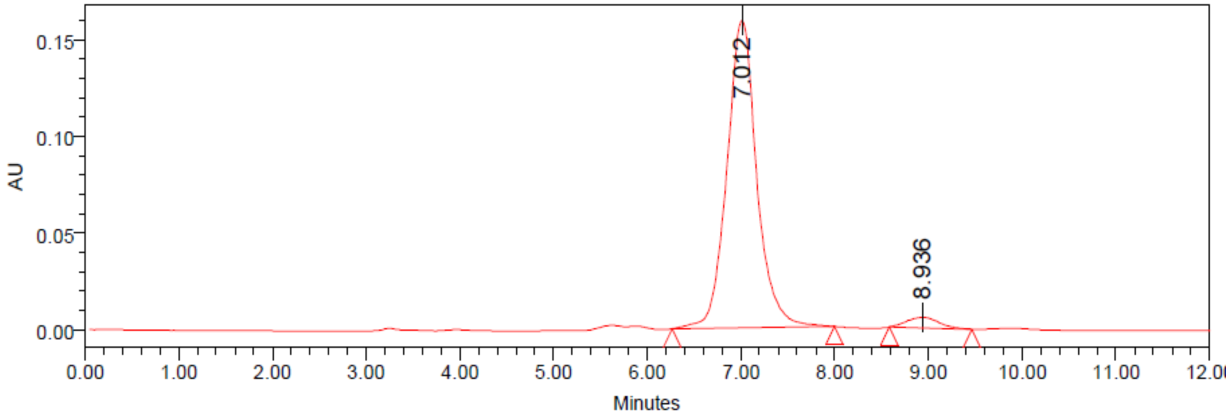

Processed Channel: PDA 221.5 nm

	Processed Channel	Retention Time (min)	Area	% Area	Height
1	PDA 221.5 nm	7.012	3544317	96.45	159261
2	PDA 221.5 nm	8.936	130384	3.55	5381

^1H NMR (400MHz, $CDCl_3$) of **3Ca.**

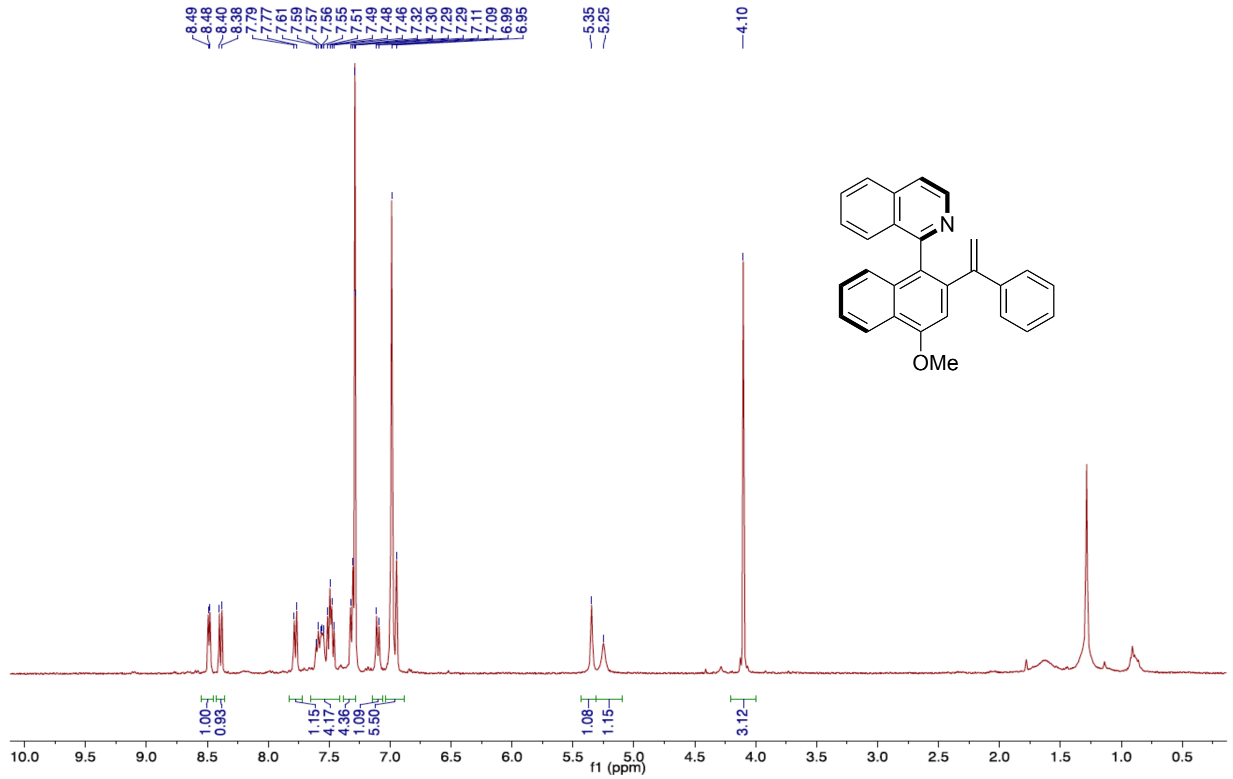

^{13}C NMR (100MHz, $CDCl_3$) of **3Ca.**

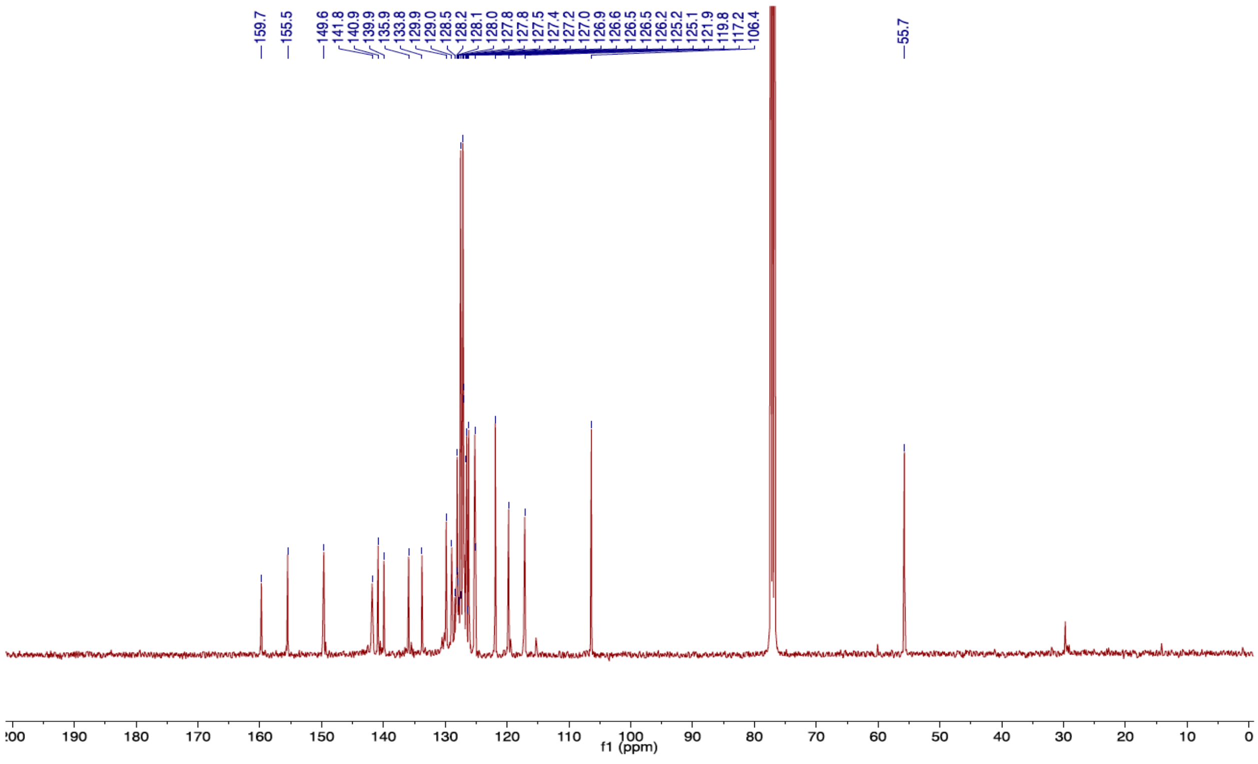

Racemic sample of 3Ca: IA column, Hex:Isop 90:10, T= 30°C, F= 1.0 mL/min.

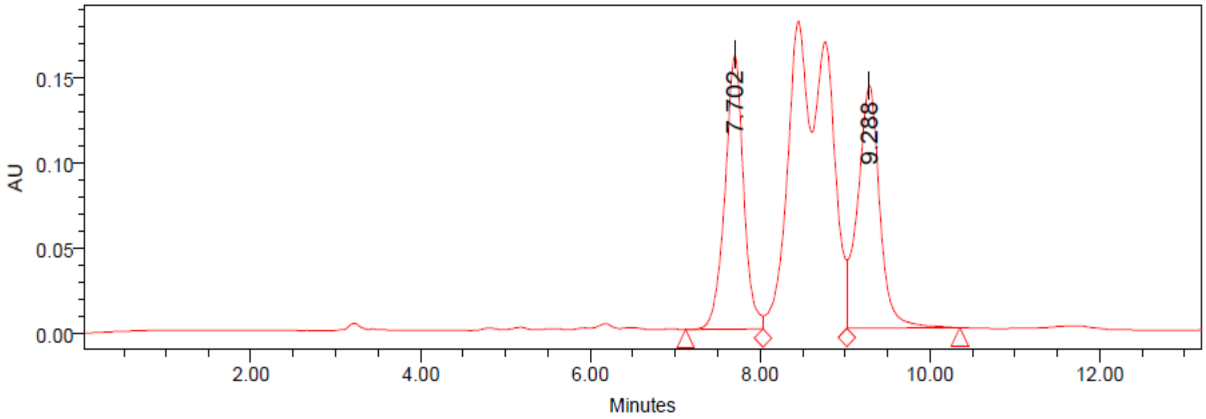

Processed Channel: PDA 221.5 nm

	Processed Channel	Retention Time (min)	Area	% Area	Height
1	PDA 221.5 nm	7.702	2457008	48.58	161363
2	PDA 221.5 nm	9.288	2600968	51.42	142502

Enantioriched sample of 3Ca:

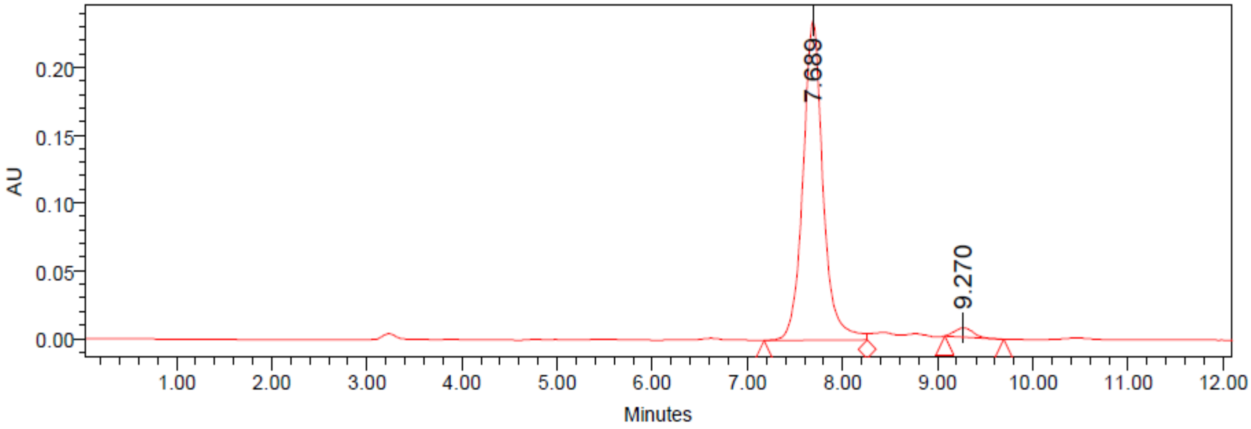

Processed Channel: PDA 221.5 nm

	Processed Channel	Retention Time (min)	Area	% Area	Height
1	PDA 221.5 nm	7.689	3458400	97.50	235891
2	PDA 221.5 nm	9.270	88786	2.50	6670

^{1}H NMR (400MHz, $CDCl_3$) of **3Cg.**

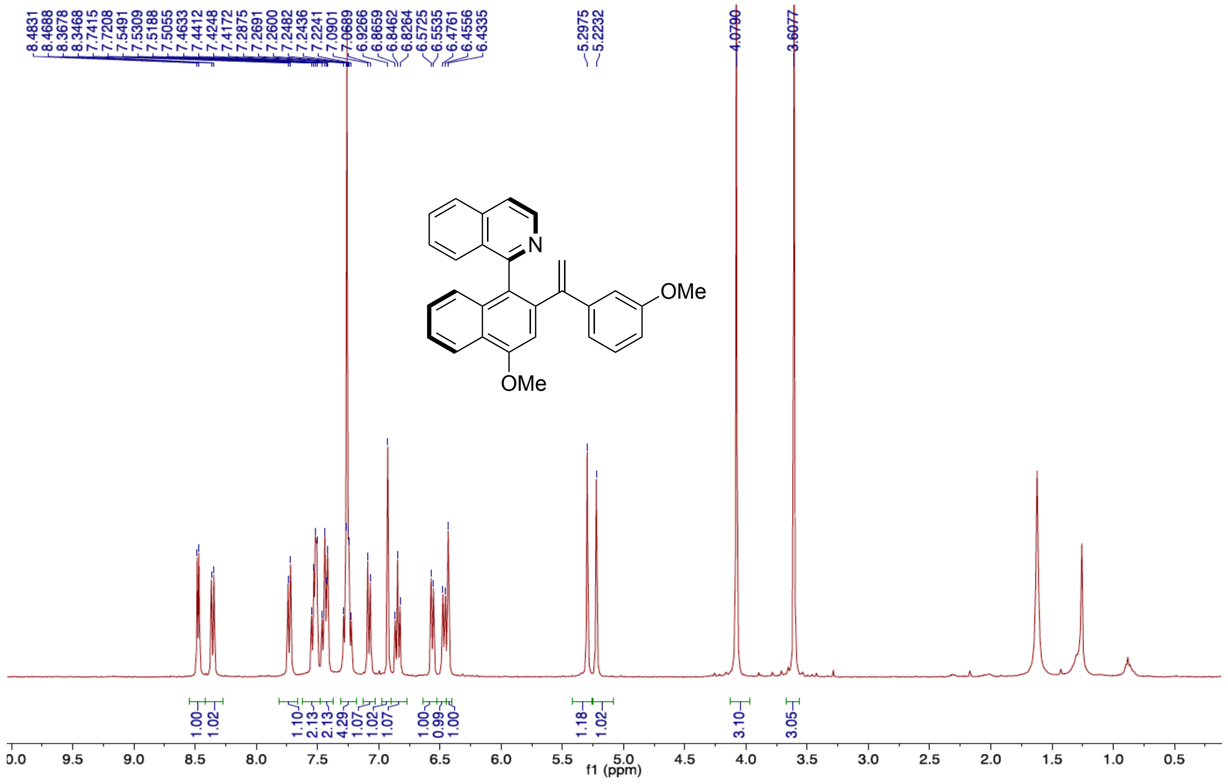

^{13}C NMR (100MHz, $CDCl_3$) of **3Cg.**

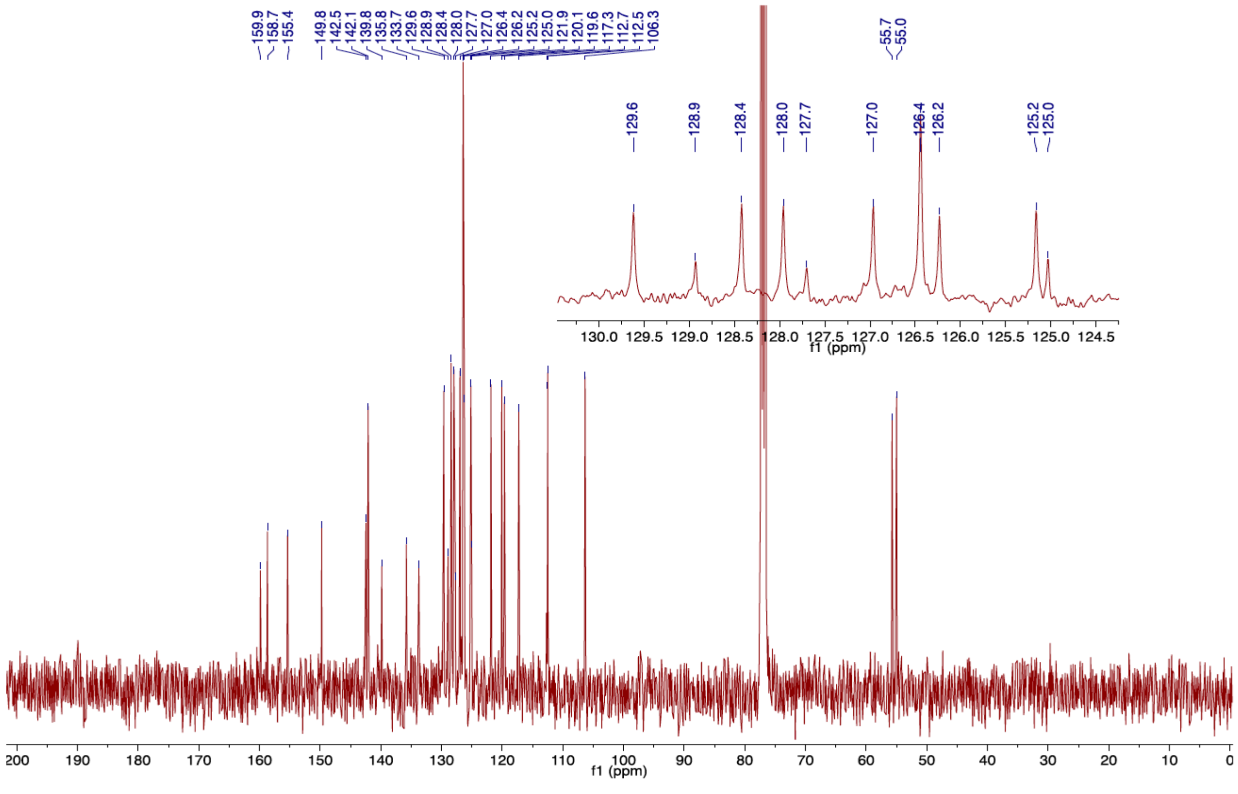

Racemic sample of 3Cg: IA column, Hex:Isop 95:5, T= 30°C, F= 1.0 mL/min.

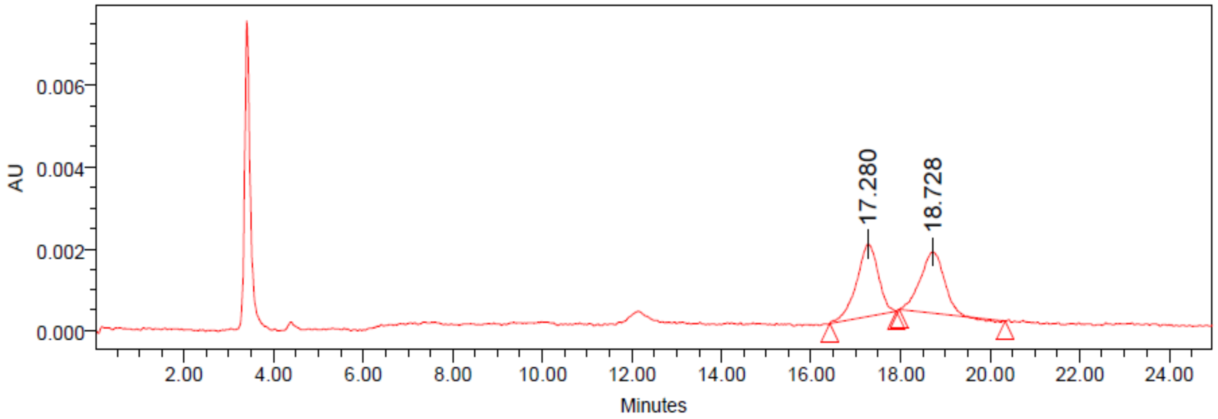

Processed Channel: PDA 271.9 nm

	Processed Channel	Retention Time (min)	Area	% Area	Height
1	PDA 271.9 nm	17.280	59199	50.54	1767
2	PDA 271.9 nm	18.728	57923	49.46	1499

Enantioriched sample of 3Cg:

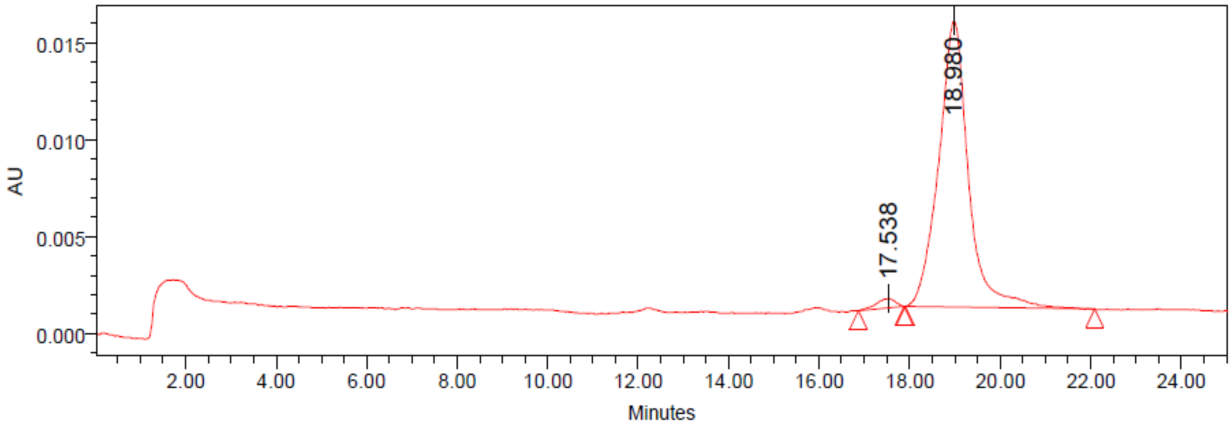

Processed Channel: PDA 271.9 nm

	Processed Channel	Retention Time (min)	Area	% Area	Height
1	PDA 271.9 nm	17.538	14353	2.07	486
2	PDA 271.9 nm	18.980	677603	97.93	14765

^{1}H NMR (400MHz, $CDCl_3$) of **3Ch.**

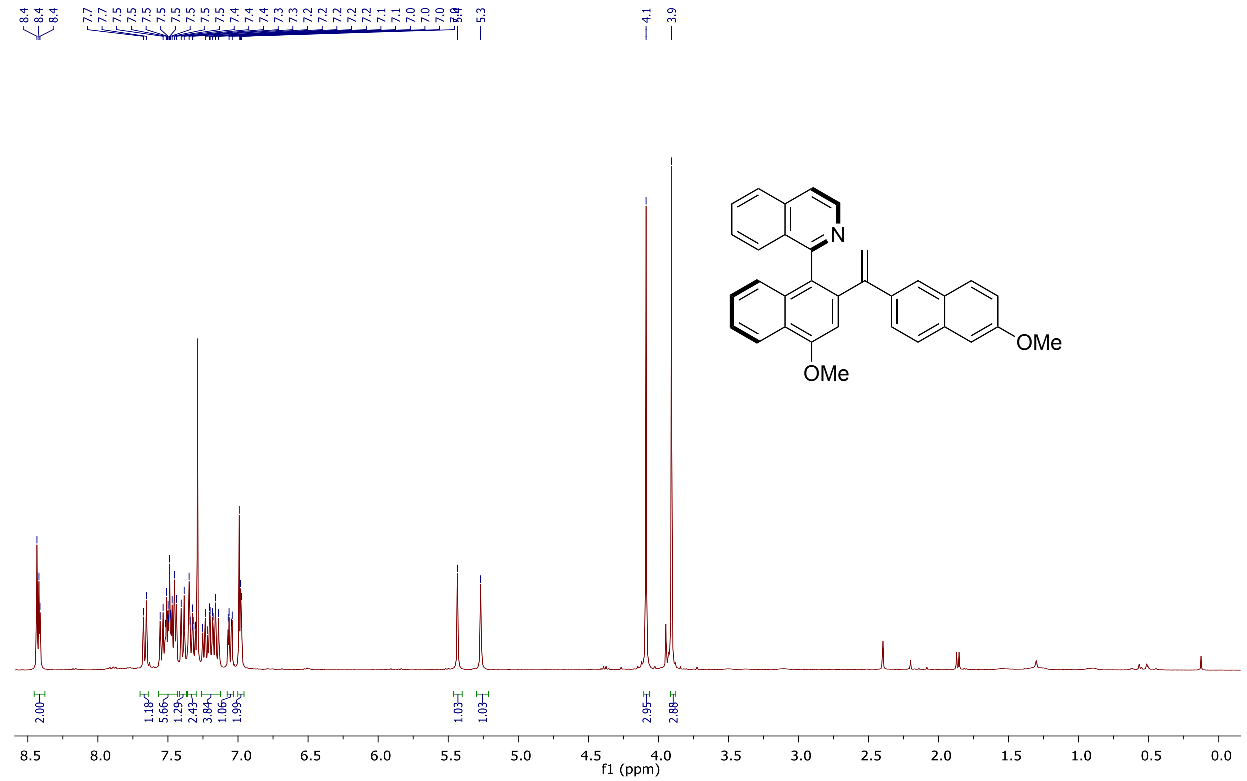

^{13}C NMR (100MHz, $CDCl_3$) of **3Ch.**

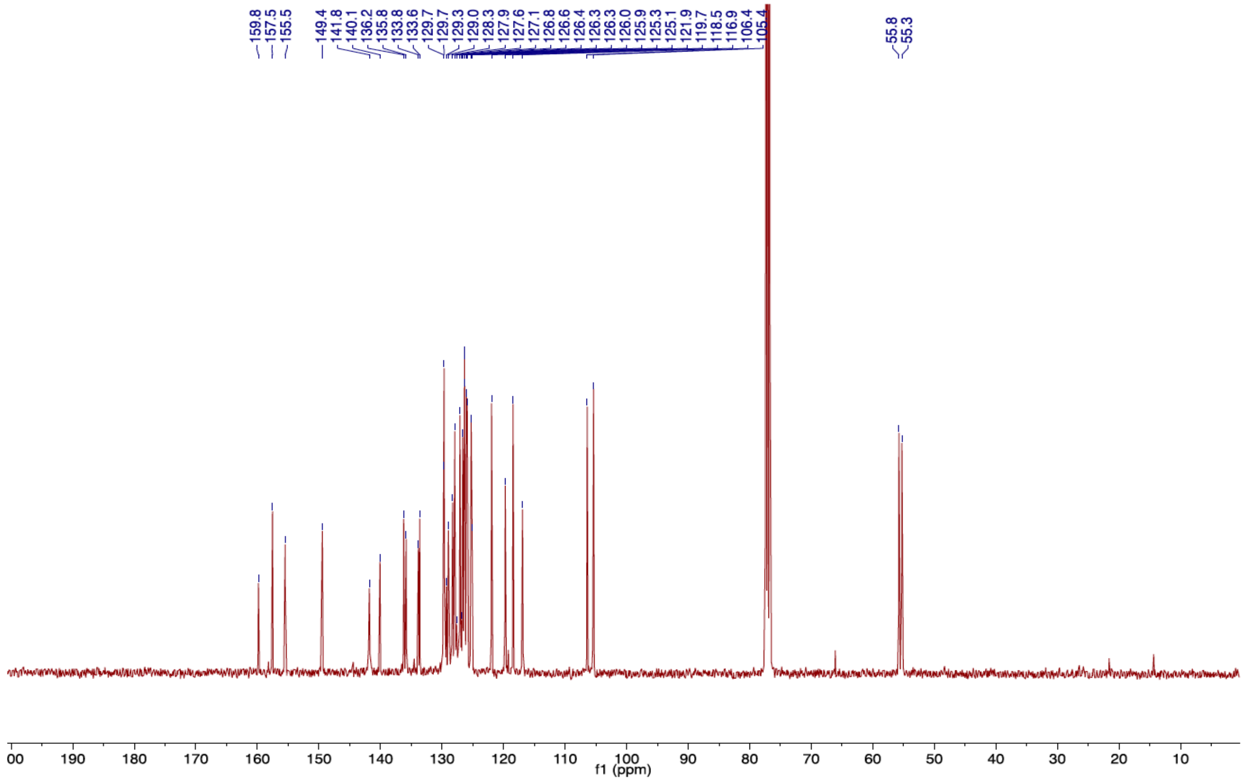

Racemic sample of 3Ch: IA column, Hex:Isop 98:2, T= 30°C, F= 1.0 mL/min.

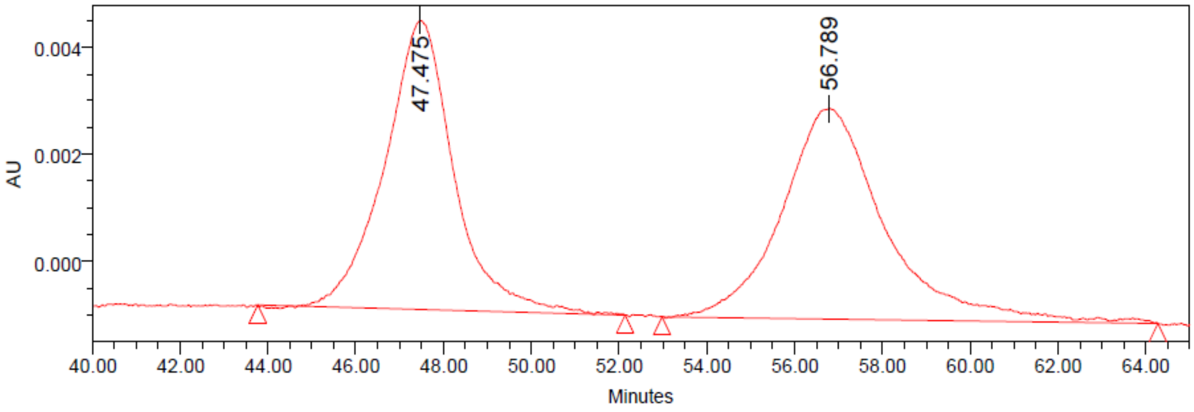

Processed Channel: PDA 217.4 nm

	Processed Channel	Retention Time (min)	Area	% Area	Height
1	PDA 217.4 nm	47.475	594488	48.07	5399
2	PDA 217.4 nm	56.789	642289	51.93	3935

Enantioriched sample of 3Ch:

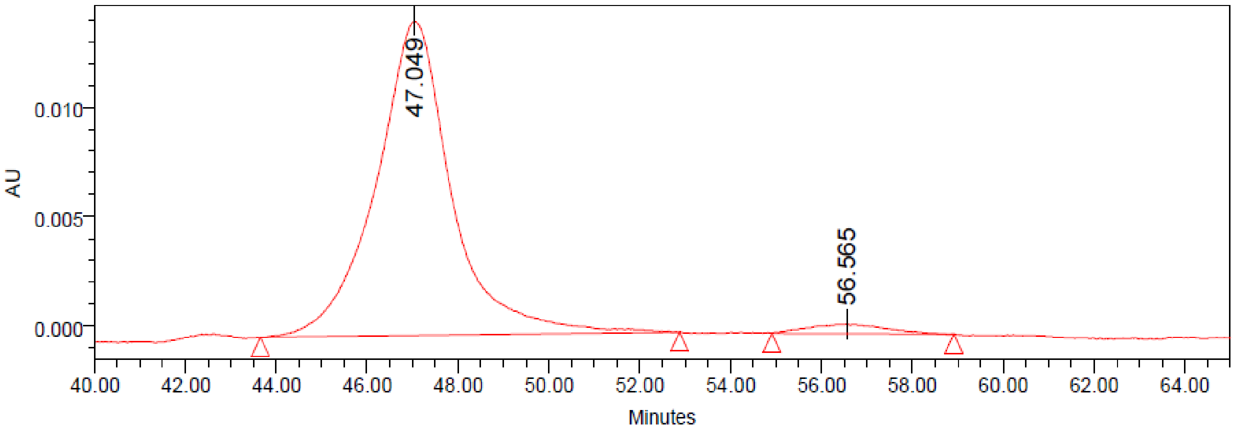

Processed Channel: PDA 217.4 nm

	Processed Channel	Retention Time (min)	Area	% Area	Height
1	PDA 217.4 nm	47.049	1672789	97.08	14390
2	PDA 217.4 nm	56.565	50378	2.92	423

^{1}H NMR (400MHz, $CDCl_3$) of **3Da.**

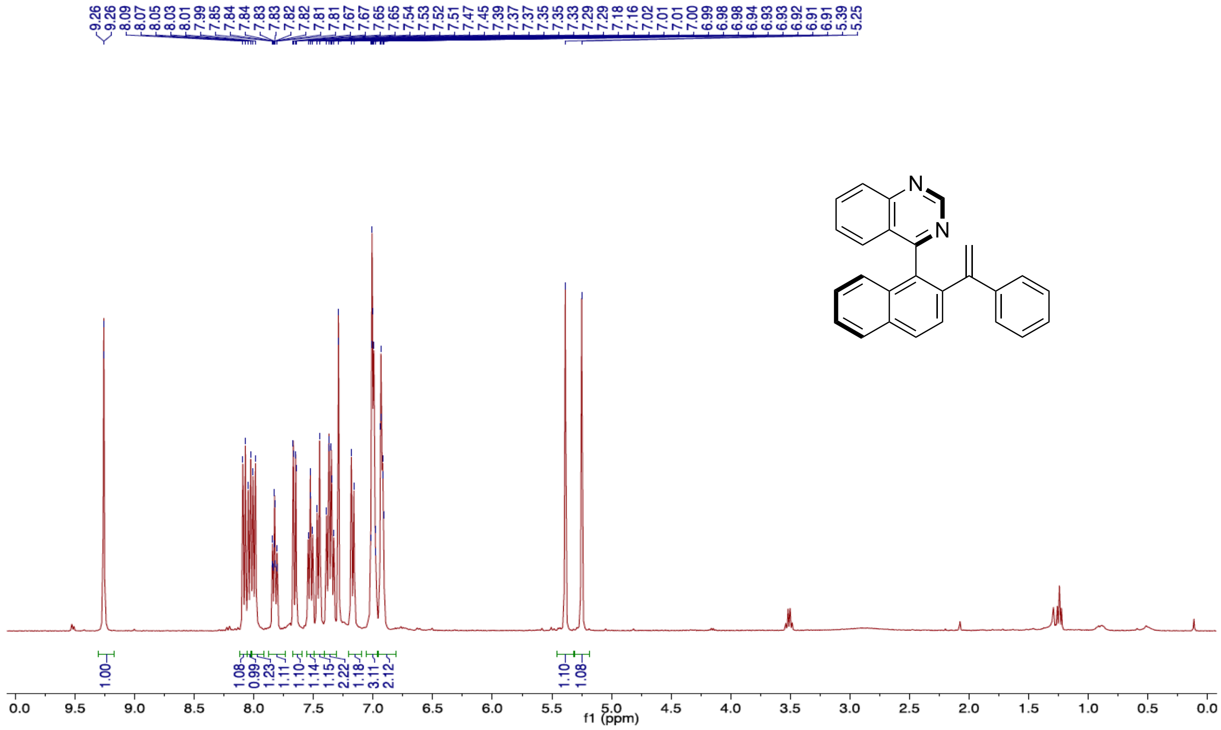

^{13}C NMR (100MHz, $CDCl_3$) of **3Da.**

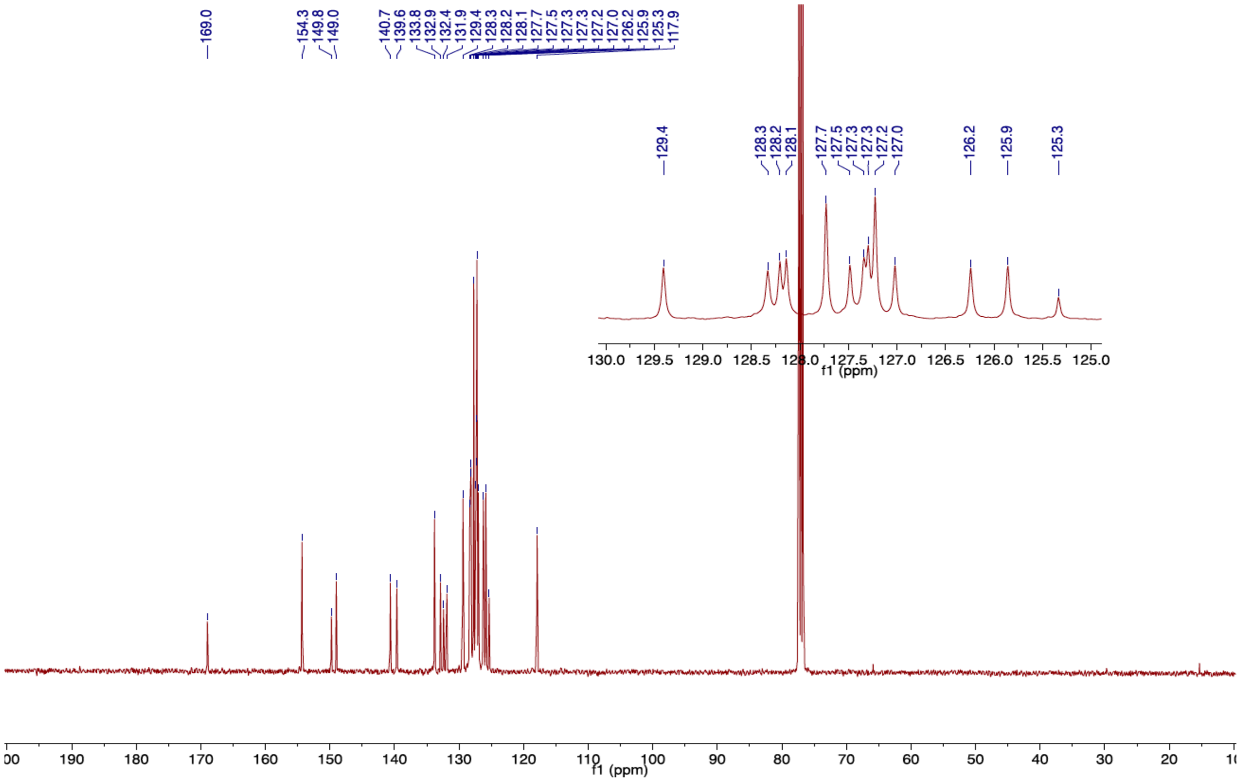

Racemic sample of 3Da: IA column, Hex:Isop 90:10, T= 30°C, F= 1.0 mL/min.

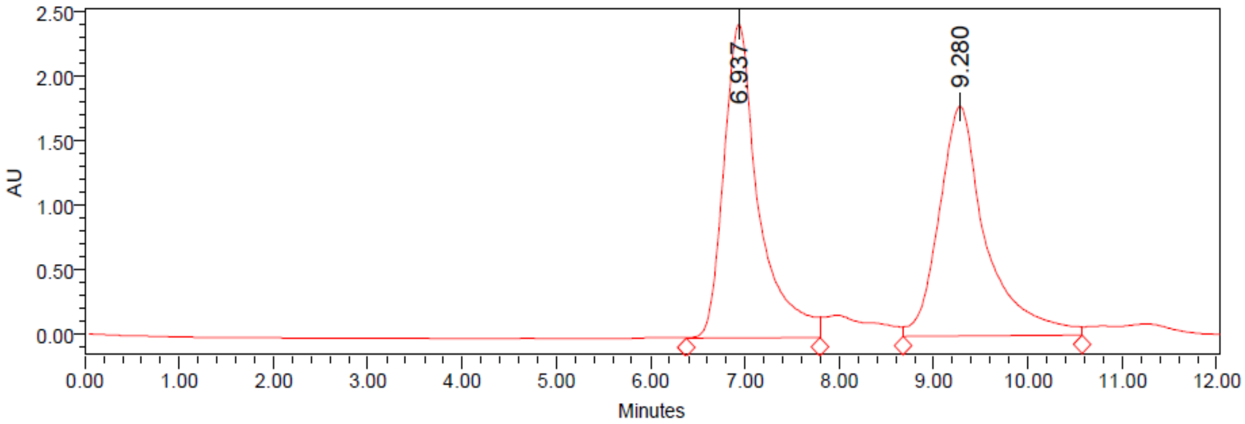

Processed Channel: PDA 221.5 nm

	Processed Channel	Retention Time (min)	Area	% Area	Height
1	PDA 221.5 nm	6.937	62535952	50.22	2433223
2	PDA 221.5 nm	9.280	61978252	49.78	1783329

Enantioriched sample of 3Da:

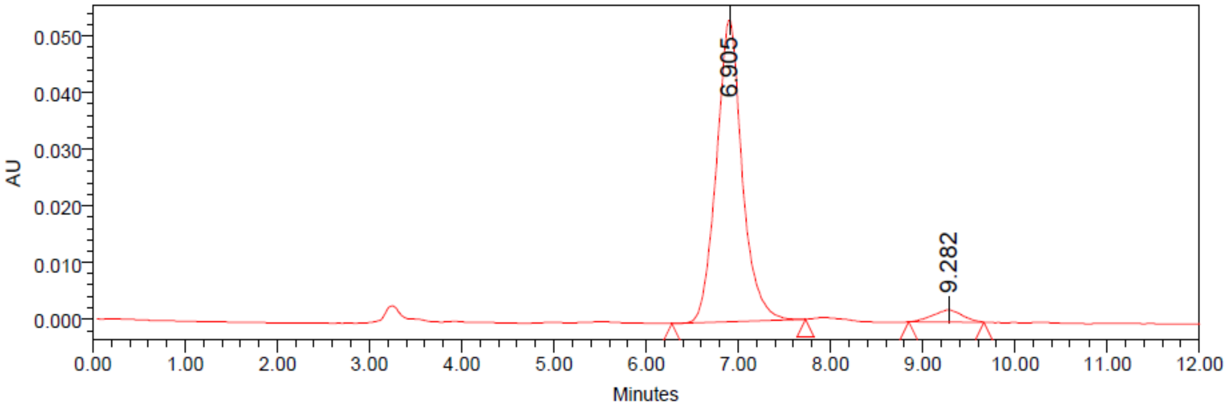

Processed Channel: PDA 221.5 nm

	Processed Channel	Retention Time (min)	Area	% Area	Height
1	PDA 221.5 nm	6.905	1033174	95.75	53322
2	PDA 221.5 nm	9.282	45848	4.25	2080

^{1}H NMR (400MHz, $CDCl_3$) of **3Dc.**

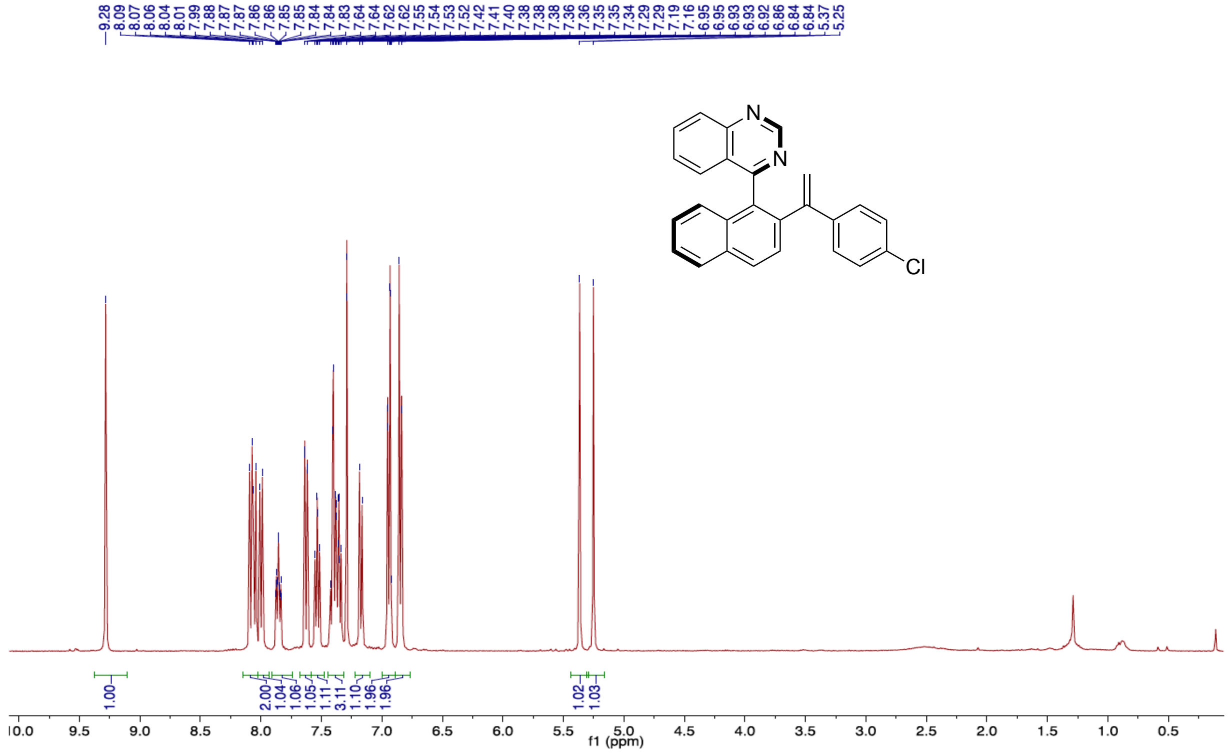

^{13}C NMR (100MHz, $CDCl_3$) of **3Dc.**

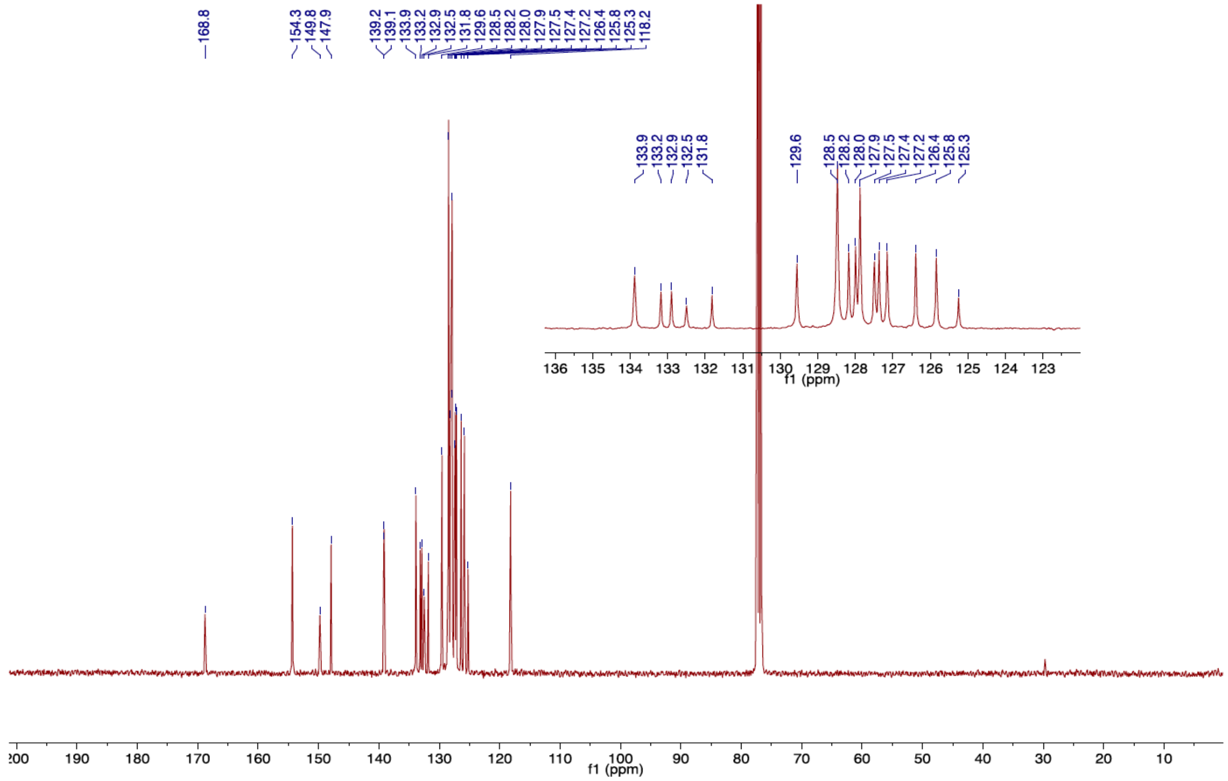

Racemic sample of 3Dc: IA column, Hex:Isop 90:10, T= 30°C, F= 1.0 mL/min.

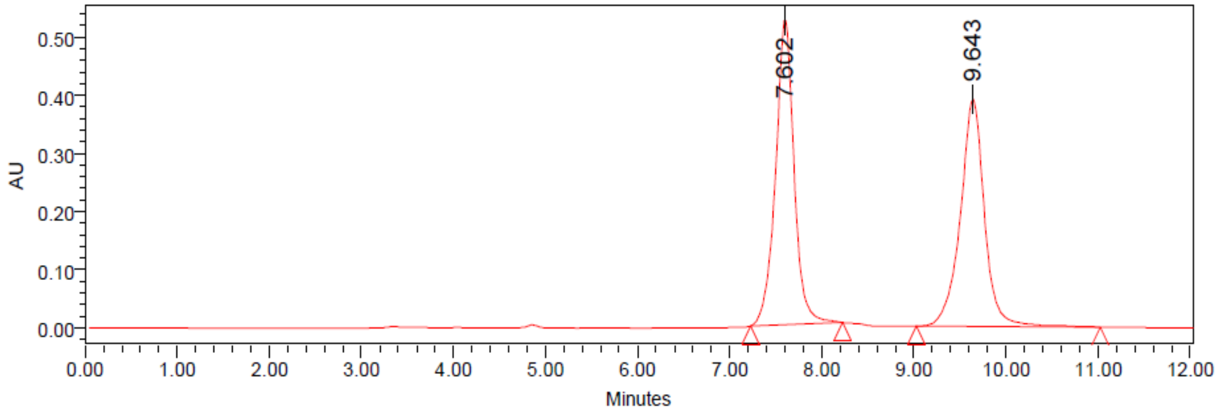

Processed Channel: PDA 221.5 nm

	Processed Channel	Retention Time (min)	Area	% Area	Height
1	PDA 221.5 nm	7.602	7378746	50.88	525975
2	PDA 221.5 nm	9.643	7122550	49.12	391142

Enantioriched sample of 3Dc:

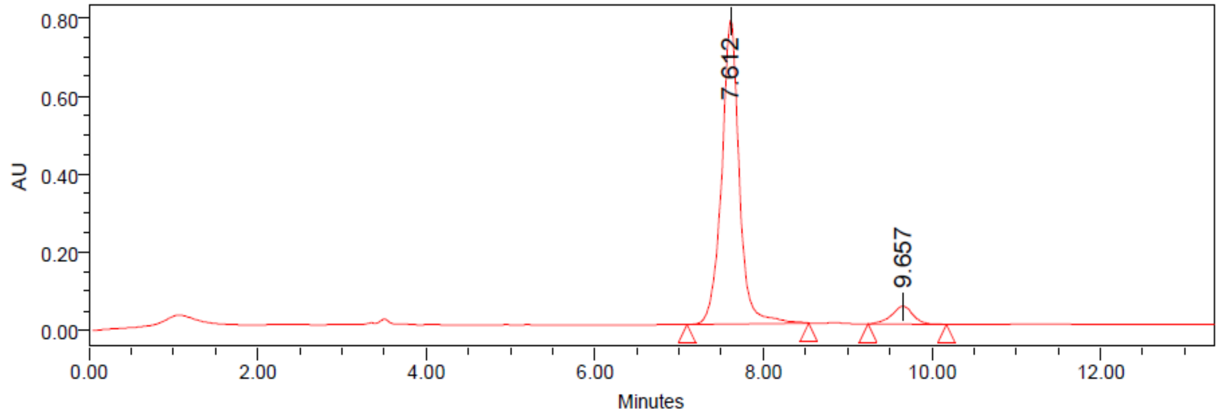

Processed Channel: PDA 209.7 nm

	Processed Channel	Retention Time (min)	Area	% Area	Height
1	PDA 209.7 nm	7.612	11361297	93.48	777893
2	PDA 209.7 nm	9.657	792300	6.52	45506

^{1}H NMR (400MHz, $CDCl_3$) of **3Dd.**

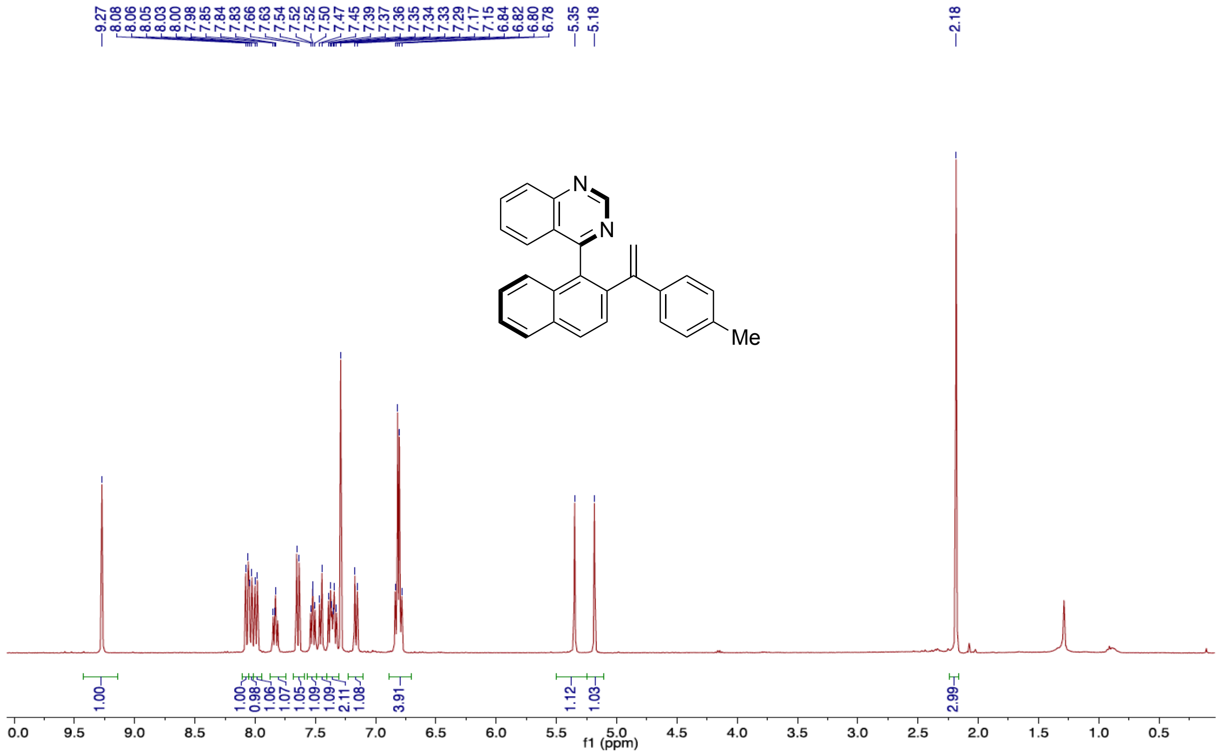

^{13}C NMR (100MHz, $CDCl_3$) of **3Dd.**

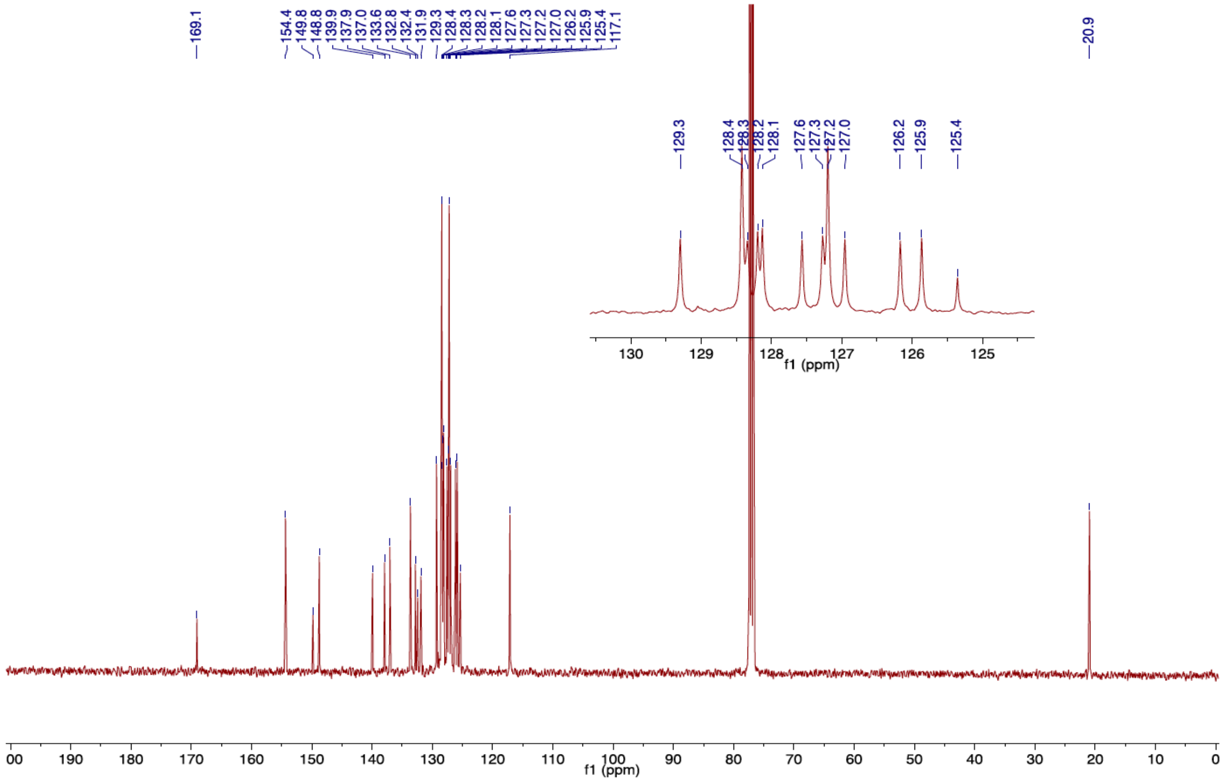

Racemic sample of 3Dd: IA column, Hex:Isop 90:10, T= 30°C, F= 1.0 mL/min.

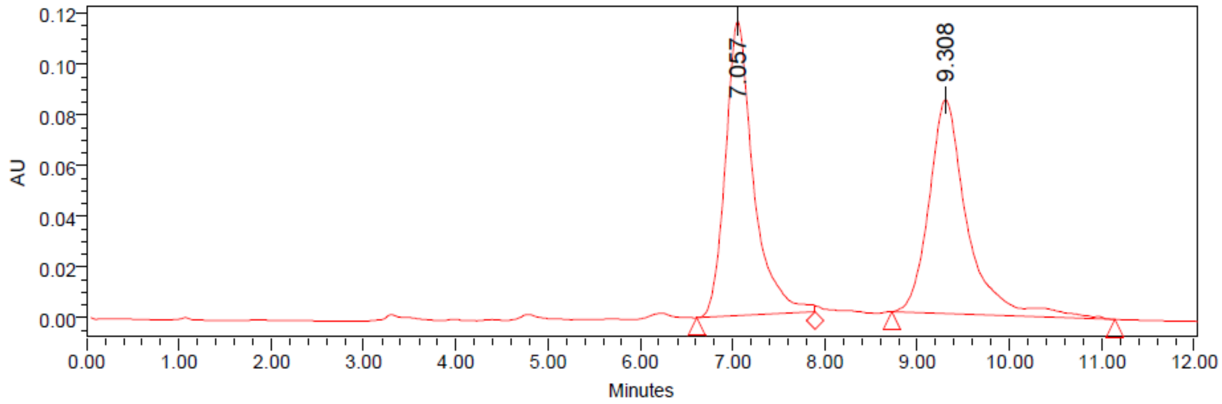

Processed Channel: PDA 221.5 nm

	Processed Channel	Retention Time (min)	Area	% Area	Height
1	PDA 221.5 nm	7.057	2456845	50.20	116131
2	PDA 221.5 nm	9.308	2437731	49.80	84380

Enantioriched sample of 3Dd.

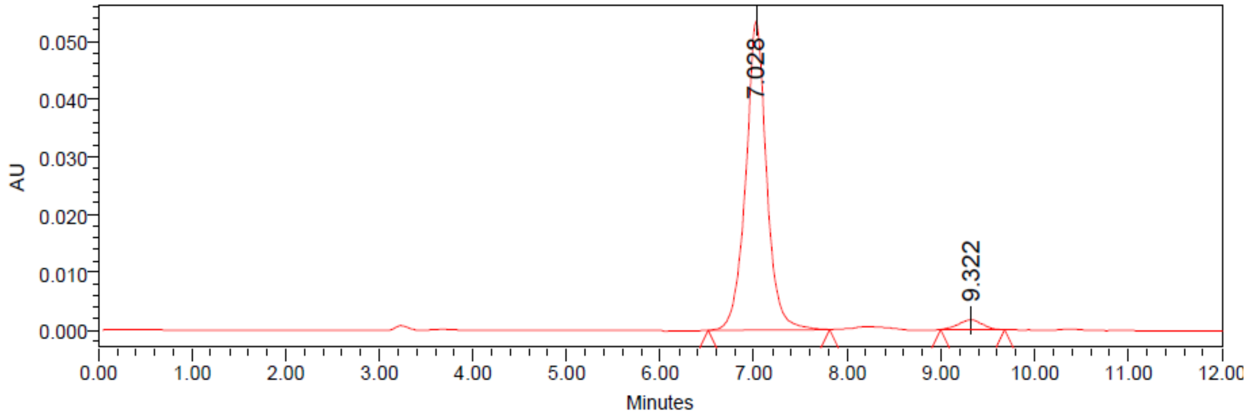

Processed Channel: PDA 255.5 nm

	Processed Channel	Retention Time (min)	Area	% Area	Height
1	PDA 255.5 nm	7.028	829457	96.61	53505
2	PDA 255.5 nm	9.322	29145	3.39	1674

^{1}H NMR (400MHz, $CDCl_3$) of **3De.**

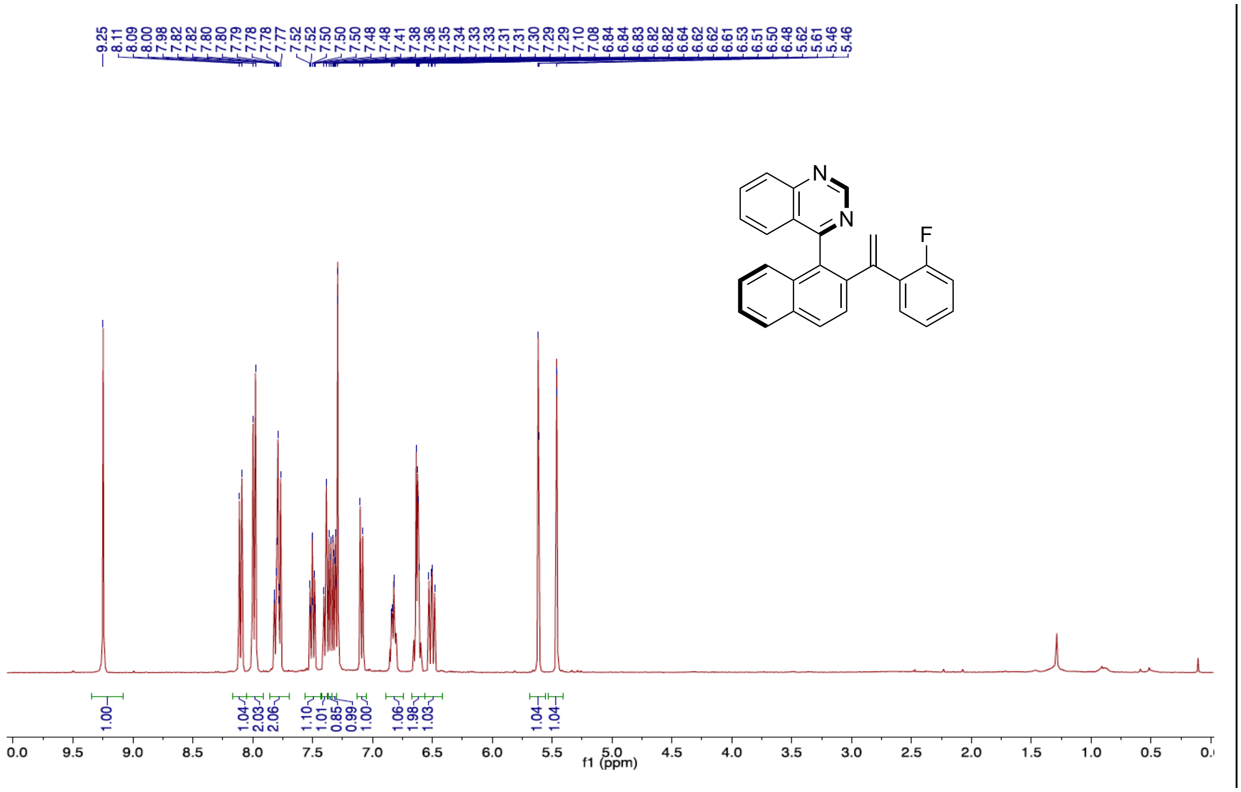

^{13}C NMR (100MHz, $CDCl_3$) of **3De.**

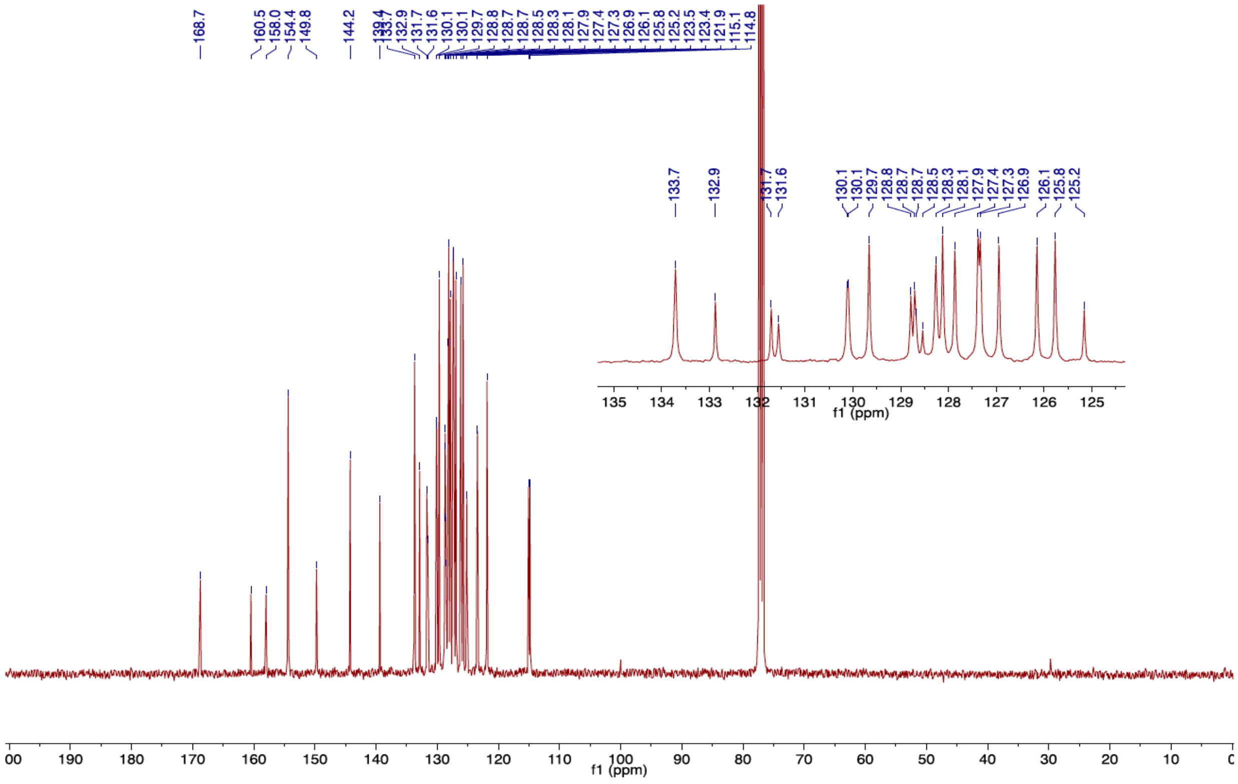

^{19}F NMR (377 MHz, $CDCl_3$) of **3De.**

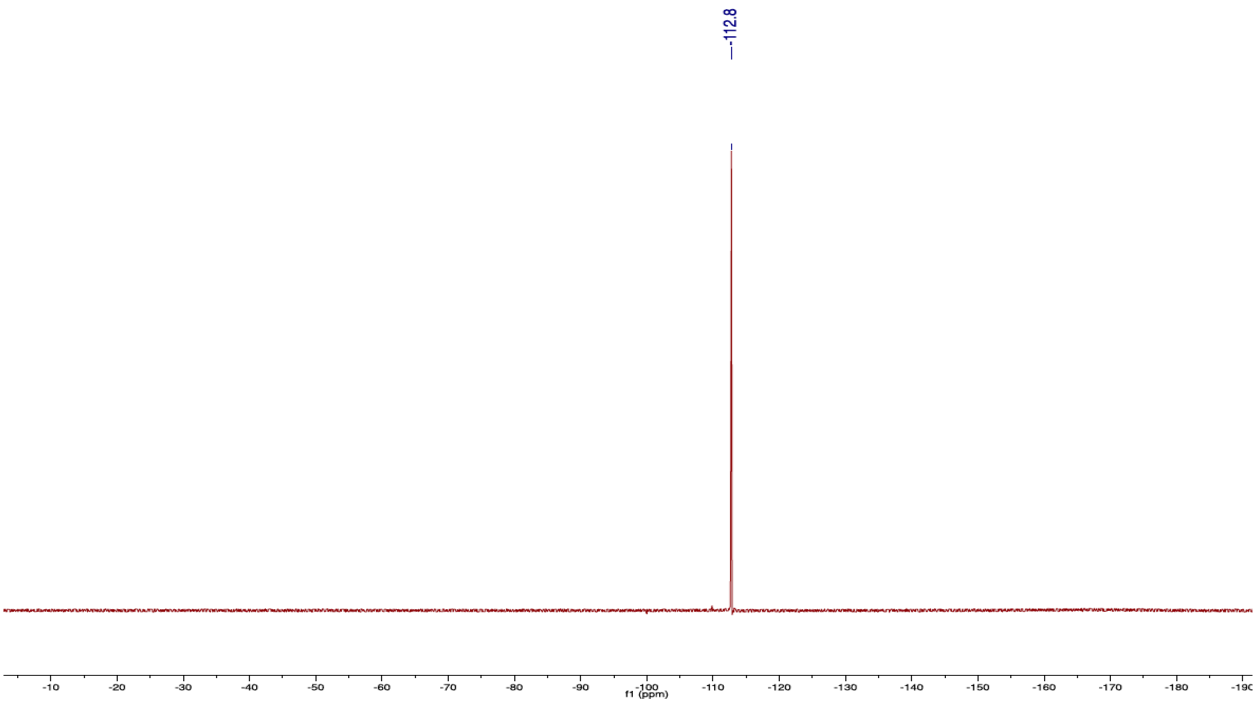

Racemic sample of 3De: IA column, Hex:Isop 90:10, T= 30°C, F= 1.0 mL/min.

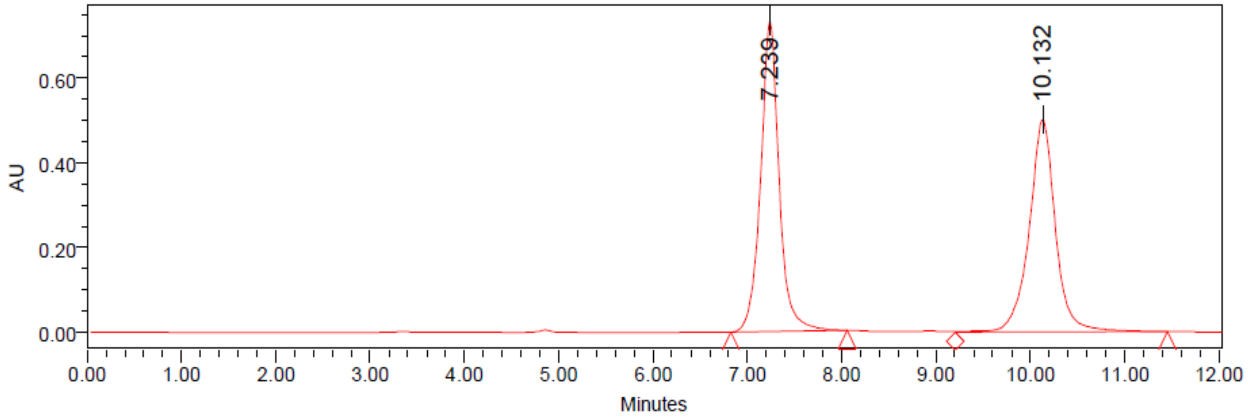

Processed Channel: PDA 221.5 nm

	Processed Channel	Retention Time (min)	Area	% Area	Height
1	PDA 221.5 nm	7.239	10028239	50.72	733907
2	PDA 221.5 nm	10.132	9743292	49.28	501000

Enantioriched sample of 3De:

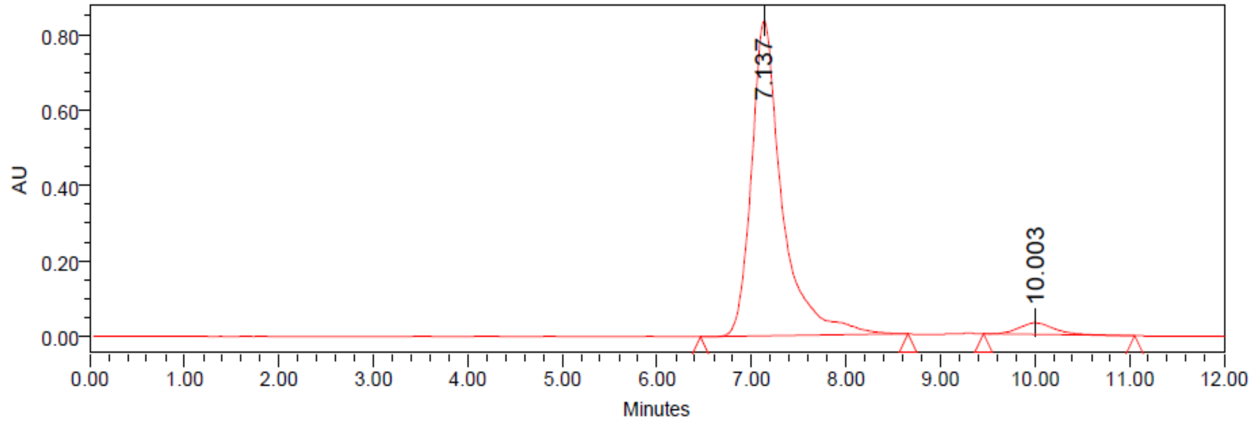

Processed Channel: PDA 248.5 nm

	Processed Channel	Retention Time (min)	Area	% Area	Height
1	PDA 248.5 nm	7.137	18899980	95.78	836550
2	PDA 248.5 nm	10.003	832958	4.22	31060

^{1}H NMR (400MHz, $CDCl_3$) of **3Dg.**

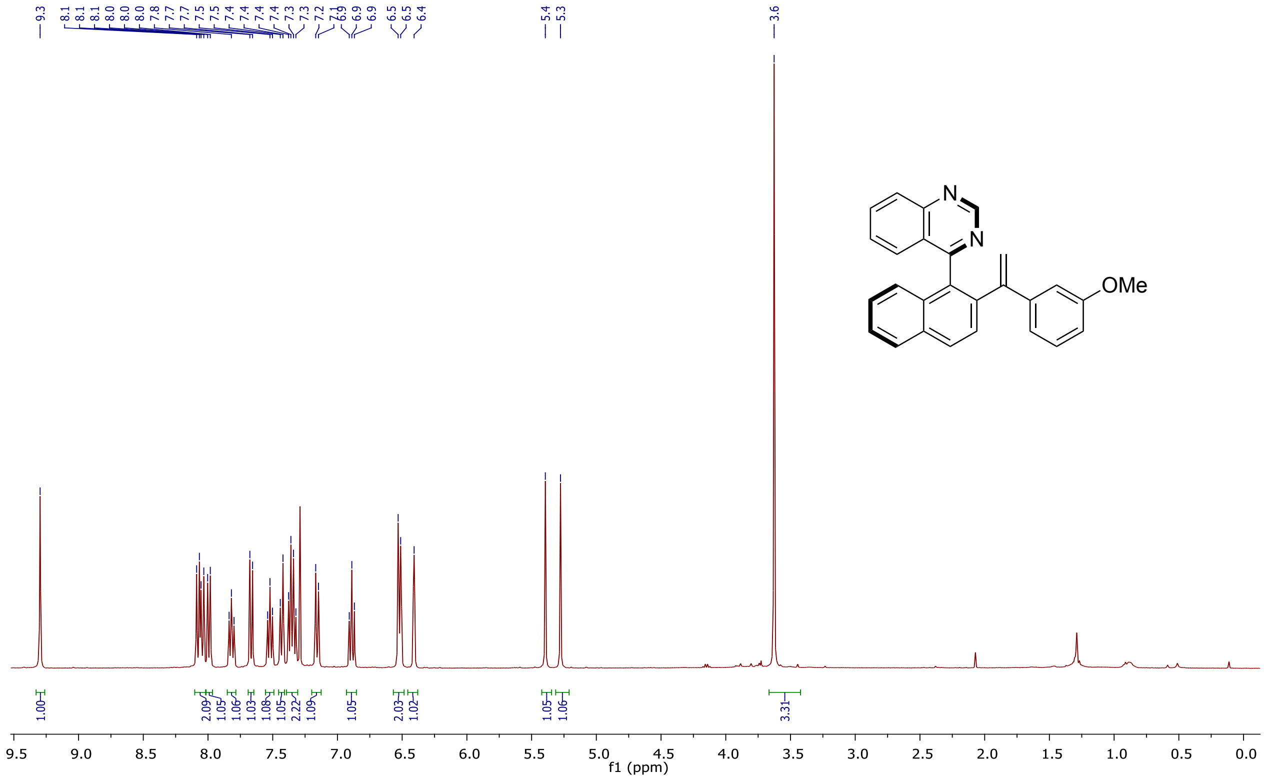

^{13}C NMR (100MHz, $CDCl_3$) of **3Dg.**

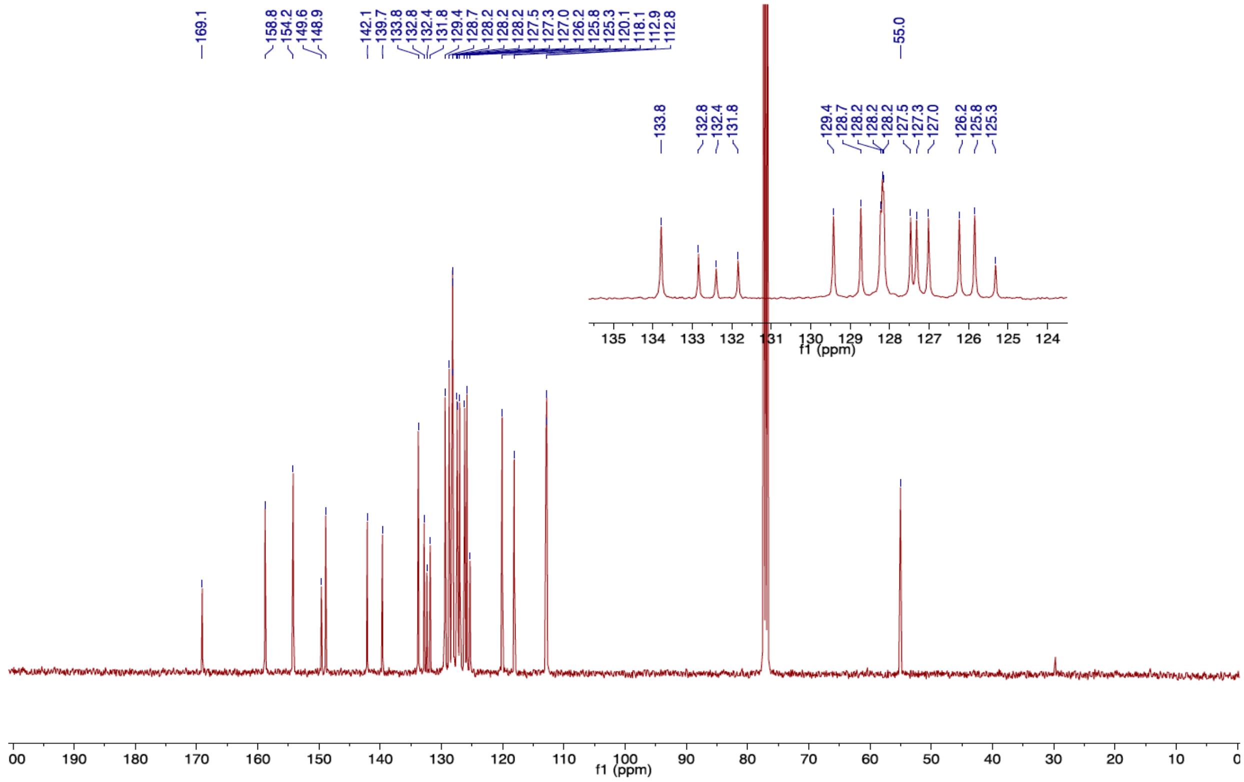

Racemic sample of 3Dg: IA column, Hex:Isop 90:10, T= 30°C, F= 1.0 mL/min.

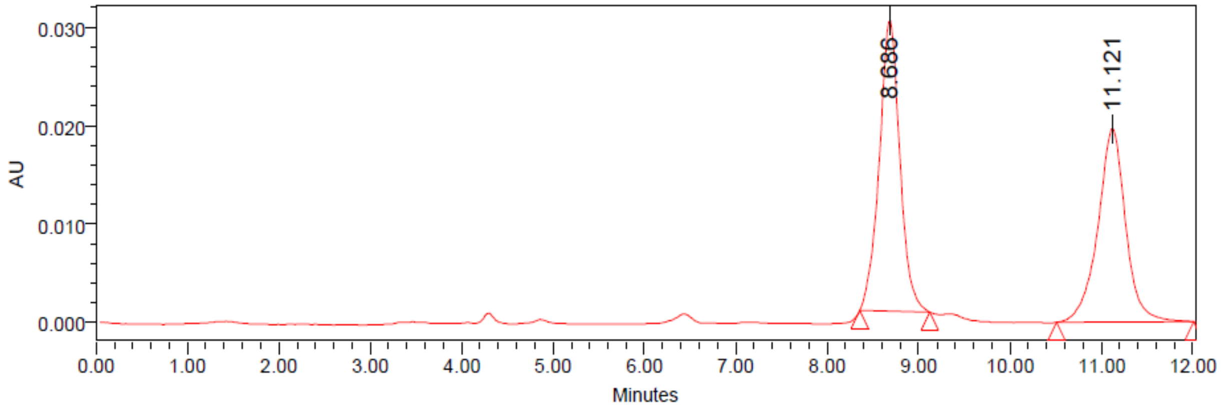

Processed Channel: PDA 260.1 nm

	Processed Channel	Retention Time (min)	Area	% Area	Height
1	PDA 260.1 nm	8.686	463688	52.62	29616
2	PDA 260.1 nm	11.121	417431	47.38	19729

Enantioriched sample of 3Dg.

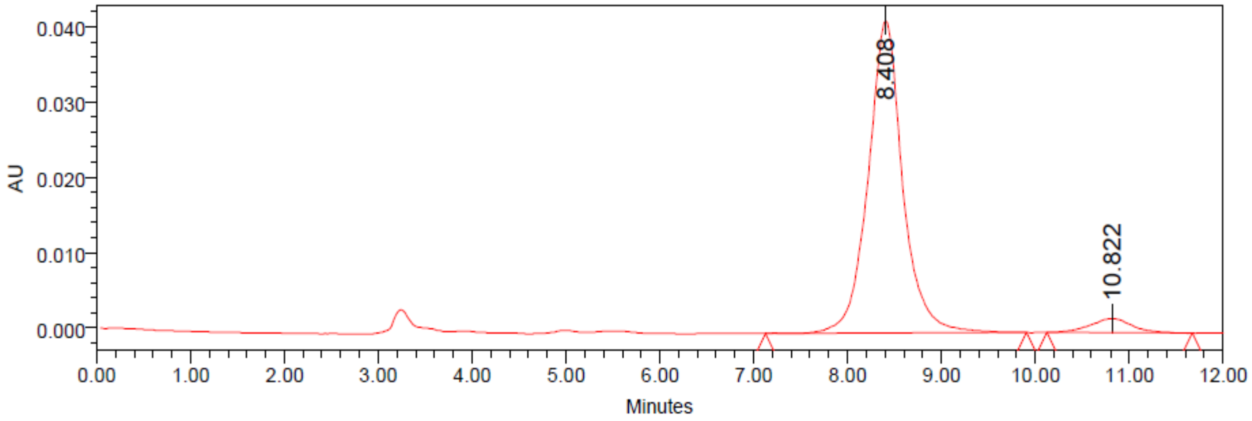

Processed Channel: PDA 221.5 nm

	Processed Channel	Retention Time (min)	Area	% Area	Height
1	PDA 221.5 nm	8.408	1062026	94.85	41424
2	PDA 221.5 nm	10.822	57712	5.15	1876

^{1}H NMR (400MHz, $CDCl_3$) of **3Dh.**

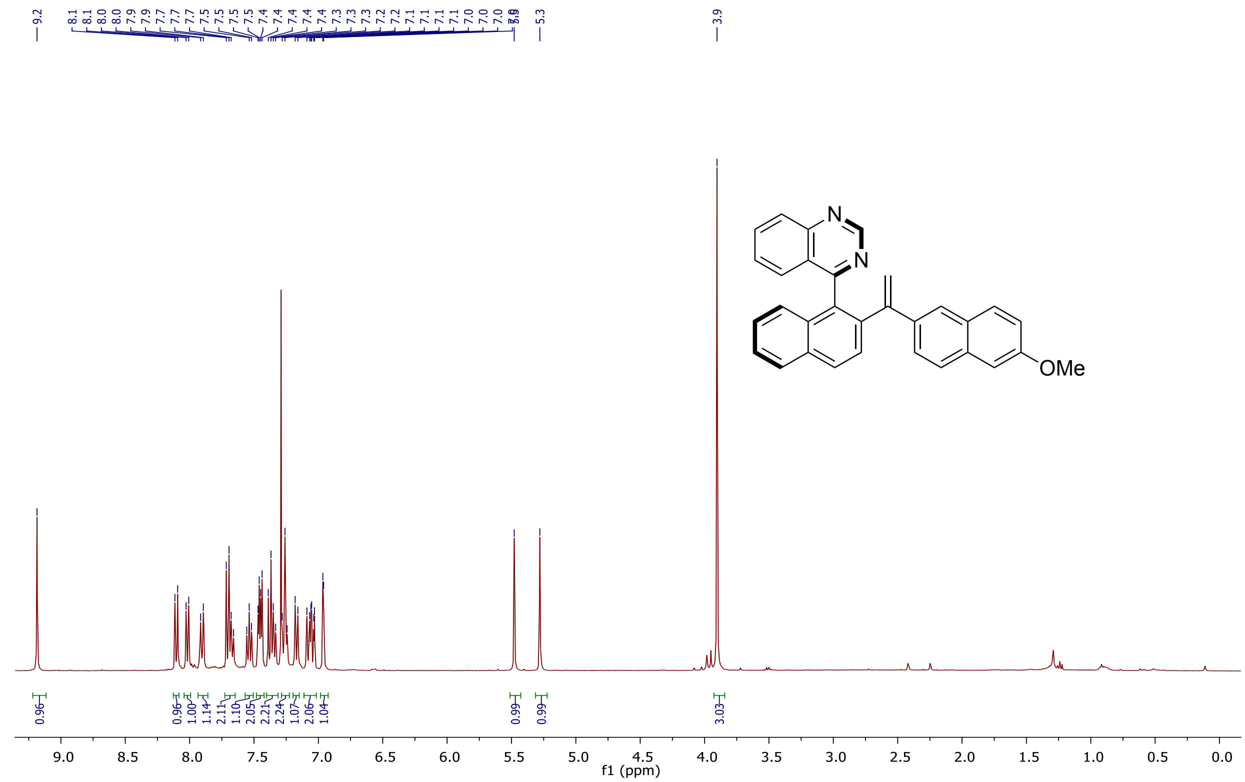

^{13}C NMR (100MHz, $CDCl_3$) of **3Dh.**

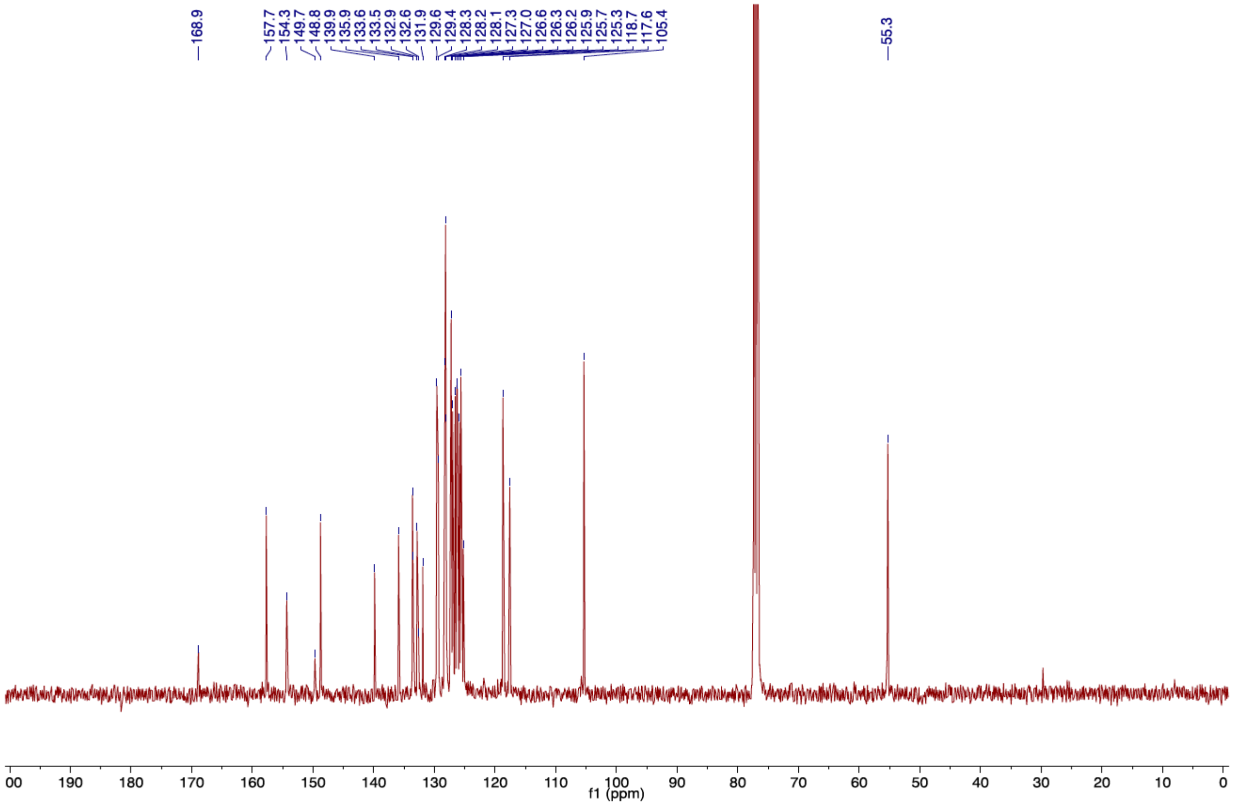

Racemic sample of 3Dh: IA column, Hex:Isop 90:10, T= 30°C, F= 1.0 mL/min.

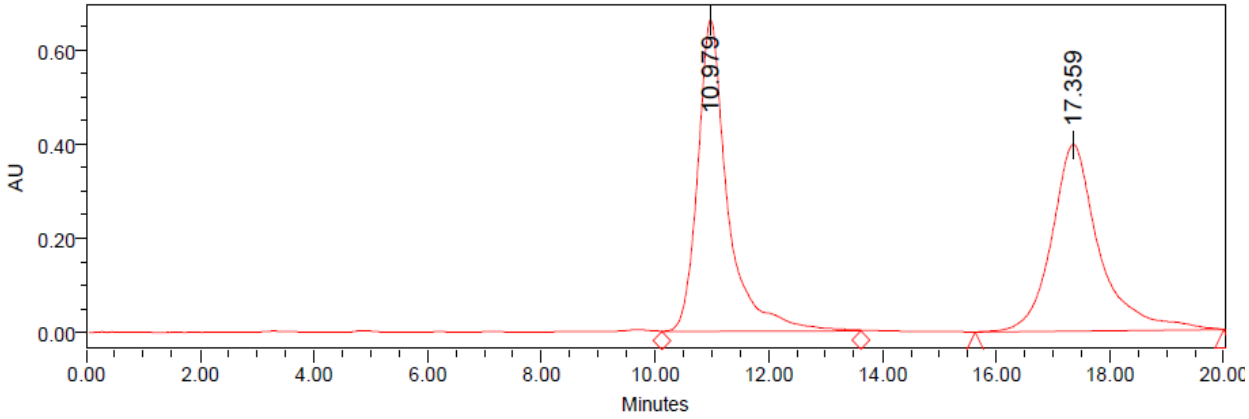

Processed Channel: PDA 221.5 nm

	Processed Channel	Retention Time (min)	Area	% Area	Height
1	PDA 221.5 nm	10.979	24173165	51.20	660164
2	PDA 221.5 nm	17.359	23041808	48.80	396153

Enantioriched sample of 3Dh:

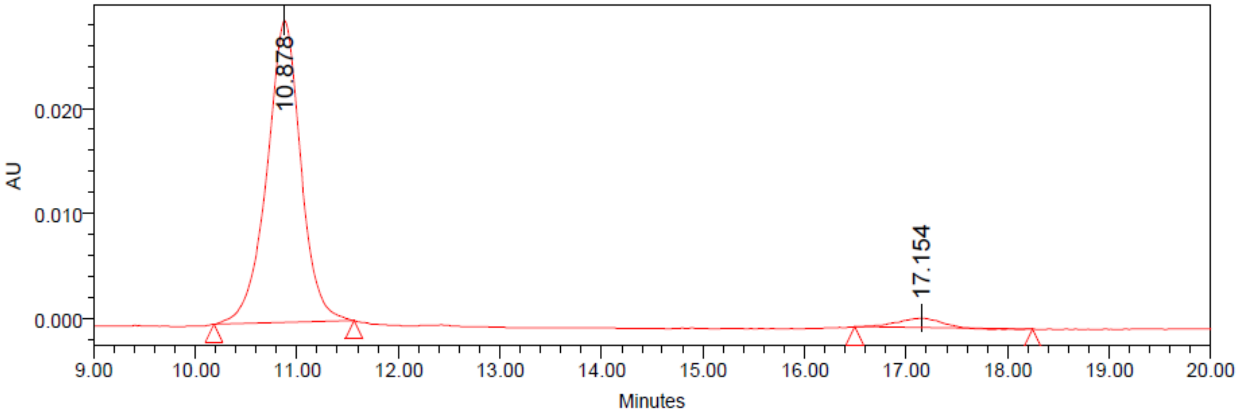

Processed Channel: PDA 221.5 nm

	Processed Channel	Retention Time (min)	Area	% Area	Height
1	PDA 221.5 nm	10.878	670884	95.87	28817
2	PDA 221.5 nm	17.154	28899	4.13	917

^{1}H NMR (400MHz, $CDCl_3$) of *(Rₐ)*-**4Aa.**

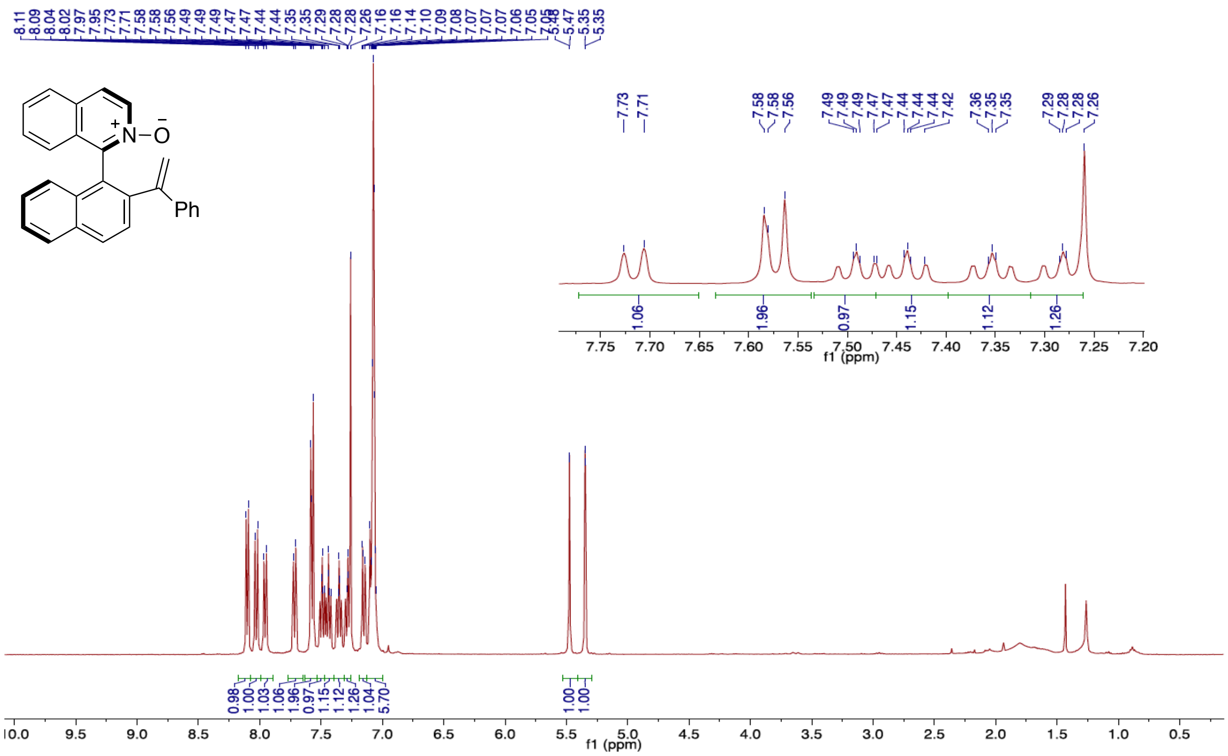

^{13}C NMR (100MHz, $CDCl_3$) of *(Rₐ)*-**4Aa.**

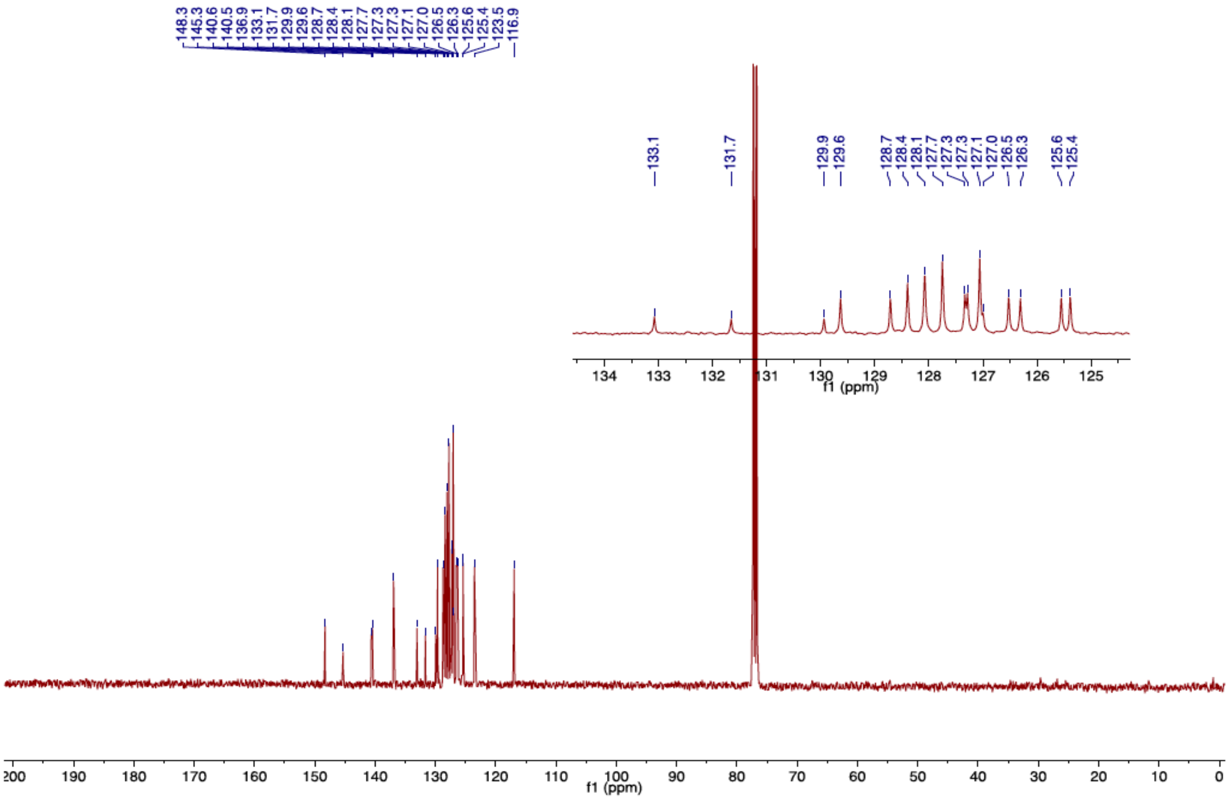

^{1}H NMR (400MHz, $CDCl_3$) of *(R$_a$)*-**5Aa.**

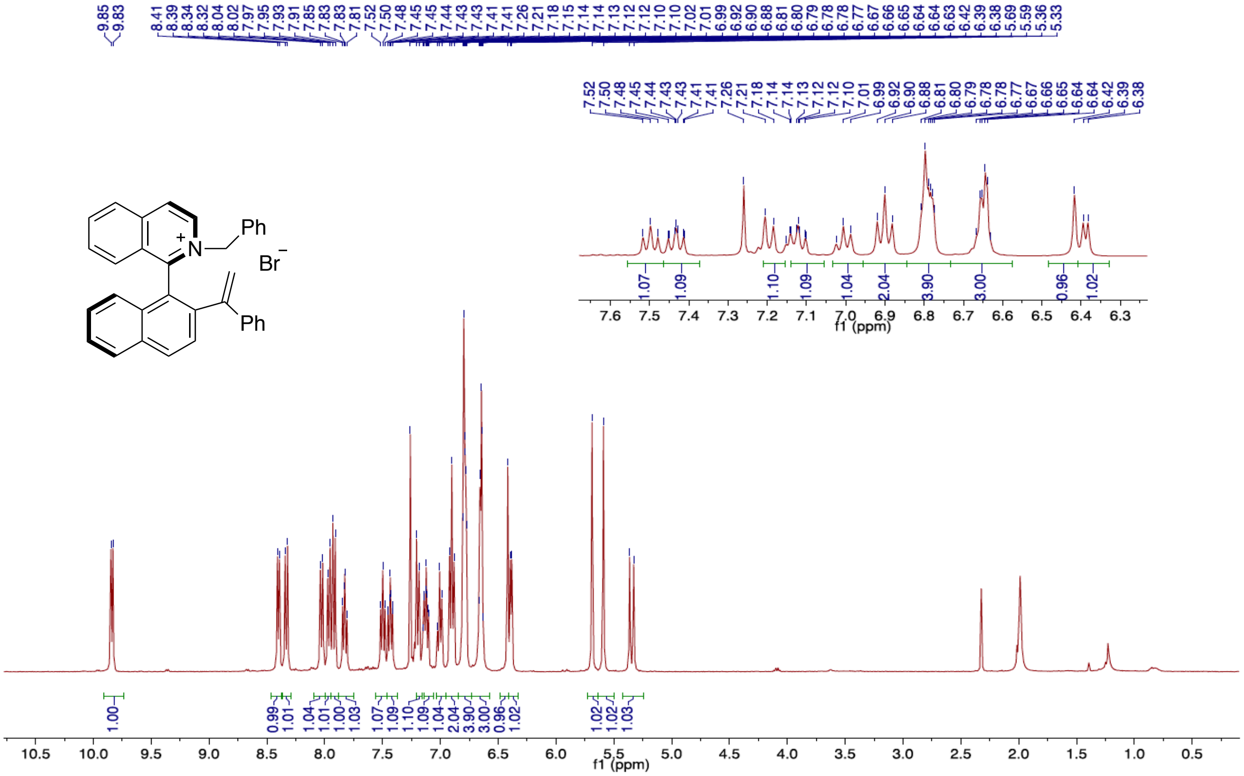

^{13}C NMR (100MHz, $CDCl_3$) of *(R$_a$)*-**5Aa.**

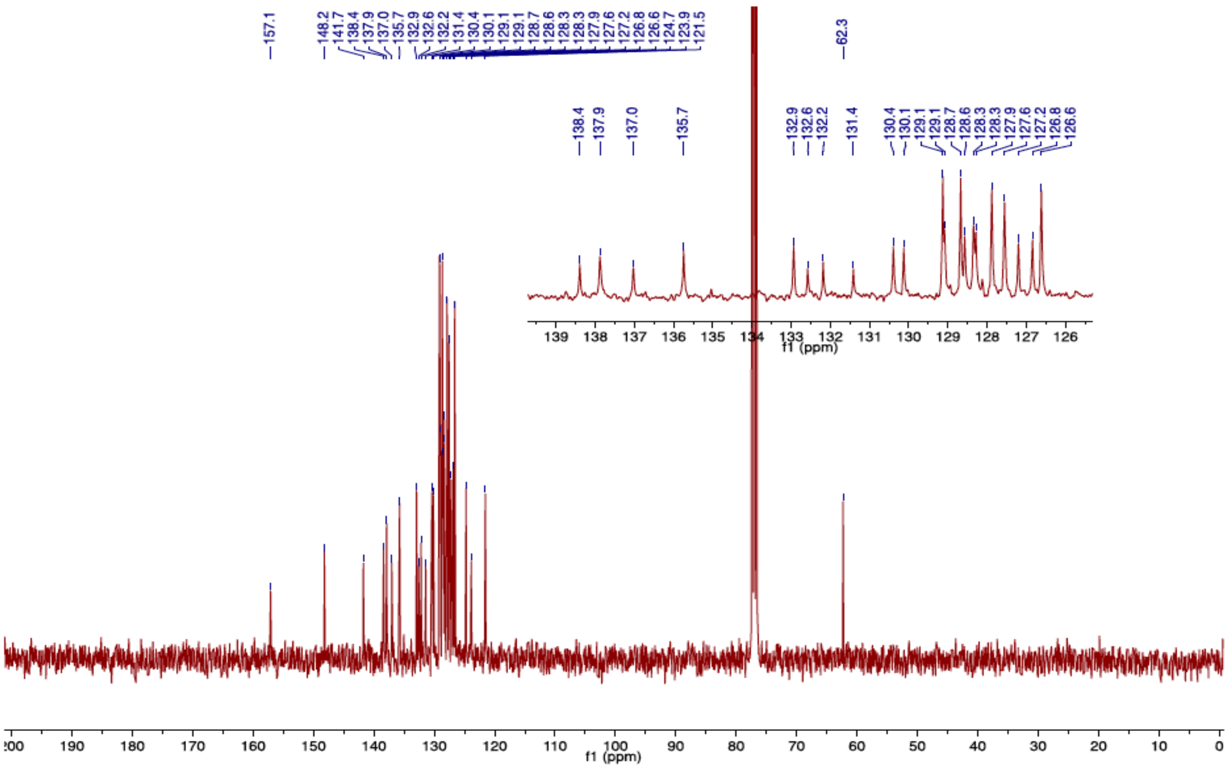

7. X-Ray Crystallographic Data.

Table S1: Crystal data for **(*R*)-3Ac**:

Empirical formula	C27 H18 Cl N	
Formula weight	391.87	
Temperature	100(2) K	
Wavelength	0.56086 Å	
Crystal system	Orthorhombic	
Space group	$P2_12_12$	
Unit cell dimensions	a = 11.4918(5) Å	a= 90°
	b = 21.9320(11) Å	b= 90°
	c = 7.7820(4) Å	g = 90°
Volume	1961.36(16) $Å^3$	
Z	4	
Density (calculated)	1.327 Mg/m^3	
Absorption coefficient	0.115 mm^{-1}	
F(000)	816	
Crystal size	0.300 x 0.210 x 0.090 mm^3	
Theta range for data collection	1.579 to 22.978°.	
Index ranges	-15<=h<=15, -30<=k<=29, -10<=l<=10	
Reflections collected	45240	
Independent reflections	5497 [R(int) = 0.0352]	
Completeness to theta = 19.665°	100.0 %	
Absorption correction	None	
Max. and min. transmission	0.7448 and 0.6751	
Refinement method	Full-matrix least-squares on F^2	
Data / restraints / parameters	5497 / 0 / 262	
Goodness-of-fit on F^2	0.906	
Final R indices [I>2sigma(I)]	R1 = 0.0392, wR2 = 0.1213	
R indices (all data)	R1 = 0.0414, wR2 = 0.1238	
Absolute structure parameter	0.010(19)	
Extinction coefficient	n/a	
Largest diff. peak and hole	0.359 and -0.280 e.$Å^{-3}$	

Figure S1: Thermal ellipsoid plot for **(*R*)-3Ac**:

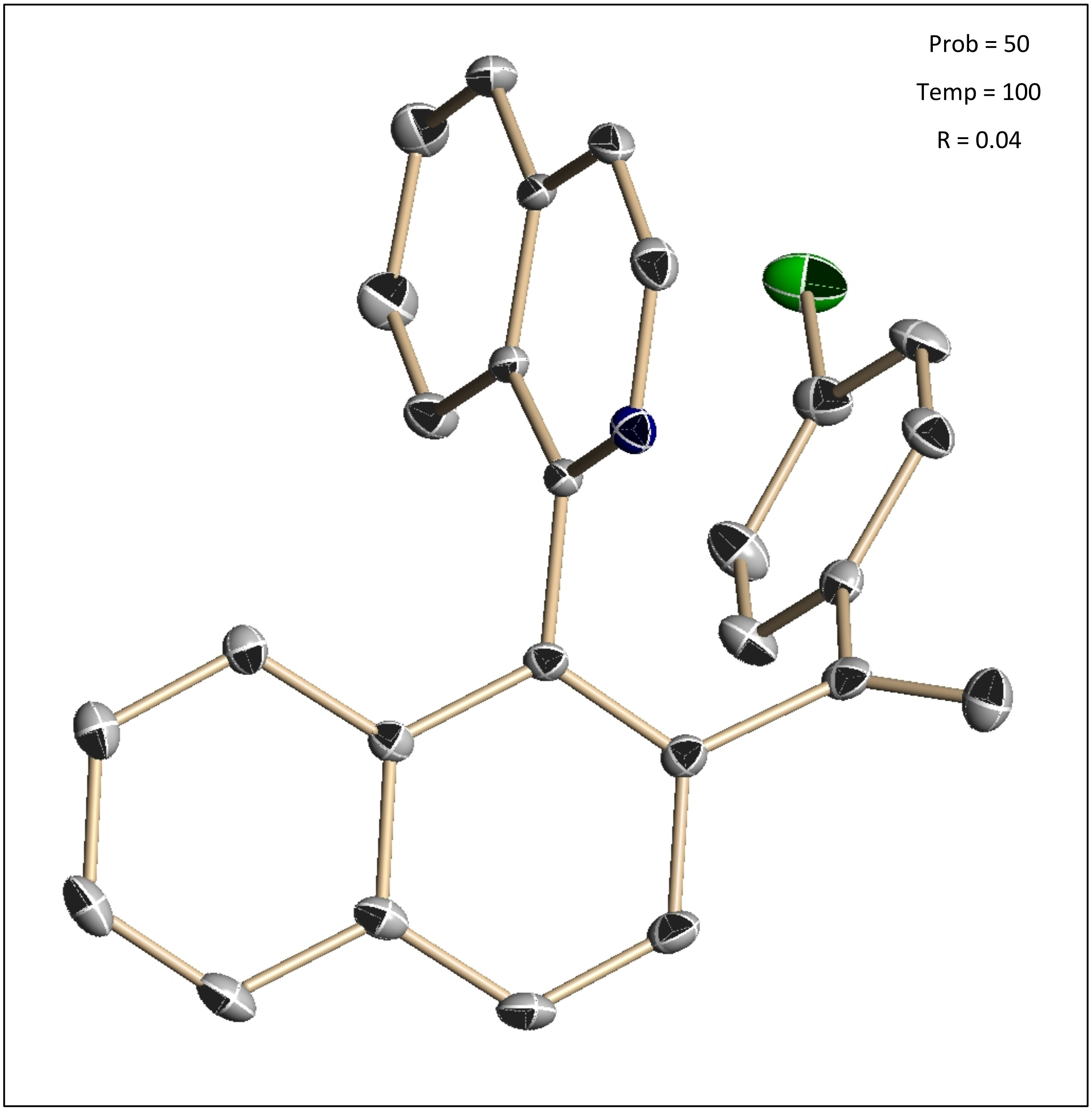

ORTEP drawing of **3Ac** showing ellipsoid contours at 50% probability level.
